# MesenSistem-EB: systemic haploidentical mesenchymal stem cell therapy in recessive dystrophic epidermolysis bullosa associated with clinical benefits and correlated with MCP1 and sCD40L dynamics

**DOI:** 10.3389/fimmu.2026.1789537

**Published:** 2026-05-05

**Authors:** Rocío Maseda, María Carmen Arriba, Lucía Martínez-Santamaría, Eva Jiménez, Sara Herráiz-Gil, Nuria Illera, Lucía Quintana-Castanedo, Marta García, Susana Suárez-Sancho, Rosa Yáñez, Isabel Pérez-Conde, Marta Carretero, Magdalena Martínez-Queipo, Raquel de Paz, Carlos León, Víctor Jiménez-Yuste, Alberto M. Borobia, Ángeles Vicente, Su M. Lwin, John A. McGrath, María Eugenia Fernández-Santos, Nora Butta, Rosa Sacedón, Marcela del Río, Raúl de Lucas, María José Escámez

**Affiliations:** 1 Department of Dermatology, La Paz University Hospital, Madrid, Spain; 2 U714-CIBERER (Centro de Investigación Biomédica en Red de Enfermedades Raras), Madrid, Madrid, Spain; 3 Department of Bioengineering, Universidad Carlos III de Madrid (UC3M), Madrid, Spain; 4 Centro de Investigaciones Energéticas, Medioambientales y Tecnológicas (CIEMAT), Health Research Institute-Fundación Jiménez Díaz University Hospital, Universidad Autónoma de Madrid (IIS-FJD, UAM), Madrid, Spain; 5 Spanish Network of Advanced Therapies by TERAV-ISCIII, Madrid, Spain; 6 Cell Biology Department, Faculty of Medicine, Complutense University, Madrid, Spain; 7 ATMPs Production Unit-GMP Facility, Gregorio Marañón General University Hospital, Instituto de Investigación Sanitaria Gregorio Marañón (IiSGM), Madrid, Spain; 8 Division of Hematopoietic Innovative Therapies, Centro de Investigaciones Energéticas, Medioambientales, y Tecnológicas (CIEMAT)/Centro de Investigación Biomédica en Red de Enfermedades Raras (CIBERER-ISCIII), Instituto de Investigación Sanitaria de la Fundación Jiménez Díaz (IIS-FJD), Madrid, Spain; 9 St John’s Institute of Dermatology, School of Basic and Medical Biosciences, King’s College London, London, United Kingdom; 10 National Institute for Health and Care Research (NIHR) Biomedical Research Centre, Guy’s and St Thomas’ NHS Foundation Trust and King’s College London, London, United Kingdom; 11 IdiPAZ, Hospital La Paz Insititute for Health Research, La Paz University Hospital, Madrid, Spain; 12 Coagulopathies and Haemostasis Disorders Group, IdiPaz, Hematology and Haemotherapy Unit, La Paz University Hospital, Madrid, Spain; 13 Clinical Pharmacology Department, IdiPAZ, La Paz University Hospital, School of Medicine, Autonomous University of Madrid, Madrid, Spain; 14 Stem Cells, Immunity and Cancer Group, Health Research Institute Hospital 12 de Octubre (i+ 12), Madrid, Spain

**Keywords:** bone marrow-derived mesenchymal stem cells, clinical trials, inflammation, mesenchymal stromal cells, recessive dystrophic epidermolysis bullosa, systemic cell therapy

## Abstract

**Background:**

Recessive dystrophic epidermolysis bullosa (RDEB) is a devastating genodermatosis caused by biallelic *COL7A1* mutations, where chronic inflammation drives disabling complications affecting quality of life and survival. Effective inflammatory control remains a major unmet need. While systemic administration of mesenchymal stromal cells (MSC) from various sources has proven safe with transient benefits yet only one recent placebo-controlled study in RDEB. Consistent type VII collagen (C7) deposition is lacking, and therapeutic mechanisms and predictive biomarkers remain undefined. The MesenSistem-EB trial evaluated for the first time the use of haploidentical BM-MSC and extensively explored their anti-inflammatory potential as a monotherapy in RDEB.

**Methods:**

Phase I/II, single-center, open-label trial, in which nine children with severe RDEB were recruited and eight completed the study after three intravenous infusions of haploidentical BM-MSC (2–3×10^6^ cells/kg) at 21-day intervals. Safety, clinical and patient-reported outcomes, systemic inflammatory mediators and peripheral blood immune profiles were assessed over 12 months.

**Results:**

MSC therapy was well-tolerated without serious adverse events. Long-term pruritus, sleep and fatigue were globally reduced. In 7/8 patients at least one key domain significantly improved (disease severity, pruritus, or inflammation) with five classified as good responders. Global median CRP and fibrinogen levels remained stable throughout the study period. Responses correlated with sCD40L and MCP1 dynamics. Flow cytometry revealed altered circulating myeloid and lymphoid compartments at baseline. Post-infusion, immune cell subset changes did not consistently distinguish responders while typically including increased CLA expression on monocytes, partial recovery of memory CD8^+^ T cells and disappearance of an aberrant granulocyte population, potentially linked to immature myeloid mobilization driven by chronic inflammation. Haploidentical MSC did not consistently confer durable engraftment or sustained C7 deposition.

**Conclusions:**

BM-MSC is a safe and potentially effective anti-inflammatory intervention that mitigates the expected escalation of systemic inflammatory markers during a critical phase of RDEB progression. MCP1 and sCD40L modulation, with both potentially serving as predictive biomarkers. MSC appear to exert both shared and patient-specific immunomodulatory effects depending on baseline inflammatory cues. Findings provide insights into individual variability, underlying mechanisms and potential therapeutic responsiveness, supporting their use also as a complementary strategy.

**Clinical trial registration:**

## 1 Introduction

Recessive dystrophic epidermolysis bullosa (RDEB) is a monogenic skin disorder with multi-organ involvement and systemic inflammatory implications (1). It is caused by biallelic loss-of-function variants in *COL7A1* leading to the partial or complete absence of collagen VII (C7), key component of anchoring fibrils (AF) attaching the epithelia to the underlying connective tissue (1). Clinically, this defect manifests as mucocutaneous fragility and recurrent blistering, often resulting in non-healing wounds and chronic local inflammation. Bacterial colonization and infections may exacerbate inflammation and wound healing problems, potentially triggering both innate and adaptive immune responses in RDEB (2–6). Extensive damage of skin (>20-30% body surface area) and mucosae over prolonged periods might eventually turn localized inflammation to a systemic level (3, 7). Consequently, patients with RDEB often exhibit leukocytosis, elevated systemic levels of C-reactive protein (CRP), pro-inflammatory cytokines and profibrotic factors such as TGFβ, HMGB1, MCP1 and periostin (3, 8–13). Recent longitudinal data indicates that severe RDEB is characterized by an early and sustained systemic inflammatory profile, with markers progressively increasing from early childhood before plateauing in mid-adulthood (14).This chronic inflammatory scenario is closely intertwined with severe outcomes such as fibrosis, mitten deformities and an increased risk of metastatic squamous cell carcinoma (15–17). Severe mucosal involvement can compromise feeding, ultimately contributing to malnutrition and anemia (18, 19). Additionally, peripheral neuropathy associated with inflammation may underpin chronic pruritus and pain (20–22). Thus, the systemic complications of RDEB, driven by chronic inflammation, significantly impact both quality of life and life expectancy, while also posing a substantial financial burden (23, 24). The effective control of inflammation remains a major unmet need in RDEB management.

RDEB is a prototypical disease targeted in numerous clinical trials exploring advanced therapies (25), leading to the authorization of two gene therapies. Vyjuvek (FDA 2023, EMA 2025) (26) provides transient *in vivo* correction of *COL7A1* using non-integrative vectors, while Zevaskyn (FDA 2025) (27) employs skin grafts with integrative vectors for a permanent effect. However, given their recent approval, real-world data regarding their long-term benefits, accessibility and cost are still lacking (28). Although these corrective therapies represent important clinical milestones, they do not address systemic manifestations, making complementary systemic approaches a pressing need for controlling inflammation and managing secondary complications. In this field, the discovery that mesenchymal stem cells (MSC) played a role in tissue regeneration by modulating inflammation transformed the landscape of stem cell therapy for inflammatory diseases (29). Clinical trials involving systemic administration of allogeneic bone marrow MSC (BM-MSC) have reported minimal side effects, with no major allo-sensitization issues in RDEB (30–33). These CT have shown improvement in wound healing, alleviation of pain and itch, better sleep and enhanced quality of life, despite considerable variability in individual responses. However, no new C7 or AF formation has been generally observed in the recipients’ skin. The optimal dose and infusion schedule for MSC, as well as biomarkers to predict favorable responses, remain undetermined. Finally, the anti-inflammatory and immunomodulatory effects of MSC in RDEB are thought to be primarily mediated through paracrine mechanisms that are poorly characterized. These challenges underscore the need for further research to optimize MSC therapy in RDEB.

MesenSistem-EB was developed to improve the therapeutic sustainability of BM-MSC by using haploidentical donors (differing at a single locus) with the goal of enhancing cell tolerance, persistence and graft success. The rationale for this approach relied on studies suggesting that host immune responses may gradually clear allogeneic MSC, potentially reducing their therapeutic efficacy (34). Despite the limitations of an uncontrolled, single-arm design with a small sample size MesenSistem-EB provides additional evidence on the safety and potential clinical benefits of MSC, elucidates their anti-inflammatory mechanisms, sheds light on patient response variability and, for the first time, investigates the use of haploidentical cells.

## 2 Materials and methods

### 2.1 Study design and approval

MesenSistem-EB (*Safety study and preliminary efficacy of infusion haploidentical mesenchymal stem cells derived from bone marrow for treating recessive dystrophic epidermolysis bullosa)* is a single-center, single-group, open-label, phase I/II pilot clinical trial designed to evaluate the safety and preliminary efficacy of systemic haploidentical BM-MSC in children with severe RDEB. The study was conducted at a Spanish reference unit of EB (Hospital Universitario La Paz, HULP) in collaboration with the Academia (Universidad Carlos III de Madrid and Universidad Complutense de Madrid) and another European reference group for EB (St John’s Institute of Dermatology - King College of London).

**Figure 1:**
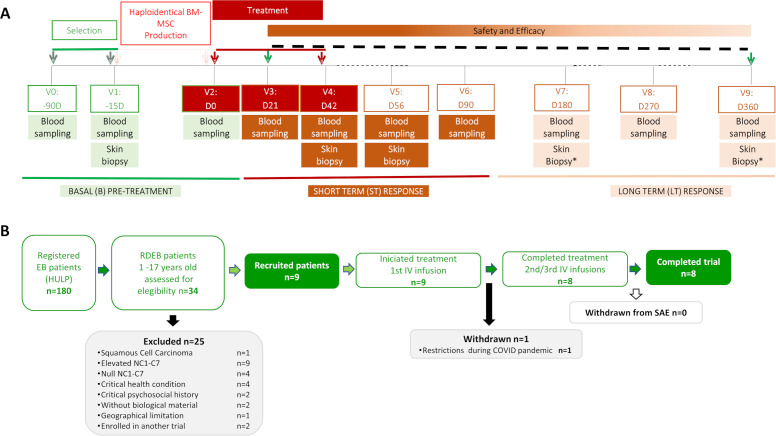
MesenSistem-EB study summary. **(A)** Trial design and sampling Schedule. **(B)** Flowchart illustrating participant recruitment, retention, exclusion and withdrawal. D, days; V, visit.

The schedule consisted of 10 visits, including three screening/basal visits covering the three months pre-treatment (V0–V2; day (D) –90 to 0), three treatment visits every 21 days (V2–V4; day 0, 21, 42) and 1-year post-treatment safety and efficacy follow-up (V5–V9; day 60 to 360) (**Figure 1A**; **Supplementary Table 1**). Safety was also assessed by phone eight days (± 2) after each infusion. Efficacy was evaluated by grouping visits into short-term (ST: V3–V6; day 21–90) and long-term (LT: V7–V9; day 180–360) responses relative to basal status (V0–V2; day –90 to 0). Both safety and efficacy were assessed individually and globally as a group. A multi-omics study (Omic-MesenSistemEB) was conducted, the results of which will be reported separately.

MesenSistem-EB was approved by the Spanish Agency of Medicines and Medical Devices (AEMPS, EudraCT 2017-000606-37) and it was also registered at ClinicalTrials.gov (NCT04153630). The study was performed in agreement with Declaration of Helsinki principles and subsequent revisions (Fortaleza, Brazil, October 2013), after favorable reports from the Ethics Committee at Hospital Universitario La Paz (HULP Code: PI-3439, PI-1359 and PI-1595). Written informed consent was obtained from all individuals (RDEB patients, unaffected children and donors) with the understanding that the collected information including clinical photographs may be published. The unaffected group did not serve as a control arm of the clinical trial, but as a reference group for certain specific parameters, as properly specified.

### 2.2 Recruitment

Patients and haploidentical donors were recruited over 18 months (**Figure 1B**). Children (age 1–17 years) registered at HULP with clinical and molecular diagnosis of RDEB and a minimum cutaneous expression of the non-collagenous domain of C7 (NC1-C7) were eligible. Critical condition (i.e., systemic infection, very low albumin or hemoglobin values), presence of anti-basement membrane zone (α-BMZ) IgG and IgA deposits and history/signs of malignancy were main exclusion criteria (**Table 1**). Five females and three males were included based on these clinical and molecular criteria. Sex was not considered a biological variable, as no related differences have been observed in RDEB (1, 3). EB molecular diagnosis was made by immunofluorescence antigenic immunomapping and *COL7A1* genotyping as recommended (35).

**Table 1:** Criteria compliance for recruitment of RDEB patients in MesenSistemEB.

Main inclusion criteria							Main exclusion criteria			
Code	Sex	Aged 1-17	BEBSS	Biallelic COL7A1 gene pathogenic variants	Minimal C7 skin expression		α-BMZ autoantibodies	Critical biochemistry		Malignancy signs/history
		Years old	Score > 20	Paternal maternal	NC1-C7 IIF	NC1-C7 WB	IgA & IgG IIF	ALB <2.5 g/dL	Hb < 7.5 g/dL	
P1	F	4	41.75	c.6527insC; p.Gly2177Trpfs113*(ex80) c.2044C/T; p.Arg682*(ex15)	Yes	Yes	Negative	3.4	9.7	No
P2	F	10	48.00	c.6527insC; p.Gly2177Trpfs113*(ex80) c.6527insC; p.Gly2177Trpfs113*(ex80)	Yes	Yes	Negative	3.6	10.0	No
P3	M	12	40.00	c.6527insC; p.Gly2177Trpfs113*(ex80) c.6527insC; p.Gly2177Trpfs113*(ex80)	No	Yes	Negative	3.7	10.4	No
P4	F	14	30.00	c.6527insC; p.Gly2177Trpfs113*(ex80) c.6527insC; p.Gly2177Trpfs113*(ex80)	Yes	No	Negative	3.7	11.3	No
P5	M	17	51.75	c.6527insC; p.Gly2177Trpfs113*(ex80) c.6040delAG; p.Gln2014Argfs84* (ex73)	No	Yes	Negative	3.3	8.0	No
P6	F	6	27.00	c.7249C/T; p.Gln2417* (ex94) c.6527insC; p.Gly2177Trpfs113*(ex80)	Yes	Yes	Negative	4.0	10.1	No
P7	F	8	37.50	c.6527insC; p.Gly2177Trpfs113*(ex80) c.6527insC; p.Gly2177Trpfs113*(ex80)	Yes	Yes	Negative	3.8	11.2	No
P8	F	4	39.25	c.6527insC; p.Gly2177Trpfs113*(ex80) c.5561delGGGAG; p.Gly1854Glufs*16 (ex65)	Yes	Yes	Negative	4.1	10.3	No
P9	M	4	23.63	c.6527insC; p.Gly2177Trpfs113*(ex80) c.6527insC; p.Gly2177Trpfs113*(ex80)	Yes	Yes	Negative	4.0	11.0	No

ALB, Albumin; BMZ, Basal membrane zone; C7, Type VII collagen; Hb, Hemoglobin; ex, exon; IIF, Indirect immunofluorescence; NC1, Non-collagenous domain 1 (amino-terminal); WB, Western blot from primary culture keratinocyte with the exception of P7 (fibroblasts). Recruitment criteria are fully described on ClinicalTrials.gov (NCT04153630) and in the EU Clinical Trials Register (EudraCT 2017-000606-37).

Progenitors willing to donate BM were also recruited, prioritizing sex-mismatch to allow for subsequent donor chimerism assessment via XY-FISH. Their health status and medical history were evaluated and peripheral blood samples were obtained for human leucocyte antigen (HLA) genotyping and infectious disease testing (**Supplementary Table 2**). All nine eligible patients, unaffected children (**Supplementary Table 3**) and their progenitors accepted to participate.

### 2.3 Haploidentical BM-MSC procurement and production

The donation, procurement and donor testing followed Good Clinical and Manufacturing Practices (directive 2004/23/EC, 2006/17/EC and 2006/86/EC). Briefly, BM-MSC were obtained by aspiration from the iliac crest of haploidentical donors (progenitor, sex mismatch). The investigational medicinal product (IMP) was manufactured according to advanced therapy medicinal product (ATMP) guidelines and regulations (directive 2004/23/EC, 2006/17/EC and 2006/86/EC) at the GMP-facility of UPC-FIBHGM (Unidad de Producción Celular de la Fundación para la Investigación Biomédica del Hospital Gregorio Marañón; certificate No. ES/040I/24) which is authorized to produce BM-MSC for RDEB (IMP No.12-088). Briefly, bone marrow aspirates were registered and immediately processed upon arrival at UPC-FIBHGM. Mononuclear cells were isolated from 100–150 mL of aspirate by Ficoll density gradient centrifugation, then cultured and expanded ex vivo under controlled conditions for up to four passages and a maximum of six weeks. Once 70-80% of confluence and the required total dose was reached, BM-MSC were cryopreserved to maintain optimal viability. During expansion, supernatants were collected for sterility and *Mycoplasma* testing and cellular samples were analyzed for phenotype (via flow cytometry), genetic stability (via CGH array) and potency. Flow cytometry confirmed the immunophenotype, reliably identifying the active ingredient, while genetic stability testing ensured that MSC had not accumulated clonal amplifications or deletions, ruling out any genomic alterations in the donor bone marrow that might increase transformation risk. BM-MSC trilineage differentiation assays were verified.

### 2.4 Haploidentical BM-MSC characterization

Characterization of BM-MSC followed recommendations from the International Society of Cellular Therapy ISTC (36). For adipogenic differentiation, Poietics™ Human Mesenchymal Stem Cells Adipogenic Differentiation Medium (Lonza) was used according to the manufacturer’s protocol. Briefly, 190,000 cells were resuspended in 2.5 mL of complete culture medium and seeded into a T25 flask, then incubated at 37 °C, 5% CO_2_ and 90% humidity. Media changes were performed every three–four days until approximately 90% confluence was reached, at which point differentiation was induced. Three cycles of three days in adipogenic induction medium followed by one–three days in maintenance medium were completed. After these cycles, two additional maintenance medium changes were performed every three–four days. Differentiated cells were then fixed and stained with Oil Red O (Sigma Aldrich) and hematoxylin (Sigma Aldrich) for the presence of lipid vacuoles. Osteogenic differentiation was conducted using HyClone AdvanceSTEM™ Osteogenic Differentiation Medium. Forty thousand cells were resuspended in 2.5 mL of complete medium, seeded into a T25 flask, and incubated at 37 °C, 5% CO_2_ and 90% humidity. After cell attachment (day 1), cells were treated with 2.5 mL of osteogenic differentiation medium, with media changes every three–four days for two–three weeks. Morphological changes, including delamination and calcium deposits, were observed. Cells were then fixed and stained with Alizarin Red (Sigma Aldrich) and hematoxylin (Sigma Aldrich) to visualize calcium deposits. Chondrogenic differentiation was performed using NH ChondroDiff Medium (Miltenyi Biotec). Briefly, an 8,000-cell pellet was prepared by centrifuging at 300 g for 10 minutes to form a 5 μL droplet, which was then placed in the center of a well in a 4-well plate, with water in adjacent wells to maintain humidity. After a 1-hour incubation at 37 °C with 5% CO_2_ and 90% humidity, 0.5 mL of chondrogenic differentiation medium was added, with media changes every three–four days. After 14 days, cells were fixed and stained with Toluidine Blue (Sigma Aldrich) to detect proteoglycan synthesis, a characteristic of chondrogenic differentiation.

BM-MSC immunosuppressive potential and secretory profiles were assessed for six of the eight haploidentical donors (MSC remnants from the other two donors were unavailable) in co-culture assays with activated human peripheral blood mononuclear cells (PBMNCs). PBMNCs were obtained by Ficoll-Paque PLUS (GE Healthcare Bioscience, Uppsala, Sweden) density gradient from three healthy unrelated buffy coat donors provided by the Centro de Transfusión de la Comunidad de Madrid (CTCM). PBMNCs were stained with the intracellular fluorescent dye, carboxyfluorescein diacetate succinimidy ester (CellTrace™ CFSE Cell Proliferation Kit; Molecular Probe/Invitrogen, USA). Before co-culture, BM-MSC were plated in 24-well plates. Twenty-four hours later, stained PBMNCs were added to each well in the presence of 200 ng/ml of monoclonal antibody anti-human CD3 (Clone OKT3; Biolegend, San Diego, USA) and 150 U/ml of human IL2 (Peprotech, London, United Kingdom) to induce the specific proliferation of T lymphocytes. BM-MSC and activated PBMNC were co-cultured at three different MSC ratios: 1:5, 1:20 and 1:50. Co-cultures were incubated at 37 °C with 5% CO_2_ for 72 hours. After three days of incubation, cells harvested from culture wells were analyzed by flow cytometry for T cell proliferation. Data were analyzed with ModFit LT™ (Verity Software House, Topsham, ME, USA). The negative control, non-activated PBMNC, was used to define the activation threshold of non-proliferating T cells. After PBMNC activation, T-cell proliferation percentages following BM-MSC coculture were determined using this threshold. All values were expressed as a percentage of the respective positive control. T-cell proliferation suppression was calculated as: 100% - [T-cell proliferation after coculture (% of positive control)].

In addition, supernatants from BM-MSC, PBMNC (prior and after activation) and co-cultures (BM-MSC and activated PBMNC) were collected at the end of the incubation period to quantify cytokines associated with immune response (CCL2, IFNγ, IL4, IL6, IL8, IL10, PGE2, TFGβ, TNFα and VEGF), by flow cytometry using the LEGENDplex™ Human Essential Immune Response Panel (Biolegend San Diego, USA). PGE2 was quantified by PARAMETER™ Prostaglandin E2 Assay (R&D Systems, Minneapolis, USA) and VEGF was quantified by Quantikine^®^ ELISA Human VEGF Immunoassay (R&D Systems, Minneapolis, USA).

General specifications, formulation and quality controls of BM-MSC are outlined in **Supplementary Table 4**, while batch-specific properties (HP1–HP9) are detailed in **Supplementary Table 5**.

### 2.5 Intervention

A single group assignment interventional model was followed. A total of three intravenous (IV) infusions of BM-MSC (2-3 × 10^6^ cells/kg/infusion) derived from haploidentical donors were administered every 21 days (Day 0, 21 and 42; visits V2-V4). The frequency, previously used by our group (5) and the dose are supported by existing data demonstrating safety and potential clinical benefits in patients with RDEB and other conditions, even at higher doses, using weekly, biweekly and monthly schedules (30–33, 37, 38). Delivery dates and doses can be found in **Supplementary Table 6**. A 30 ml BM-MSC-suspension was infused via a peripheral cannula during 5–10 minutes (≥10 mL/min). Prior to infusion (30 minutes), the patients received 5 mg of dexchlorpheniramine, 100 mg of hydrocortisone and 500 mg of paracetamol. After infusion, patient vital signs were monitored for at least four hours prior to discharge.

### 2.6 Safety assessment

The primary safety outcomes included the incidence of treatment-emergent adverse events (AE) observed over the 1-year follow-up period. Hepatic and renal toxicity, vital signs, global coagulation and the expression of antibodies against C7 were also evaluated.

Hepatic function was evaluated through the levels of alanine aminotransferase (ALT), aspartate aminotransferase (AST), γ-glutamyl transferase (GGT), alkaline phosphatase (ALP), total proteins, total bilirubin and albumin. Renal function was monitored by determining urea and creatinine levels.

Patients were closely monitored for clinical signs of thromboembolic events by rotational thromboelastometry (ROTEM^®^, Pentapharm, Munich, Germany). ROTEM evaluates the kinetics of clot formation and fibrinolysis in blood samples drawn with 3.2% sodium citrate as anticoagulant. Coagulation was triggered by the exTEM and fibTEM reagents (Werfen, Madrid, Spain). The following parameters were recorded: clotting time (time from the start of clot formation until an amplitude of 2 mm, in seconds); alpha angle (α) (the slope of the tangent line to the clotting curve through the 20 mm amplitude that reflects the rate of fibrin polymerization, in degrees); the clot firmness X min after clotting time; the maximal clot firmness (MCF, in mm); and clot lysis as the percentage of clot lysed after 60 min. Plasma fibrinogen levels were measured by Clauss method, along with platelet count to assess the patient’s coagulation status and potential for thrombosis.

Circulating anti-C7 autoantibodies were measured by ELISA in the patient’s serum, while indirect immunofluorescence (IIF) microscopy for IgA and IgG against BMZ autoantibodies was performed on 4-μm sections of human skin, salt-split skin and monkey esophagus substrates. Direct immunofluorescence microscopy was also conducted for IgM, IgG, IgA, C3 and fibrinogen deposition. These studies were carried out at the Immunodermatology Laboratory at St John’s Institute of Dermatology (London, UK).

### 2.7 Efficacy assessment

Secondary efficacy outcomes grouped into four categories were measured: 1) muco-cutaneous response: percentage of skin surface affected, EB disease severity (specific scales for EB), skin resistance and presence of both, C7 and anchoring fibrils as well as dermal chimerism; 2) systemic inflammation response: circulating levels of acute phase reactants [C reactive protein (CRP), prealbumin, fibrinogen and retinol binding protein (RBP)], cytokines and immunomodulators peripheral blood leukocyte count and flow cytometry analysis of immune cell populations; 3) pain and 4) pruritus, using specific scales. Finally, the impact of the treatment on the health-related quality of life (HRQOL) was assessed by the Pediatric Quality of Life Inventory™ (PedsQL™ 4.0). Patients were categorized based on an exploratory multidimensional assessment to account for inter-individual variability according to the statistical significance of clinical parameter shifts (See Statistical Analysis).

Reference values for standard biochemical parameters in the age-matched general population recorded from HULP were used. Control values for serum cytokines and immunophenotyping of peripheral blood cells were obtained from unaffected children with ages in the same range that the RDEB patients.

### 2.8 EB severity scores

Clinical muco-cutaneous involvement was assessed by the Epidermolysis Bullosa Disease Activity and Scarring Index (EBDASI total = EBDASI activity + EBDASI damage) (39) and the Birmingham Epidermolysis Bullosa Severity Score (BEBSS) (40). EBDASI quantifies the overall severity of involvement of the skin, scalp, mucous membranes, nails and other epithelialized surfaces in terms of activity (0–276 points) and damage (0–230 points). The EBDASI total score ranges from 0 to 506, with phenotype severity classified as mild (0–42), moderate (43–106), or severe (107–506). BEBSS quantifies disease severity across 11 elements, including the percentage of body surface area (BSA) affected, nail involvement, oral, ocular, laryngeal and esophageal complications, hand scarring, skin cancer, chronic wounds, alopecia and nutritional status. BEBSS total score ranges from 0 to 100. All evaluations were conducted by a single dermatologist.

Pain was monitored at each visit using two scales. Visual Analog Scale (VAS) (41) from 0 (absence of pain) to 10 (unbearable pain): 1–3 mild pain, 4–6 moderate pain, 7–9 severe pain. The Wong-Baker FACES^®^ scale (42) represents pain graphically with six faces and numerically with even values, where 0 indicates no pain and 10 represents the worst imaginable pain.

Changes in itch were assessed at each visit also using two scales. The Leuven Itch Scale 1.0 (LIS 1.0) (43, 44) evaluates multiple dimensions of itch, including frequency, duration, severity, distress, consequences and surfaces. Responses are transformed into a score ranging from 0 to 100, with 100 indicating the most severe condition. The Itch man Scale (45) graphically represents five levels, where 0 indicates comfort and 4 represents unbearable itch that hinders concentration.

The PedsQL™ Measurement Model (46) includes self-report versions for young children (ages 5–7), children (ages 8–12) and teens (ages 13–18), as well as parent proxy versions for all age groups, including toddlers (ages 2–4). In this study, three modules have been assessed before (V0, V1) and after the treatment (V4, V5, V6, V7, V8 and V9): the Pediatric Quality of Life Inventory™ (PedsQL™ 4.0, parent and child forms), the Multidimensional Fatigue Scale (PedsQLTM, parent and child forms) and the PedsQLTM 2.0 Family Impact Module (parent forms). The Total Scale Score is the sum of all items divided by the number of items answered. These forms evaluate the extent to which each item has been a problem using a 5-point scale (0 = never a problem to 4 = almost always a problem). The items are reverse scored and converted to a 0–100 scale, with higher scores indicating better HRQOL, lower problems related to fatigue and better functioning regarding family impact.

### 2.9 Laboratory procedures

EB molecular diagnosis, retrieved from the EB Registry at HULP, was made by immunofluorescence antigenic immunomapping and sequencing of *COL7A1* as recommended (35). Presence of NC1-C7 domain (LH7.2 monoclonal antibody; Sigma-Aldrich) at the dermo-epidermal junction (DEJ) was determined by indirect immunofluorescence (IIF) on 5-μm cryosections from patient’ skin biopsies, as previously described (47). The percentage of NC1-C7 expression (compared with the control) was calculated from the corrected total fluorescence (CTCF; mean +/- SD). To ensure comparability, all samples were processed in a single batch, and images were captured using identical exposure times and settings. CTCF = Integrated Density – (Selected area at DEJ x Mean fluorescence of background readings) quantified in five random fields per sample by ImageJ (https://imagej.nih.gov/ij/index.html). During the selection period NC1-C7 expression was also assessed in cell lysates from primary keratinocytes/fibroblasts by western blot (WB; SDS-PAGE) as formerly described elsewhere (13). LH7.2 policlonal antibody for WB was a gift from A. Nyström (University of Freiburg-Germany).

Presence of donor MSC in the skin (dermal chimerism) of treated patients was analyzed by FISH. FISH was performed on skin histological sections (FFBE or frozen; 5 µm) at D180 post-treatment. Interphase nuclei with centromeric probes of the X [SE X (DXZ1) Xp11.1-q11.1_green] and the Y [SE Y (DYZ3) Yp11.1-q11.1_red] chromosomes from MetaSystems and Kreatech Diagnostics were respectively used.

Circulating levels of cytokines and immunomodulators were determined in serum obtained from whole blood collected with silica particles and serum separator gel (STT) -after 30 minutes at room temperature- by refrigerated centrifugation at 3000 ×g for 15 minutes. IFNγ, IL1β, IL2, IL4, IL6, IL10, IL13, IL15, IL17A, monocyte chemoattractant protein 1 (MCP1), soluble CD40 ligand (sCD40L), TNFα, VEGFα and fractalkine were measured with MILLIPLEX MAP Human Cytokine/Chemokine Magnetic Bead Panel (HCYTOMAG-60K, Merck, Madrid, Spain). ELISA was used to measure total and active TGFβ (R&D Systems^®^, Inc., USA), bioactive hepcidin (^©^DRG Instruments GMBH, Deutschland), erythropoietin (EPO; R&D Systems^®^, Inc., USA) and high mobility group box 1 (HMGB1; Mybiosource, Netherland).

Peripheral blood lymphocytes (PBL) populations analysis by flow cytometry was performed immediately after harvesting EDTA-whole blood samples. Following FC receptor blocking (FcR blocking reagent, Miltenyi), samples were incubated with specific fluorochrome-conjugated (FITC, PE, PerCP-Cy5.5, Pe-Cy5.5 Alexa Fluor 647 or APC) antibodies for cell surface markers: CD3 (HIT3a), CD4 (OKT4), CD8 (SK1), CD11b (ICRF44), CD11c (3.9), CD13 (WM15), CD14 (14PC-100T and 47-3D6), CD15 (HI98), CD16 (VEP13), CD19 (HIB19), CD33 (WM-53), CD45 (2D1), CD45RA (HI100), CD45RO (UCHL1), CD56 (AF12-7H3 and 5.1 H11), CD62L (DREG-56), CD90 (5E10), CCR2 (K036C2), CCR10 (6588–5), CLA (HECA-452), CXCR3 (G025H7), CX3CR1 (2A9-1), CCR4 (L291H4), CXCR4 (12G5), HLADR (GRB-1) and TC receptors TCR γδ (B1) and TCRαβ (IP26). Antibodies were obtained from ImmunoStep (Salamanca, Spain), BioLegend, Myltenyi and BD Biosciences (Madrid, Spain). Antibodies were obtained from ImmunoStep (Salamanca, Spain), BioLegend, Myltenyi and BD Biosciences (Madrid, Spain). Then, erythrocyte cell lysis was performed with BD PharmLyse™ (BD Biosciences) and cells were fixed using BD CellFIX buffer. All analyses were conducted the Centre for Cytometry and Fluorescence Microscopy (Universidad Complutense de Madrid, Spain) in a FACS Calibur flow cytometer (BD Biosciences).

### 2.10 Statistical analysis

Statistical analysis was performed with GraphPad Prism 10.4.1 software. The Shapiro-Wilk test indicated non-parametric distribution in most of the data. Therefore, medians and interquartile ranges (IQRs: Q1, 25^th^ percentile and Q3, 75^th^ percentile) were used for the descriptive statistics and graphical representations. Non-representative outliers (1.5 IQR: below Q1 or above Q3) were removed (48) from the control group of unaffected children within the same age range. Median changes from basal status, were tested against the null hypothesis using 1-sided non-parametric tests: Wilcoxon and Mann Whitney U tests for global (paired data) and individual (unpaired data) analysis, respectively. 95% confidence interval (CI) was used to determine statistical significance for differences between medians in individual analyses. This one-sided approach was based on the expected immunomodulatory effect of BM-MSCs and the progressive natural history of RDEB, prioritizing the detection of clinical signals in an ultra-rare disease cohort while minimizing Type II error (n=8).

Patients were exploratorily classified as good responders when statistically significant changes were observed in two or more clinical parameters, while mild responders were defined by significant improvement in a single key parameter. To determine potential associations between clinical benefit after treatment and significant variation in cytokine levels, 2x2 contingency tables were constructed. The statistical significance of the association was established by applying the 2-sided Fisher’s exact test; p-values ≤ 0.05 were considered statistically significant.

The immunosuppressive potential and the secretory profile of cytokine concentrations in the supernatants were also analyzed for significant statistical differences between the seven haploidentical donors using GraphPad Prism 10.4.1. Differences between medians were calculated using the non-parametric Kruskal-Wallis test, followed by uncorrected Dunn’s pairwise multiple-comparison test. p-values ≤0.05 were considered statistically significant.

### 2.11 Supplementary material summary

The Supplementary material provides additional experimental data, methodological details and extended clinical analyses supporting the findings of the study. **Supplementary Figures 1**–**7** include characterization of haploidentical BM-MSC batches, their immunosuppressive and secretory profiles, coagulation studies, individual C7 expression analyses and comprehensive flow cytometry assessment of myeloid and lymphoid cell populations at baseline and post-treatment. **Supplementary Tables 1**-**21** contain detailed information on study procedures, donor recruitment criteria, MSC product specifications and batch release data, patient demographics and clinical features, safety assessments, autoantibody profiling and full analyses of clinical, nutritional, pruritus and quality-of-life outcomes. An overview of clinical trials using MSC-based therapies for RDEB is also provided.

## 3 Results

### 3.1 Protocol adherence

A summary of the study is provided in **Figure 1**. A total of 34 patients with molecular diagnoses of RDEB registered at the EB National Reference Unit database at HULP (49) were assessed for eligibility between May 2018 and March 2019. Of these, 25 patients did not meet MesenSistem-EB inclusion criteria (**Figure 1B**). All nine eligible pediatric patients, along with their nine haploidentical donors (three out of six matching HLA loci, **Supplementary Table 2**), accepted the invitation to participate and provided informed consent. Patients were treated sequentially, with intervals between treatments ranging from 10 days to 3 months. Eight patients received three repeated intravenous haploidentical BM-MSC and completed the study. One patient (P9) withdrew after the first injection due to COVID-19 health restrictions in Spain. Consequently, data collected from P9 were not included in the analysis but basal status information is provided. Patient P3 received doses below the established threshold (<2 × 10^6^ cells per kg body weight) in all three infusions. Patients P4 and P7 also received lower dose in the second and third infusion, respectively (1.9 million cells per kg body weight) due to unexpected weight gain. The rest of the patients received three infusions of 2-3 × 10^6^ cells per kg body weight, as shown in **Supplementary Table 6**.

### 3.2 BM-MSC characterization

In this study, BM-MSC batches from haploidentical donors (progenitors, HP1-HP9) were obtained fulfilling the minimal standard criteria for multipotent mesenchymal stromal cells stablished by the ISTC (36). BM-MSC were characterized according to their surface antigen expression, trilineage differentiation and immunosuppressive capacity. All BM-MSC batches exhibited the expected immunophenotype (**Supplementary Table 4****),** adhered to plastic and demonstrated *in vitro* differentiation into osteocytes, adipocytes and chondrocytes (**Supplementary Figure 1A****).** The differentiation potential was generally consistent across batches. HP3 and HP8 exhibited adipogenic and osteogenic potential under 50%, respectively (**Supplementary Figure 1B**).

To further assess consistency among batches, the immunosuppressive capacity and secretory profile of BM-MSC from six progenitors were analyzed by co-culturing with activated PBMNC (peripheral blood mononuclear cells) from three healthy buffy coat donors. Samples from the other progenitors were unavailable. All six batches showed statistically significant dose-dependent inhibition of T-cell proliferation in co-cultures at all analyzed ratios (Kruskal-Wallis, p=0.0001) as shown in **Supplementary Figure 1C** (BM-MSC: PBMNC = 1:5 ratio). All batches effectively immunosuppressed activated PBMNC (**Supplementary Figure 1D**) with a median percentage of 79.9% (IQR: 75.5-88.1). Notably, HP1 was the most effective reaching 98.2% of immunosuppression. Overall, cytokine profiling upon co-culture with the different BM-MSC batches revealed an increase in IL4, IL6, IL10, TGFβ, MCP1 and VEGFα, while TNFα and IFNγ were decreased (**Supplementary Figure 2**) suggesting a shift toward an anti-inflammatory and pro-regenerative environment. PGE2 and IL8 displayed the greatest variability, with some BM-MSC batches showing increased levels while others showed decreased levels (**Supplementary Figure 2D**).

### 3.3 Patient characteristics at baseline

The clinical and molecular features of the recruited patients are summarized in **Table 1**. Of the nine unrelated children with RDEB in the cohort, three were males and six were females, aged 4 to 17 years at recruitment. All harbored biallelic null mutations in *COL7A1*, with at least one allele carrying the prevalent Spanish founder mutation, c.6527insC (p.Gly2177Trpfs113*) in exon 80 (47, 50). All patients had BEBSS scores >20 and showed minimal NC1-C7 expression in skin by indirect immunofluorescence (IIF) and/or Western blot. IIF for α-BMZ autoantibodies (IgA and IgG) was negative in all cases. No relevant biochemical abnormalities or history of malignancy were noted at basal status.

All participants had a history of skin fragility and blistering since birth. At study entry, all showed severe mucocutaneous involvement, as defined by EBDASI total scores >107. Despite similar *COL7A1* mutations and NC1-C7 expression levels at the dermo-epidermal junction (DEJ), clinical heterogeneity was observed in mucocutaneous symptoms, pain, pruritus and systemic markers of inflammation and nutrition (**Supplementary Table 7****).**

### 3.4 Safety outcomes

Haploidentical BM-MSC infusions were well tolerated with no serious adverse events (SAE). All patients experienced at least one adverse events (AE), 97.6% of which were mild and resolved within 24 hours (**Supplementary Table 8**). Only 3.9% of AE were related to MSC infusion. Fever and low-grade fever were the most common AE, observed in 62.5% and 87.5% of patients, respectively (**Supplementary Table 9**). Headache was the most frequent unexpected AE, affecting 14.8% of the cohort. No signs of malignancy were present before or within the 1-year follow-up.

No clinically relevant changes in vital signs or hepatic/renal function were observed (**Supplementary Table 10**) except for P3, who showed a transient increase in GGT (from 56 IU/L at baseline to 108 IU/L post-infusion; reference range <73 IU/L) while other liver markers remained within normal limits.

No thrombotic events occurred. Rotational thromboelastometry confirmed that MSC infusion did not induce a procoagulant profile (**Supplementary Figure 3**). Elevated basal MCF-fibTEM and MCF-exTEM values were likely due to increased fibrinogen. Four patients (P2, P3, P4, P6) had baseline thrombocytosis, consistent with reactive thrombocytosis. Platelet counts normalized in P4 and P6 after treatment in the ST and LT, respectively.

No deposition of anti-C7 antibodies was detected pre- or post-treatment (Day 56). Elevated circulating anti-C7 antibodies were observed in P1 and P2 at baseline and persisted in P1 post-infusion (**Supplementary Table 11**).

### 3.5 Efficacy analysis

Individual and global responses to haploidentical BM-MSC were evaluated in terms of mucocutaneous involvement, inflammation, pain, pruritus and health-related quality of life (HRQOL). Intraindividual changes were compared in the short term (ST; median of V3–V6; day 21 to 90) and long term (LT; V7–V9; day 180 to 360) post-treatment relative to basal status (B; V0–V2; 90 days pre-treatment). Global analyses were performed using the median-of-medians and paired comparisons across eight patients.

**Figure 2:**
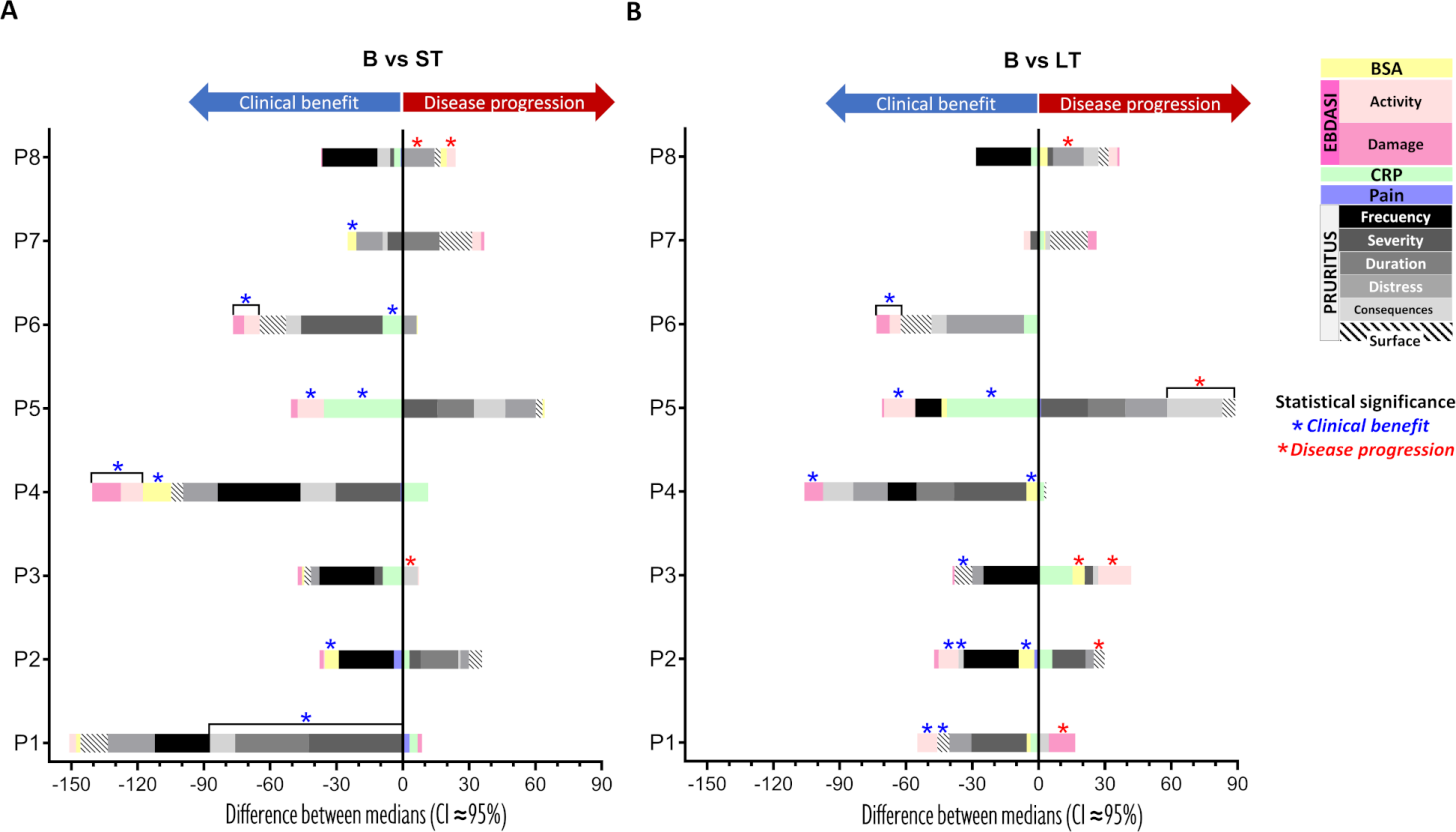
Clinical individual benefits in RDEB patients following haploidentical BM-MSC systemic therapy by assessing five efficacy outcomes: percentage of affected skin (BSA), levels of C-reactive protein (CRP) as a systemic inflammation marker, pruritus (Leuven Itch Scale, LIS 1.0), pain (Wong-Baker Faces scale) and disease severity (Epidermolysis Bullosa Disease Activity and Scarring Index: EBDASI total and, activity and damage subscores). Bars represent differences between medians before and after treatment, at **(A)** short term (ST) and **(B)** long term (LT). Negative values (left of the zero-line) indicate clinical improvement, while positive values (right) indicate disease progression. Statistical significance at a 95% confidence interval. BSA, CRP and EBDASI damage showed improvement or stabilization in seven out of eight patients, with statistical significance in four patients (P4, P5, P6 and P7). Additionally, different aspects of pruritus significantly improved in two patients (P1 and P6). Remarkable effects also noted in LT in four patients (P1, P2, P5 and P6). To account for inter-individual variability, patients with statistically significant improvements in two or more key parameters were classified as good responders, while those with significant shifts in single parameters or consistent biological trends were identified as mild responders.Basal **(B)** medians from V0 (-90D), V1 (-15D) and V2 (D0); Short Term (ST) medians from V3 (D21), V4 (D42), V5 (D56) and V6 (D90) and Long Term (LT) medians from V7 (D180), V8 (D270) and V9 (D360). D= days.

All eight patients demonstrated clinical benefit, with at least one efficacy parameter showing a significant ST and/or LT improvement in seven individuals (**Figure 2**, blue asterisk). According to the multidimensional assessment defined in Methods, five patients were classified as “good responders,” with significant EBDASI reductions along with additional improvements in itching (P1, P2), CRP (P5, P6) or BSA (P2, P4). However, no statistically significant global changes were observed in EBDASI (total, activity or damage scores), pain or acute-phase markers (**Supplementary Table 12**). Minor improvements in weight, height, BMI and other nutritional parameters were recorded (**Supplementary Table 13**).

**Figure 3:**
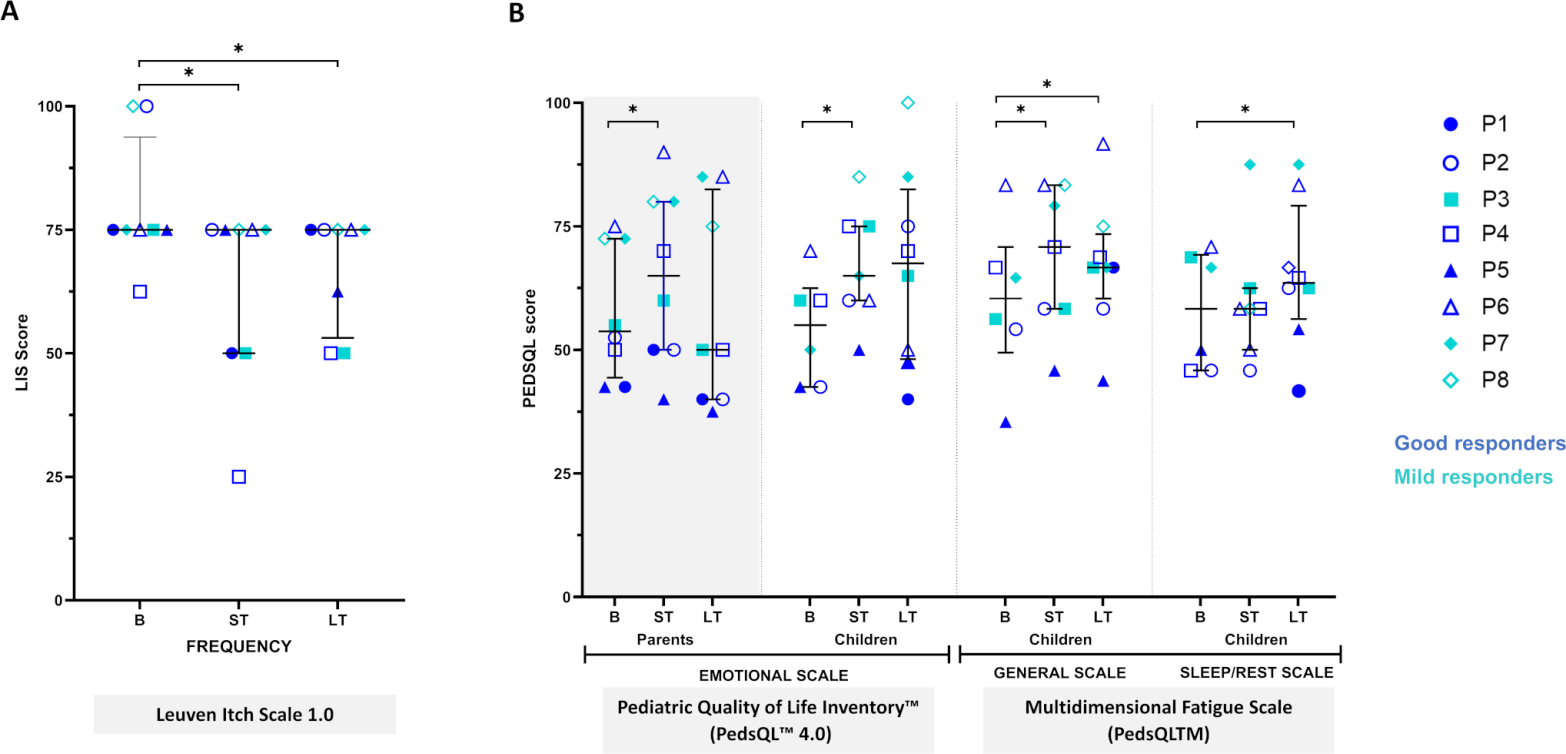
Global impact on itch and quality of life in the RDEB cohort following haploidentical BM-MSC systemic therapy. Symbols represent patient median scores while bars display pooled medians with interquartile ranges (IQR). Statistical significance (1-sided paired Wilcoxon test: *p ≤ 0.05, **p ≤ 0.01 and ***p ≤ 0.001. **(A)** Pruritus assessed by the Leuven Itch Scale (LIS 1.0), scoring 0-100 (100=most severe). Significant global differences observed in itch frequency, but not in the other dimensions (**Supplementary Table 14**); though individual variations were noted (**Supplementary Table 15**). **(B)** Health-related quality of life (HRQOL) assessed by the PedsQL™ 4.0, the Multidimensional Fatigue Scale and the Family Impact Module. Each module includes both child self-reports and parent proxies. Scores range from 0 to 100, with higher scores indicating better HRQOL. Significant global changes observed in emotional scales for both parents and children, as well as in general and sleep/rest fatigue scales for children. No global differences in other dimensions (**Supplementary Table 16**); though individual variations were noted (**Supplementary Tables 17**-**21**). Basal (B) medians from V0 (-90D), V1 (-15D) and V2 (D0); Short Term (ST) medians from V3 (D21), V4 (D42), V5 (D56) and V6 (D90) and Long Term (LT) medians from V7 (D180), V8 (D270) and V9 (D360). D= days.

In this cohort, there was a significant global reduction in the frequency of pruritus that persisted in the long term (p=0.03) (**Figure 3A**; **Supplementary Tables 12**, **14**). Also, fatigue and sleep/rest scores on the PedsQL™ Fatigue Module improved significantly in the long term (p=0.05) (**Figure 3B**, **Supplementary Table 15**). Both children and caregivers reported significant emotional well-being improvements on the PedsQL™ 4.0 Emotional Scale in the ST (p=0.05 and p=0.02, respectively) (**Figure 3B****;** **Supplementary Table 15**). Individual analyses of health-related HRQOL domains are detailed in **Supplementary Table 15**–**20**.

### 3.6 Mucocutaneous response

**Figure 4:**
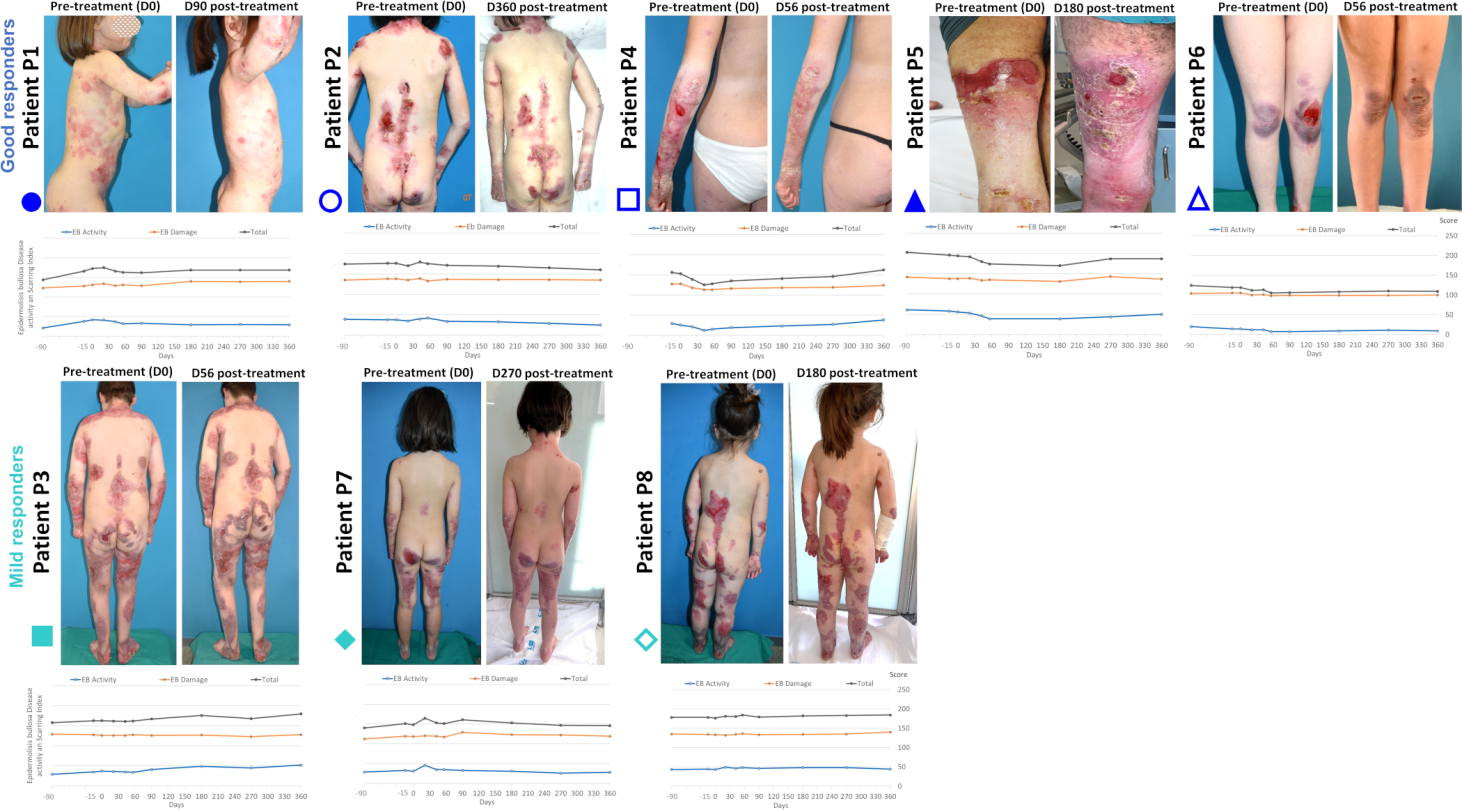
Mucocutaneous response in RDEB patients following haploidentical BM-MSC systemic therapy. Representative photographs before and after treatment showed a reduction in lesions and affected area and improved wound healing in good responders (top row) compared to mild responders (bottom row). Graphs represent individual evolution of EBDASI total scores and sub-scores (activity and damage) three months and one year before and after treatment, respectively. The total EBDASI score decreased one-year post-treatment in three good responders, with no increased damage in any of them. All patients provided consent for the publication of their photographs.

**Figure 5:**
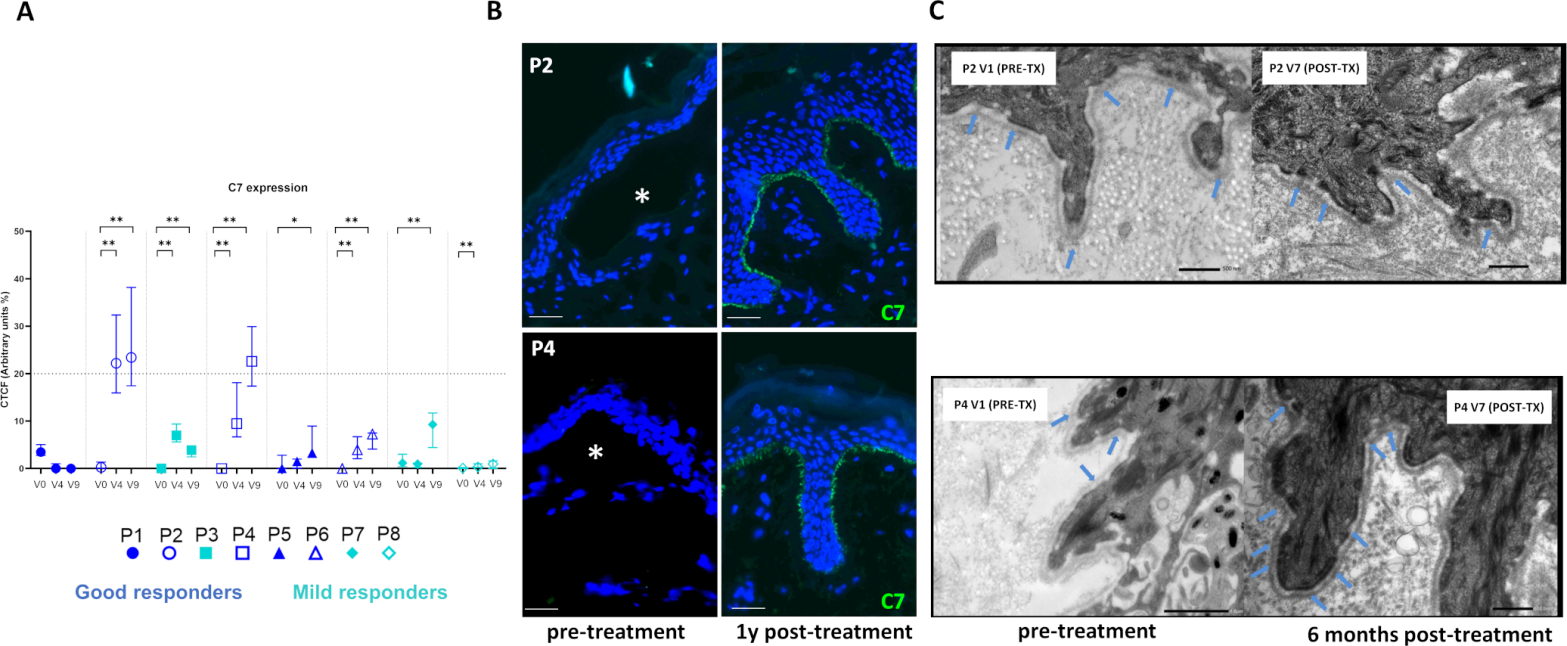
Type VII collagen (C7) expression in RDEB patients following haploidentical BM-MSC systemic therapy. **(A)** C7 detected by indirect immunofluorescence using a monoclonal LH7.2 antibody (NC1 domain) on skin sections collected at different time points. Relative C7 expression (percentage of control) calculated from corrected total fluorescence (CTCF), quantified in five random fields per sample in ImageJ. Graphs show individual median values (IQR). Statistical significance (1-sided unpaired Mann-Whitney U test): *p ≤ 0.05, **p ≤ 0.01, ***p ≤ 0.001. Notably, C7 expression in P2 and P4 increased significantly, reaching slightly over 20% of control levels. **(B)** C7 staining in P2 and P4 increased one year post-treatment *vs.* minimal baseline expression. White asterisks denote dermal-epidermal separation. Scale bars: 50 μm. **(C)** TEM showed no mature AF at baseline or six months in both patients; thin rudimentary AFs (blue arrows) slightly increased after treatment. Scale bars: 0.25 μm. AF, anchoring fibrils; TEM, Transmission electron microscopy. Basal (B) medians from V0 (-90D), V1 (-15D) and V2 (D0); Short Term (ST) medians from V3 (D21), V4 (D42), V5 (D56) and V6 (D90) and Long Term (LT) medians from V7 (D180), V8 (D270) and V9 (D360). D, days; V, visit.

In good responders, wound areas and skin erosions qualitatively decreased (**Figure 4**), although no donor cells were detected in skin biopsies at 180 days post-injection. Three patients showed notable EBDASI activity reductions. Global and individual analyses revealed increased C7 levels post-treatment in multiple patients, exceeding 20% of normal levels in P2 and P4 (**Figure 5**; **Supplementary Figure 4**). Though rudimentary anchoring fibrils were observed, mature fibrils were absent (**Figure 5C**).

Clinically meaningful reductions in EBDASI activity (>9 points) were observed in P4 (ST) and P5 (ST and LT). P1 and P2 showed a 9-point reduction and P6 a 5-point reduction in LT. Conversely, P3 and P8 showed disease progression (EBDASI activity increase >3 points) (51). EBDASI damage and percentage of BSA remained stable, except for P1 and P3, respectively.

### 3.7 Systemic inflammation response

**Figure 6:**
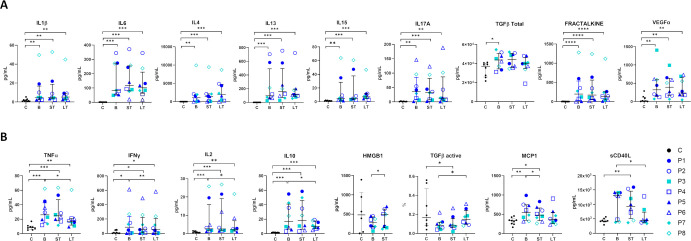
Circulating cytokine levels in RDEB patients following haploidentical BM-MSC systemic therapy. **(A, B)**. Individual median data (symbols) and pooled medians with interquartile ranges (bars) at day 0 in RDEB patients (P, n=8) compared with control medians (C, n=8 unaffected individuals within the same age range). Outliers (1.5×IQR) were excluded. Global cohort comparisons assessed by the one-tailed unpaired Mann–Whitney U test and intraindividual changes from baseline by the one-tailed paired Wilcoxon test. Statistical significance: *p ≤ 0.05, **p ≤ 0.01 and ***p ≤ 0.001. A. Elevated cytokines at basal status did not significantly change post-treatment. **(B)** Cytokines with significant changes post-treatment. Basal status (B) medians from V0 (-90D), V1 (-15D) and V2 (D0); Short Term (ST) medians from V3 (D21), V4 (D42), V5 (D56) and V6 (D90) and Long Term (LT) medians from V7 (D180), V8 (D270) and V9 (D360). D, days; V, visit.

**Figure 7:**
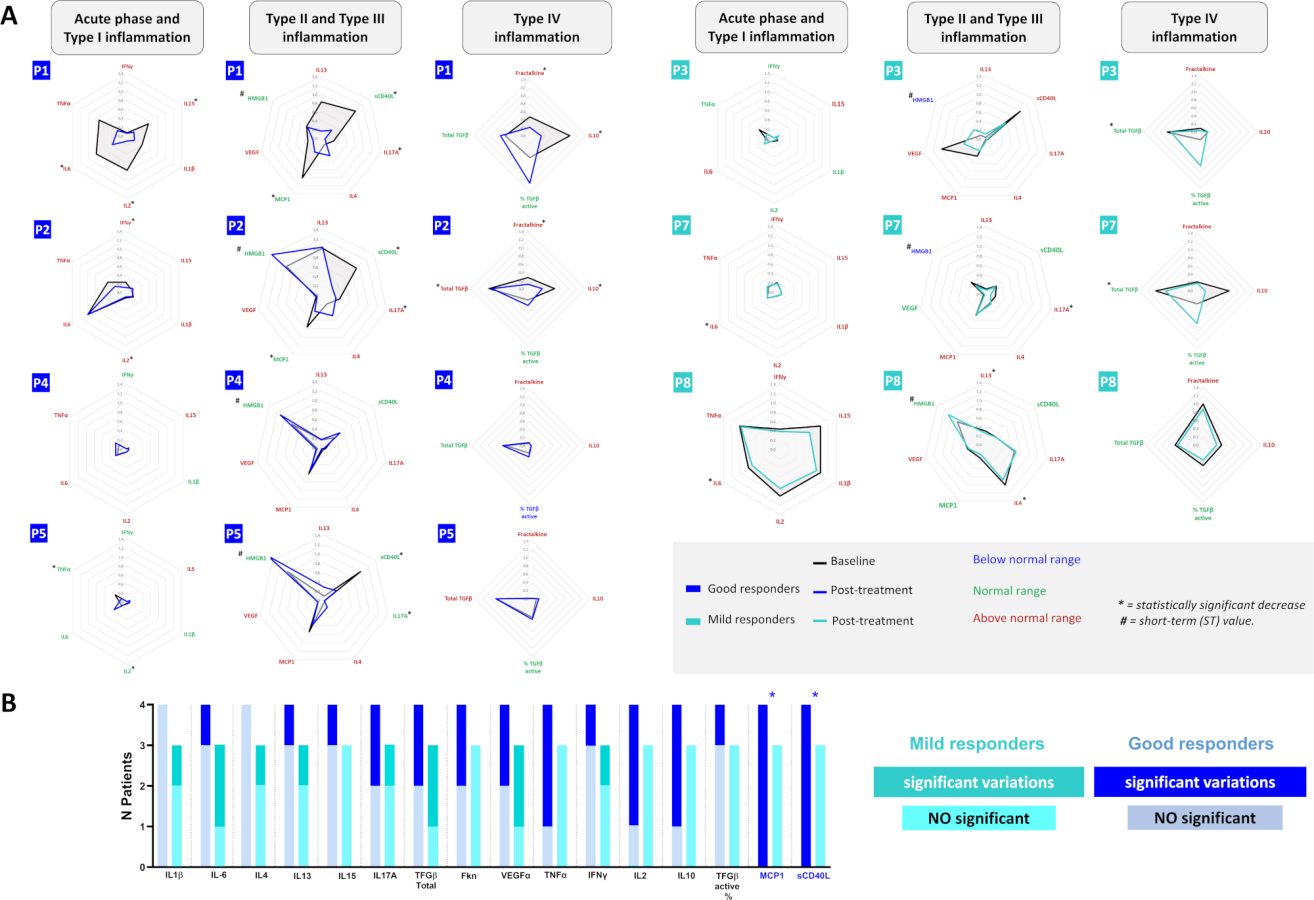
Individual cytokine profiles across inflammatory patterns after haploidentical BM-MSC Therapy in RDEB. Spider plots summarizing the effect of treatment, with cytokines classified according to the proposed model of cutaneous inflammation in DEB across disease stages (11, 12), while including a broader panel of cytokines. Each plot forms a polygon connecting values normalized to the control. Basal and post-treatment cytokine patterns within each inflammatory type are unique for each patient. Differences between good responders and mild responders are highlighted in panel **(B)** Fisher’s exact test analysis, based on significant changes in two or more clinical parameters (good responders), revealed a statistically significant correlation between decreases in MCP1 and sCD40L and treatment response (*p = 0.03, two-sided). sCD40L normalized in all good responders except P4, who had normal basal levels. MCP1 normalized in two good responders (P1 and P2) and was already normal at baseline in P6. No other cytokine showed a correlation with response.

An exploratory analysis of 18 circulating cytokines and immunomodulators were quantified. Most of them were elevated in RDEB patients at baseline, except HMGB1, TGFβ (active) and sCD40L (**Figures 6A,B**). Post-treatment, statistically significant LT reductions in TNFα, IFNγ, IL2 and IL10 were noted, though levels remained above control values. Global MCP1 and sCD40L levels normalized in the LT (**Figure 6B**). Cytokine profiles across inflammatory patterns (Type I-Type IV) revealed that both basal and post-treatment patterns were unique for each patient (**Figure 7A**). Despite this variability, the Fisher’s exact test revealed a statistically significant association between good responders and relevant variations in MCP1 and sCD40L levels (p=0.03), indicating a potential role as efficacy biomarkers (**Figure 7B**). No consistent changes were found in other cytokines.

**Figure 8:**
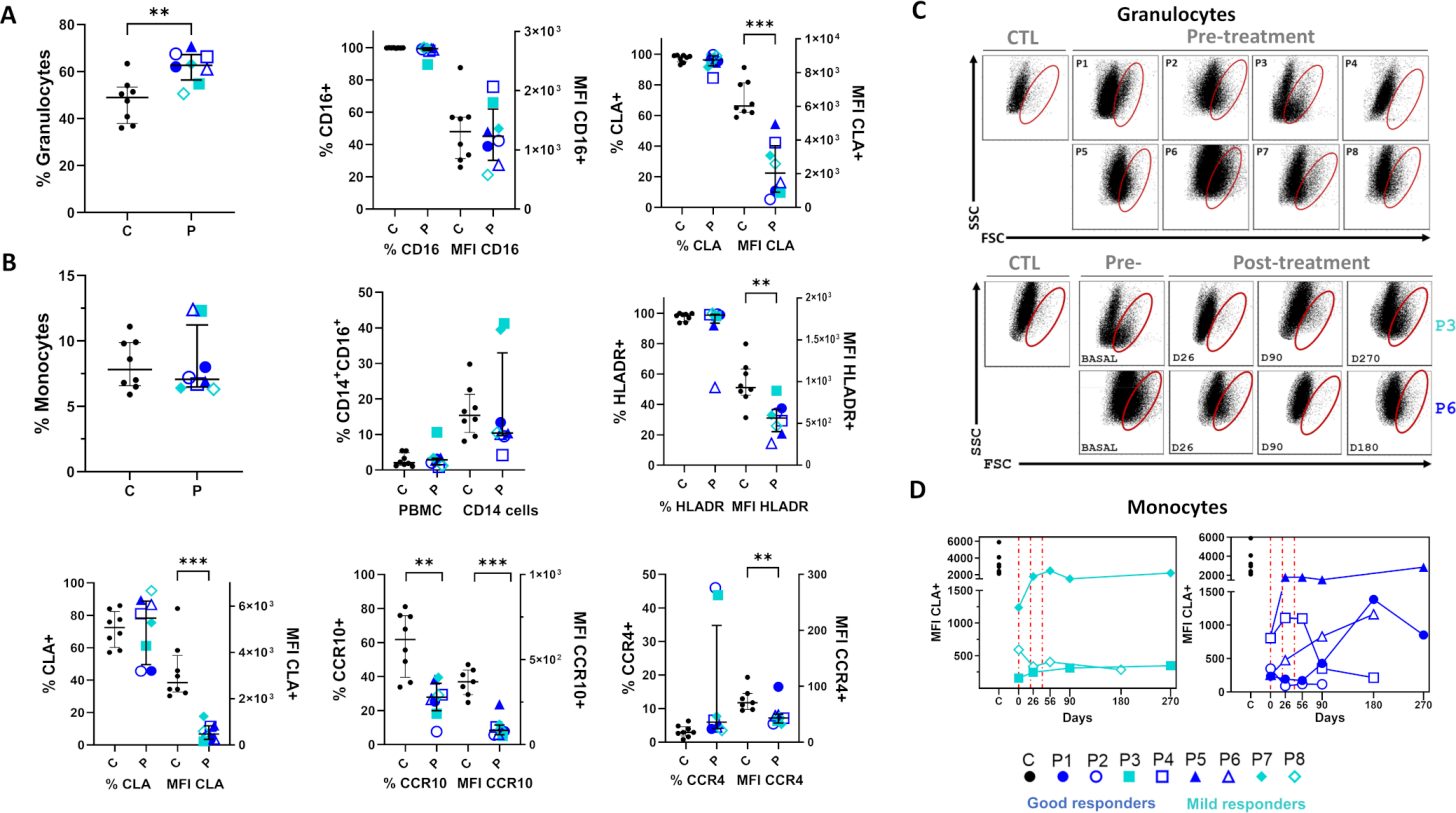
Relevant changes in myeloid populations in RDEB patients following haploidentical BM-MSC systemic therapy, assessed by flow cytometry. **(A, B)** Individual data (symbols) and medians with interquartile ranges (bars) at day 0 in RDEB patients (P, n=8) and control medians (C, n=8 unaffected individuals within the same age range). Outliers (1.5×IQR) were excluded. Statistical significance (one-tailed unpaired Mann–Whitney U test): *p ≤ 0.05, **p ≤ 0.01. A. Percentages of circulating granulocytes and CD16^+^ and CLA^+^ subsets were not significantly different in RDEB, but mean fluorescence intensity (MFI) of CLA^+^ cells decreased. **(B)** Percentages of circulating classical monocytes and subsets with mean fluorescence intensity (MFI) for HLA-DR^+^, CLA^+^, CCR4^+^ and CCR10^+^ cells. No significant global differences observed in CD14^+^CD16^+^ monocytes at baseline, despite increases in P3 and P7 (mild responders) and decreases in the remaining patients. **(C)** Dot plots of granulocytes at day 0 (baseline) in RDEB patients and selected post-treatment examples from each response group. Representative control samples are also shown. Anomalous population observed in all individuals except the control and P4 (red oval). **(D)** Monocyte CLA^+^ MFI showed improvement in P4, P5 and P6 (good responders) and P7 (mild responder) post-treatment. Vertical grids indicate treatment days.

**Figure 9:**
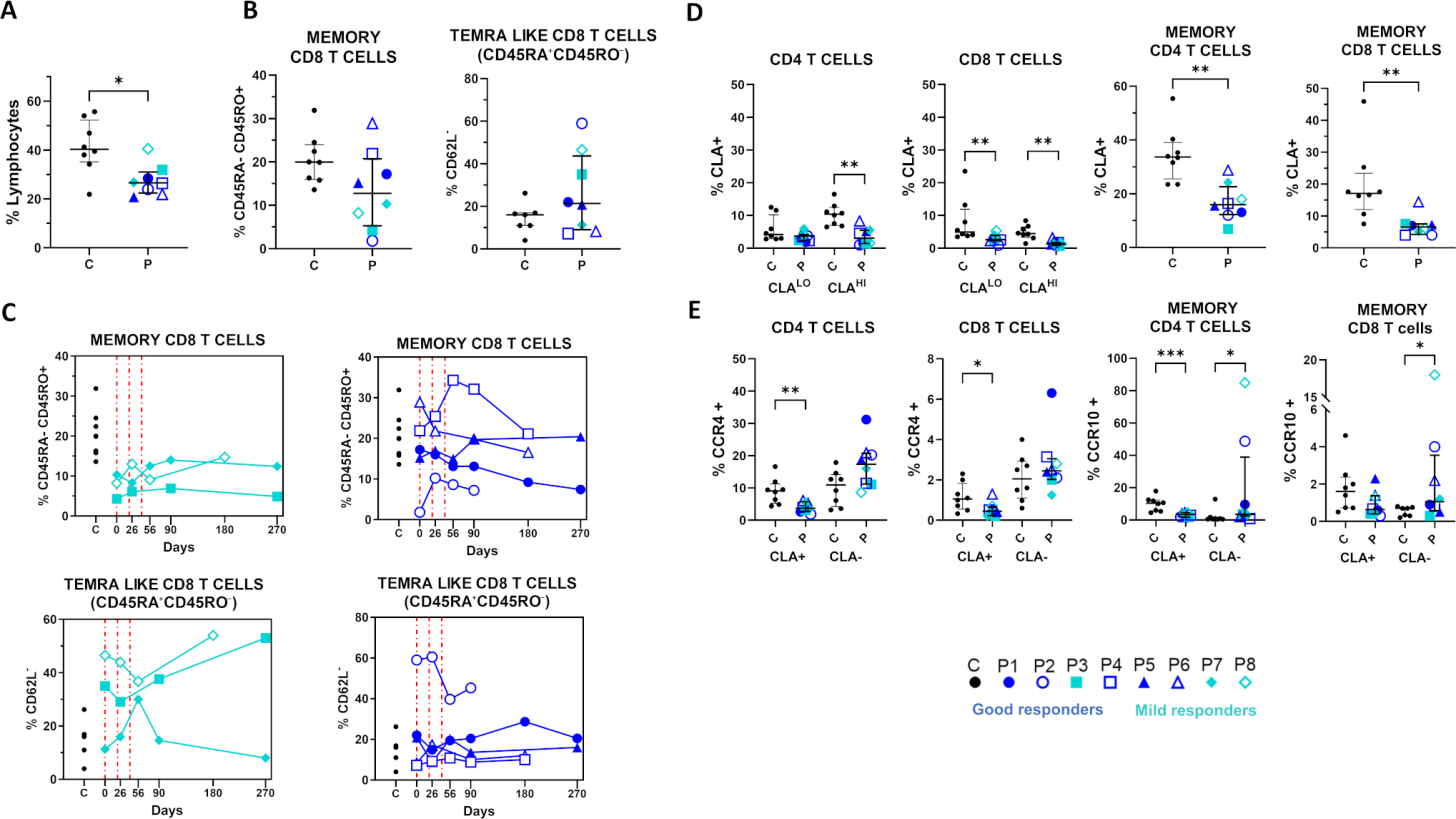
Relevant changes in lymphoid populations in RDEB patients following haploidentical BM-MSC systemic therapy, assessed by flow cytometry. **(A, B, D, E)**. Individual data (symbols) and medians with interquartile ranges (bars) at day 0 in RDEB patients (P, n=8) and controls (C), n=8 unaffected individuals within the same age range). Outliers (1.5×IQR) were excluded. Statistical significance (one-tailed unpaired Mann–Whitney U test): *p ≤ 0.05, **p ≤ 0.01. A. Percentages of total T lymphocytes (CD3^+^) significantly decreased in the RDEB cohort. **(B)** Percentages of memory (CD45RA^+^CD45RO^+^) and TEMRA-like (CD26L, CD45RA^+^CD45RO^-^) CD8^+^ T cells not significantly different in RDEB cohort. However, mild responders and P2 showed reduced memory CD8^+^ T cells with increased CD62L^-^ TEMRA-like subsets. **(C)** Post-treatment, memory CD8^+^ T cells transiently improved in P2 (good responder) and normalized in P7 and P8, with a decrease in CD62L^-^ TEMRA-like subsets. No relevant changes observed in these subsets in the remaining patients. Vertical grids indicate treatment days. **(D)** Percentages of skin-homing CD4^+^ and CD8^+^ T cells (CLA^+^) reduced in RDEB patients, especially within the CD45RA^-^CD45RO^+^ memory subset. **(E)** Percentage of CCR4^+^ and CCR10^+^cells increased in memory CD4^+^ and CD8^+^ T cells lacking CLA.

The impact of the treatment on PBL populations by flow cytometric analysis included immunophenotyping of both general leukocyte populations and specific subsets associated with mucocutaneous tissue recruitment or linked to inflammatory and infectious stress (52, 53). RDEB patients showed increased granulocyte percentages (**Figure 8A**), comparable monocyte percentages (**Figure 8B**) and reduced lymphocyte percentages (**Figure 9A**), relative to controls.

Within the myeloid population, a distinct granulocyte subset of larger size (suggestive of immaturity) was present in 87.5% of patients at basal status and transiently decreased or disappeared in 62.5% of them after treatment, in both good and mild responders (**Figure 8C**; **Supplementary Figure 5B**). This transient decrease was observed after the second infusion (D26), persisting for at least 90 days (and up to 270 days in some patients). Globally, frequencies and subset distributions of non-classical and intermediate monocytes (CD16+) were within the range observed in controls (**Figure 8B**). However, RDEB monocytes had significantly lower expression of HLADR/DQ and skin-homing markers (CLA, CCR10, CCR4). Treatment did not alter HLADR/DQ MFI (**Supplementary Figure 5C**) but CLA expression increased in 50% of the cohort reaching normalization in a good (P5) and a mild responder (P7) (**Figure 8D**).

Regarding lymphoid populations, the total percentages of B, T, NK and NKT cells were not significantly different from controls (**Supplementary Figure 6**) and post-treatment changes were not relevant (**Supplementary Figure 7**). This included CD4^+^ T populations with regulatory phenotypes (CD25^+^, CD25^+^CD127^-^, CD25^+^CD39^+^, or CD25^+^CD73^+^), which showed no relevant differences between RDEB patients and controls (data not shown). In the cohort, 50% (mostly mild responders) had markedly reduced memory CD8^+^ T cells (CD45RA^-^CD45RO^+^). Of these four patients, three had an increased proportion of CD62L^-^ CD8^+^ T cells within the CD45RA^+^CD45RO^-^ subset, suggestive of TEMRA-like differentiation (**Figure 9B**). After treatment, memory CD8^+^ T cells increased in 3/4 patients reaching normalization in two of them, along with a transient reduction of TEMRA-like cells (**Figure 9C**). Percentages of skin-homing CD4^+^ and CD8^+^ T cells (CLA^+^) were reduced in RDEB patients, particularly within the CD45RA^-^CD45RO^+^ memory subset (**Figure 9D****).** While CCR4 and CCR10 expression was suppressed in CLA^+^ T cells, the expression of both receptors was increased in memory T CLA^-^ (**Figure 9E**). None of these T cell subpopulations normalized following treatment (data not shown). Notably, NKT cells were not significantly altered, except in P3 (mild responder) and P5 (good responder), who had high levels that normalized post-treatment in P5 (**Supplementary Figures 6C**, **7C**).

## 4 Discussion

MesenSistem-EB is the first clinical trial to evaluate systemic haploidentical BM-MSC monotherapy in RDEB, comprehensively assessing its effects on systemic inflammation, including peripheral blood leukocytes. It demonstrated that three intravenous infusions of 2–3 × 10^6^ cells/kg at 21-day intervals were well tolerated, providing variable clinical benefits. The mechanism of action appears to involve MCP1 and sCD40L dynamics, which emerge as potential biomarkers of efficacy, affecting circulating myeloid and lymphoid compartments and supporting anti-inflammatory effects rather than durable engraftment or sustained C7 deposition.

Allogeneic MSC are attractive due to their low immunogenicity—characterized by minimal MHC class I expression and absence of MHC class II and costimulatory molecules—enabling transplantation without extensive pre-conditioning. This has been consistently supported in clinical trials (37, 54), where minimal premedication (e.g., antihistamines or corticosteroids) sufficed. However, despite their low immunogenicity, MSC are not immune to host rejection, often via allo-recognition by NK, B and T cells, which compromises their persistence and efficacy (55–57). Notably, MSC persist longer in immunodeficient mice than in immunocompetent models (58). Haploidentical donors may confer greater immunotolerance and enhance MSC persistence and function, by reducing the repertoire of non-self HLA antigens presented to the host immune system. This strategy, underexplored in RDEB, where unrelated donor BM-MSC are most commonly used (33) (**Supplementary Table 21****),** seeks to mitigate the well-documented cellular and humoral alloimmune responses that can lead to the premature clearance of allogeneic MSCs in immunocompetent recipients (55, 58). By potentially extending the survival of infused cells, this approach may broaden the therapeutic window for paracrine signaling. This rationale led to the use of haploidentical BM-MSC in the MesenSistem-EB study, with individual batches manufactured for each patient from one of their parents (sex-mismatch), to facilitate tracking of donor cells in the patient skin. All BM-MSC batches suppressed T cell proliferation (median inhibition ~80%), exhibited anti-inflammatory secretory profiles and retained trilineage differentiation potential. However, two batches (HP3, HP8) had reduced adipogenic/osteogenic differentiation and were associated with mild responders. Additionally, IL8 and PGE2, key mediators in MSC immunomodulation (59–61) varied across batches and may have influenced outcomes. Indeed, using a single BM-MSC batch would reduce inter-variability. From an economic perspective, producing haploidentical cells from a single donor source would have reduced costs by approximately 20%. Specifically, the total cost for eight patients would have decreased from €148,000 (€18,500 per patient) to €116,000 (€14,500 per patient).

Consistent with previous CT, rejection of allogeneic MSC has not raised significant safety concerns in MesenSistem-EB, with no SAE and mild and transient AE (n=127) such as chills and hypotension being the most common (33, 37, 38, 62). Only 3.9% of AE related to MSC infusion—all mild and resolving within 24 hours. Fever (87.5%) and low-grade fever (62.5%) were the most frequent AE. Headache occurred in 14.8% of patients, though no direct association with MSC was confirmed in prior meta-analyses (37).

Despite isolated reports of MSC-related thrombotic events (63), no coagulation abnormalities or thrombotic episodes were observed in MesenSistem-EB, consistent with the broader safety data (37). Renal and hepatic parameters remained stable, except for one case of elevated GGT. Although some patients had circulating anti-C7 antibodies, no dermal deposition was observed, even in cases with clinically increased C7 expression, reinforcing findings that autoantibodies in RDEB are common but often clinically silent (64).

While MSC are not considered tumorigenic (37), the high baseline risk of SCC in RDEB warrants vigilant monitoring. In one prior RDEB trial, two adults developed SCC 6–7 months after MSC infusion (32), though this likely reflects disease progression, as SCC incidence increases with age. No malignancies occurred during the MesenSistem-EB trial.

In terms of efficacy, 62.5% of patients (5/8) were classified as good responders, with improvements in at least two key clinical parameters (e.g., EBDASI, BSA, CRP, pruritus). Notably, 80% of good responders (4/5) exhibited a >9-point reduction in EBDASI activity (51)—an outcome consistent with prior MSC trials (**Supplementary Table 21**) (30–32, 62, 65–67). While individual responses were evident, global analyses reached statistical significance only for pruritus, sleep and fatigue, reporting the second-longest duration of clinical benefit (5). Although the lack of broader significance could be attributed to the small cohort size combined with the use of different MSC batches, results from the MissionEB trial (ISRCTN14409785), —with 36 patients, a single batch and a placebo-controlled design—show a comparable pattern, with no significant improvement in the primary EBDASI outcome at three months. Nonetheless, MissionEB provides valuable subgroup and long-term data (62) while underscoring the intrinsic challenges of demonstrating efficacy in heterogeneous RDEB populations. MSC have not yet been tested in other EB subtypes. This is particularly relevant given the natural history of systemic inflammation in severe RDEB. Longitudinal data (14) identify a critical inflection point where CRP levels, already elevated in early childhood (~20–30 mg/L), typically rise up to 18-fold during adolescence. Thus, a measurable increase would be expected within the one-year timeframe of our study for a cohort in the 4–17 age range. However, despite a severe phenotype of the cohort, the global median CRP remained stable from baseline (20.9 mg/L) to the 1-year follow-up (20.4 mg/L). This stabilization during a phase of anticipated inflammatory escalation suggests a potential clinical benefit by effectively “flattening the trajectory” of systemic inflammation. Indeed, larger cohorts with control arms and longer follow-up periods are needed to confirm the durability of this effect.

MSC delivery route critically influences therapeutic action. Intravenous infusion enables systemic distribution but may limit homing to skin, with MSC often sequestered in lungs (68). This supports a “hit-and-run” mechanism, where MSC transiently modulate local inflammation via paracrine signaling (33, 69, 70). No donor-derived cells were detected in skin. However, two patients (P2, P4) exhibited >20% C7 expression post-treatment, indicating functional benefit without engraftment. Cell tracking studies are needed to clarify MSC fate. Moreover, given the generally limited C7 deposition at the dermal–epidermal junction and variability of clinical benefits after MSC therapy (30–32, 62, 65–67), dose standardization is essential. In RDEB studies, MSC of different sources doses have ranged from 1 to 3.5 × 10^6^ cells/kg, typically administered in one to three intravenous infusions at one to three-week intervals, complicating comparisons across studies (**Supplementary Table 21**). Future approaches may include MSC priming, alternative delivery methods or the use of extracellular vesicles.

RDEB’s inflammatory landscape fluctuates and influences MSC function. Globally, patients exhibited elevated levels of multiple cytokines at baseline, including Type I-associated (IFNγ, IL1β, IL2, IL6, IL15), Type II and III (IL4, IL13, IL17A, MCP1) and Type IV inflammation (IL10), indicating a broadly activated, mixed immune profile. While IL10 and MCP1 could be considered transversal modulators of inflammation, they are assigned to Type IV and III, respectively, to maintain consistency with the classification framework used in this analysis. This complex profile may be reflecting overlapping chronic and acute inflammation, accompanied by ongoing tissue damage (11, 12). Post-treatment decreases in TNFα, IFNγ, IL2 and IL10, together with increased active TGFβ and HMGB1 levels, may indicate a shift toward immune recovery with reduced systemic inflammation and enhanced regulatory and reparative responses. Normalization of MCP1 and sCD40L—especially in good responders—correlated with reduced CRP and improved clinical parameters. MCP1, implicated in fibrosis and cancer progression (71), may serve as a predictive biomarker, as might sCD40L, which plays a role in autoimmunity and alloimmunization (72). Further validation in larger, independent cohorts is required to confirm these exploratory findings, establish causality and definitive cut-off values.

MSC exert immunoregulatory effects by altering dendritic cells, NK cells and T/B lymphocytes (70) and have been described to promote Treg induction (73). In MesenSistem-EB, at baseline granulocyte and monocyte alterations consistent with systemic inflammation were observed in agreement with recent findings (6). Notably, immature granulocyte populations decreased in good and mild responders, post-treatment. This common biological shift highlights a consistent anti-inflammatory effect, justifying the inclusion of mild responders as a relevant clinical category beyond EBDASI scores and distinguishing them from non-responders. Monocytes showed improved CLA expression after treatment, which is a known skin-homing marker for T cells, suggesting a potential enhancement of their skin-homing capacity, although this has not been formally studied in monocytes (52, 74–76). Given the roles of MCP1 and sCD40L in monocyte recruitment and activation, their normalization may align with these post-treatment changes. Lymphocyte subsets showed more modest changes, though memory CD8^+^ T cells increased post-treatment mostly in mild responders alongside a reduction in CD62L^-^ CD8^+^ T cells within the CD45RA^+^CD45RO^-^ subset, consistent with TEMRA-like differentiation (77). This shift may reflect a skewing toward terminal effector states at the expense of memory maintenance, possibly driven by sustained activation or inflammatory pressure. However, persistently low CLA+ T cells underscore ongoing immune dysfunction, particularly in skin (75, 78). Interestingly, in other inflammatory skin diseases such as atopic dermatitis and psoriasis, CLA antigen has been proposed as a therapeutic target and the frequency of circulating CLA^+^ T cells as a biomarker for disease activity and treatment response, highlighting the clinical relevance of this axis (79). Whether persistent CLA downregulation in RDEB reflects irreversible mucocutaneous damage or long-term immune adaptation remains to be elucidated.

This academic clinical trial is inherently limited by its uncontrolled, single-arm design and the small sample size of only eight patients, all representing the most severe RDEB subtype. Nevertheless, given the rarity of the condition, assembling larger cohorts remains highly challenging. Importantly, the outcomes of MesenSistem-EB not only extend and align with previous MSC studies, including the only recent placebo-controlled trial, but also provide novel insights into patient-specific inflammatory profiles, peripheral blood immune compartments and cytokine dynamics. These data underscore the importance of determining baseline inflammatory status, as systemic inflammation is an unmet need that may influence the efficacy of MSCs and other therapies, including gene-based interventions. Our multidimensional categorization of good and mild responders seems to be a rational and useful approach to capture relevant biological shifts that global statistics often mask, and should be further tested in larger, ideally multicenter studies. Haploidentical BM-MSCs appear to mediate therapeutic effects primarily through paracrine and anti-inflammatory mechanisms rather than durable engraftment or sustained C7 deposition. Key cytokines, particularly MCP1 and sCD40L, emerged as candidate biomarkers of treatment efficacy, highlighting potential tools for monitoring and predicting responses that merit further validation. The observed heterogeneity in the response reflects both the intrinsic complexity of RDEB’s immune landscape and potential MSC batch variability. These findings support the use of MSC also as complementary therapy. Future randomized controlled trials integrating multi-omics and cell tracking will be essential to clarify mechanisms, personalize therapy and improve outcomes in RDEB.

## Data Availability

Data from this study are available to qualified academic researchers upon reasonable request to the corresponding author, in line with the participants’ consent. All shared data will be de-identified and comply with applicable privacy and legal regulations. Requests to access the datasets should be directed to mescamez@ing.uc3m.es.
