# Supplementary material for: MesenSistem-EB: systemic haploidentical mesenchymal stem cell therapy in recessive dystrophic epidermolysis bullosa associated with clinical benefits and correlated with MCP1 and sCD40L dynamics
Source: Front Immunol. 2026 May 5;17:1789537. doi: 10.3389/fimmu.2026.1789537 (PMC13183538; doi:10.3389/fimmu.2026.1789537)
Supplement: Supplementary file 1 [file DataSheet1.pdf]

frontiers

Supplementary Material

1. Supplementary Figures Page

Figure 1. *In vitro* differentiation and immunosuppressive capacity of haploidentical BM-MSC batches 2

Figure 2. Secretory profile of haploidentical BM-MSC in coculture 3

Figure 3. Coagulation study in RDEB patients following haploidentical BM-MSC systemic therapy 4

Figure 4. C7 expression in RDEB patients following haploidentical BM-MSC systemic therapy. 5

Figure 5. Myeloid cell subpopulation dynamics in RDEB patients following haploidentical BM-MSC therapy, assessed by flow cytometry 7

Figure 6. Lymphoid cell populations at baseline in RDEB patients, assessed by flow cytometry 8

Figure 7. Lymphoid cell population dynamics in RDEB patients following haploidentical BM-MSC therapy, assessed by flow cytometry 9

2. Supplementary Tables

Table 1. Schedule of activities in MesenSistem-EB 10

Table 2. Recruitment criteria for haploidentical bone marrow donors 11

Table 3. Demographics of unaffected individuals 11

Table 4. Specifications, formulation and quality controls of the BM-MSC investigational medicinal product 12

Table 5. Batch-Specific Properties of the investigational medicinal product from haploidentical BM-MSC donors 13

Table 6. BM-MSC final product release data 14

Table 7. Basal clinical features of the nine RDEB patient recruited in Mesensistem-EB 15

Table 8. Summary of emergent adverse events (AE) following haploidentical BM-MSC systemic therapy 16

Table 9. AEs by symptom type and relationship to MSC infusion 17

Table 10. Vital signs 18

Table 11. Autoantibodies following haploidentical BM-MSC systemic therapy 18

Table 12. Global analysis of the clinical benefits following haploidentical BM-MSC systemic therapy 19

Table 13. Global analysis of changes in the nutritional status following haploidentical BM-MSC systemic therapy 20

Table 14. Individual analysis of changes in pruritus by LIS 1.0 followi following haploidentical BM-MSC systemic therapy 21

Table 15. Global analysis of changes in HRQOL by the PedsQL measurement model following haploidentical BM-MSC systemic therapy 22

Table 16. Individual analysis of PedsQL™ 4.0 (parent forms) 23

Table 17. Individual analysis of PedsQL™ 4.0 (child forms) 24

Table 18. Individual analysis of PedsQL™ Multidimensional Fatigue Scale (parent forms) 25

Table 19. Individual analysis of PedsQL™ Multidimensional Fatigue Scale (child forms) 26

Table 20. Individual analysis of PedsQL™ 2.0 Family Impact Module (parent forms) 27

Table 21. Overview of clinical trials using mesenchymal stromal cells and subpopulations for the treatment of RDEB 29

1. Supplementary Figures

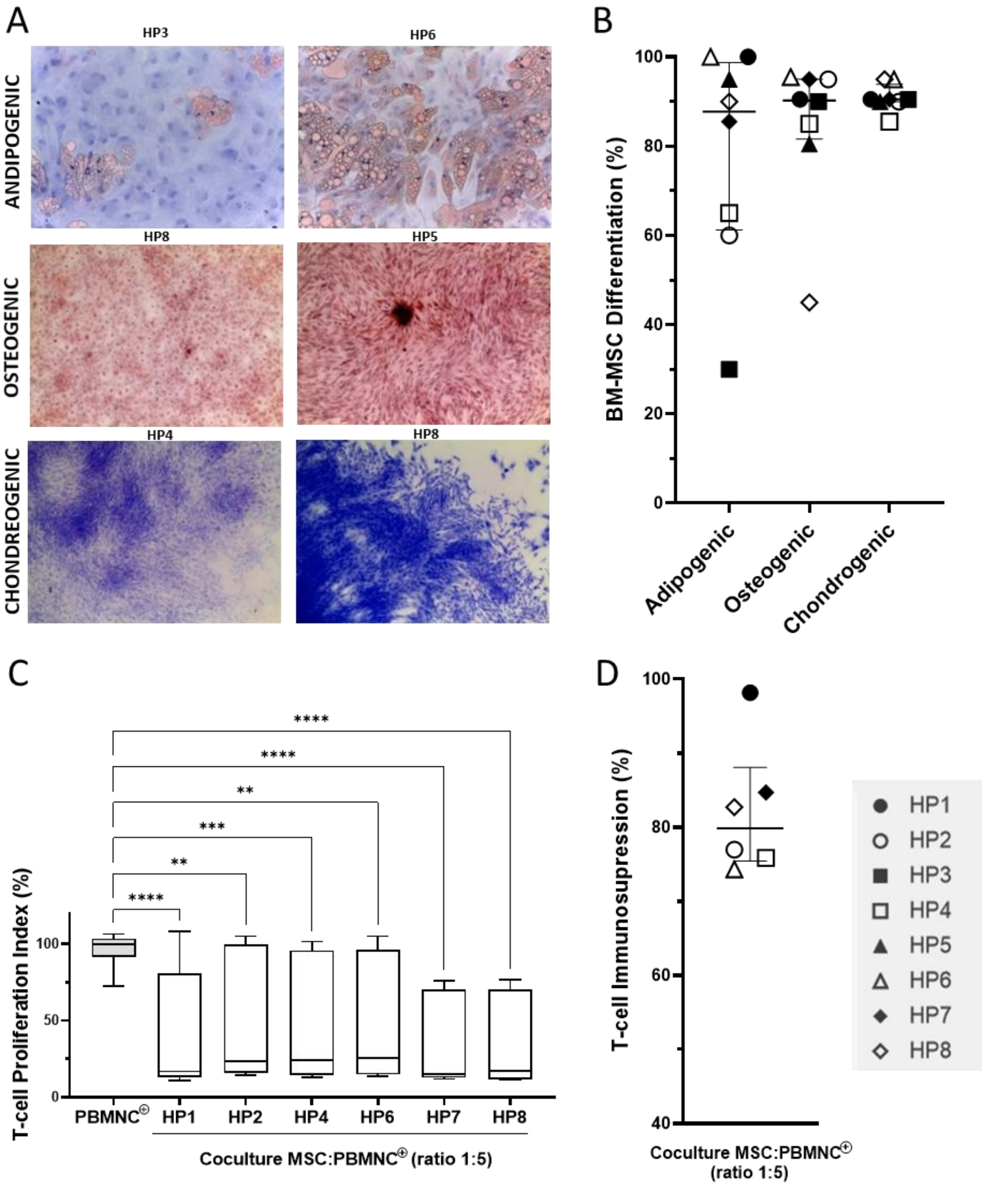

Supplementary Figure 1. *In vitro* differentiation and immunosuppressive capacity of haploidentical BM-MSC batches. A. Representative microphotographs of *in vitro* trilineage differentiation of BM-MSC by specific staining. Oil Red-Hematoxylin, Alizarin Red-Hematoxylin and Toluidine Blue showing vacuole lipids, calcium deposits and proteoglycans typical from adipogenic, ostogenic and chondrogenic differentiation, respectively. Images were adquired at 200x magnification. **B.** Global percentage of differentiation in vitro on the different lineages. **C.** In vitro immunosuppressive assay. Graph represents the percentage of proliferative index of human T cells from 3 different buffy coat donors, activated with anti-CD3/IL-2 and cocultured with the different BM-MSC batches. Statistical differences were calculated using the Kruskal-Wallis test followed by uncorrected Dunn's multiple comparisons test (*$p \leq 0.05$, **$p \leq 0.01$, ***$p \leq 0.001$, ****$p \leq 0.0001$). **D.** Percentage of T-cell proliferation suppression was calculated as 100% - [T-cell proliferation after coculture (% of positive control)] for every batch. In B, C and D graphs display median values and IQR, with symbols representing the percentage for each batch.

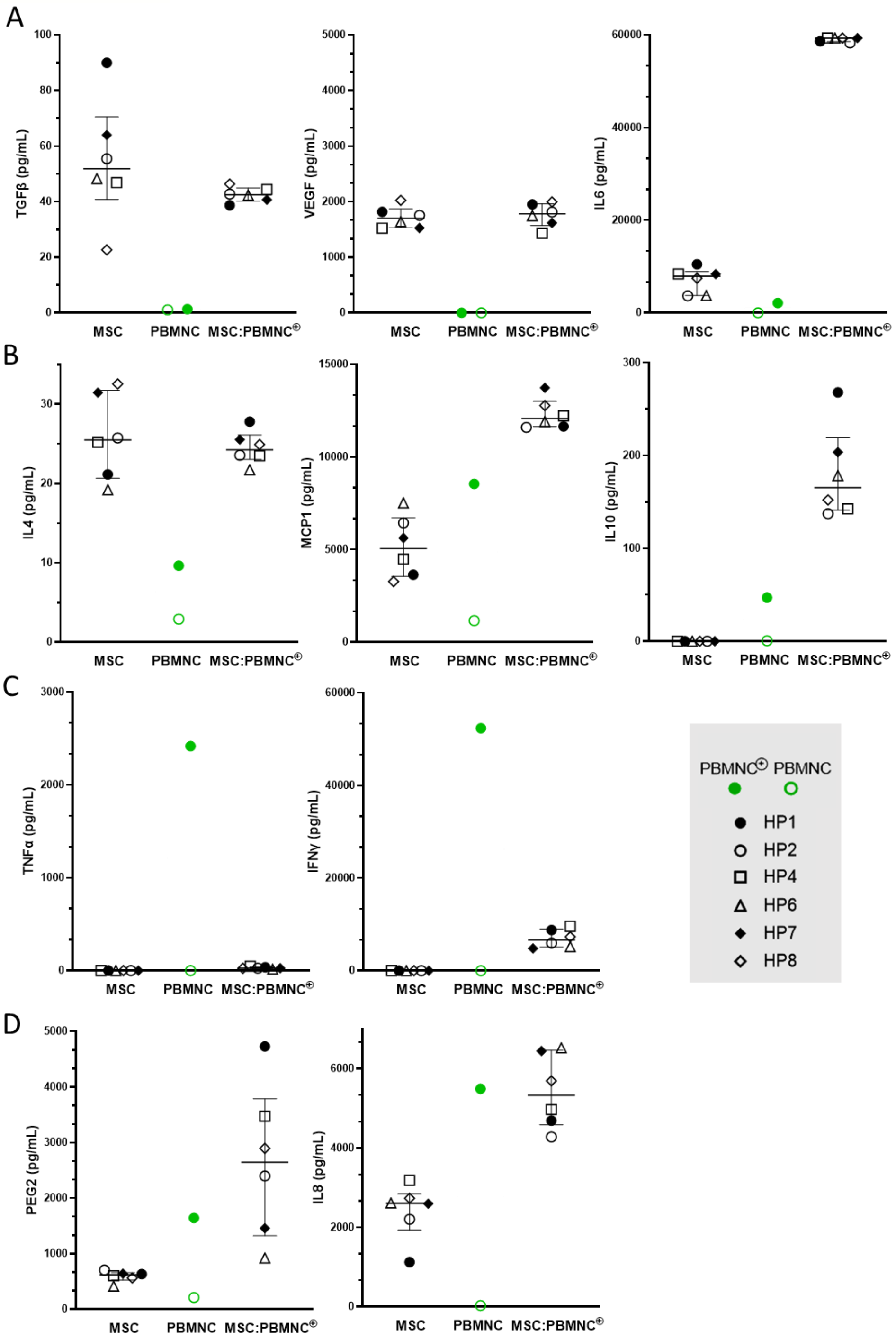

Supplementary Figure 2. Secretory profile of haploidentical BM-MSC in coculture. Immunomodulator levels were analysed in supernatants collected 3 days after initiating the cocultures of BM-MSC and activated PBMNC (ratio 1:5). Graphs display median values and IQR, with symbols representing the concentration for each batch **A.** Factors with unchanged undetectable levels after PBMNC activation that consistently increase in coculture with BM-MSCs across all batches. **B.** Factors that rise following PBMNC activation and homogeneously further increase in coculture with BM-MSCs across all batches. **C.** Factors that increase after PBMNC activation but consistently decrease in coculture with BM-MSCs, with TNF-α becoming undetectable. **D.** Factors that increase after PBMNC activation but exhibit heterogeneous changes (further increase or decrease) in coculture with BM-MSCs across batches.

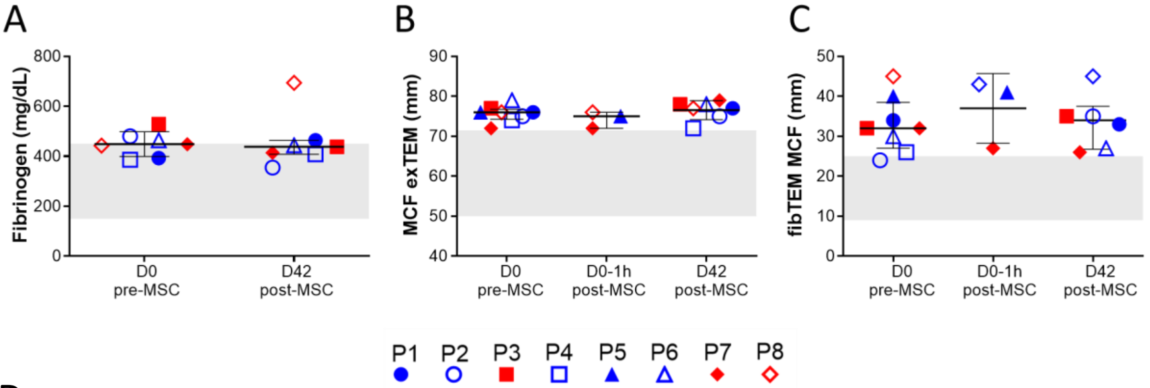

D.

Global		Median (IRQ) (platelets x 10^3/µL)			Difference between medians (CI ≈95%)		P One tailed	
		BASAL	SHORT TERM	LONG TERM	B vs ST	B vs LT	B vs ST	B vs LT
		435.5 (341.0; 525.8)	432.3 (393.0; 518.4)	420.5 (351.5; 526.3)	-3.25 (-98.0; 135.0)	-24.00 (-121.0; 87.50)	0.47	0.32
Individual	P1	286.5 (158; 415)	421.5 (370.5; 474.8)	374 (368; 380)	135 (-51; 324)	87.5 (-47; 222)	0.27	0.50
	P2	526 (521; 563)	527.5 (480.3; 624.3)	559 (421; 564)	1.5 (-87; 124)	33 (-142; 43)	0.43	0.50
	P3	659 (531; 703)	561 (553; 626.8)	538 (531; 700)	-98 (-151; 116)	-121 (-172; 169)	0.31	0.80
	P4	446 (435; 457)	385 (355.8; 413.5)	491 (458; 499)	**-61 (-104; -19)**	45 (1; 64)	0.07	0.10
	P5	319 (300; 359)	345.5 (332.8; 389.8)	288 (277; 299)	26.5 (-30; 104)	**-31 (-82; -1.00)**	0.20	0.10
	P6	525 (501; 570)	491 (438.5; 548)	451 (387; 529)	-34 (-135; 52)	-74 (-183; 28)	0.31	0.20
	P7	407 (284; 448)	443 (417.8; 480)	390 (354; 426)	36 (-35; 205)	-17 (-94; 142)	0.11	0.50
	P8	425 (412; 438)	417 (386; 442)	344 (302; 372)	-8.00 (-52; 30)	**-81 (-136; -40)**	0.50	0.10

Supplementary Figure 3: Coagulation study in RDEB patients following haploidentical BM-MSC systemic therapy. No global (Mann-Whitney U test) or individual differences (Wilcoxon test) reflecting alterations in the procoagulant profile or platelet count were found, regardless of whether patients were good (in blue) or mild responders (red). **A.** Plasma fibrinogen levels (Clauss method). **B-C.** Maximum clot firmness (MCF) assessed by rotational thromboelastometry (ROTEM) at short (D0, 1-hour post-infusion) and long (D42) time using exTEM (B) and fibTEM (C). Graphs show medians, interquartile ranges, the reference range (grey shading) and symbols representing individual patients. **D.** Platelet count remained normal throughout the study, suggesting that statistically significant differences observed in 3 patients (in bold) were not clinically relevant. Reference values according to La Paz University hospital (platelets x 10^3/µL): 1-12 years old: 180-490; 12-18 years old: 160-400 and >18 years old:150-370. Normal, Elevated, Decreased.

A

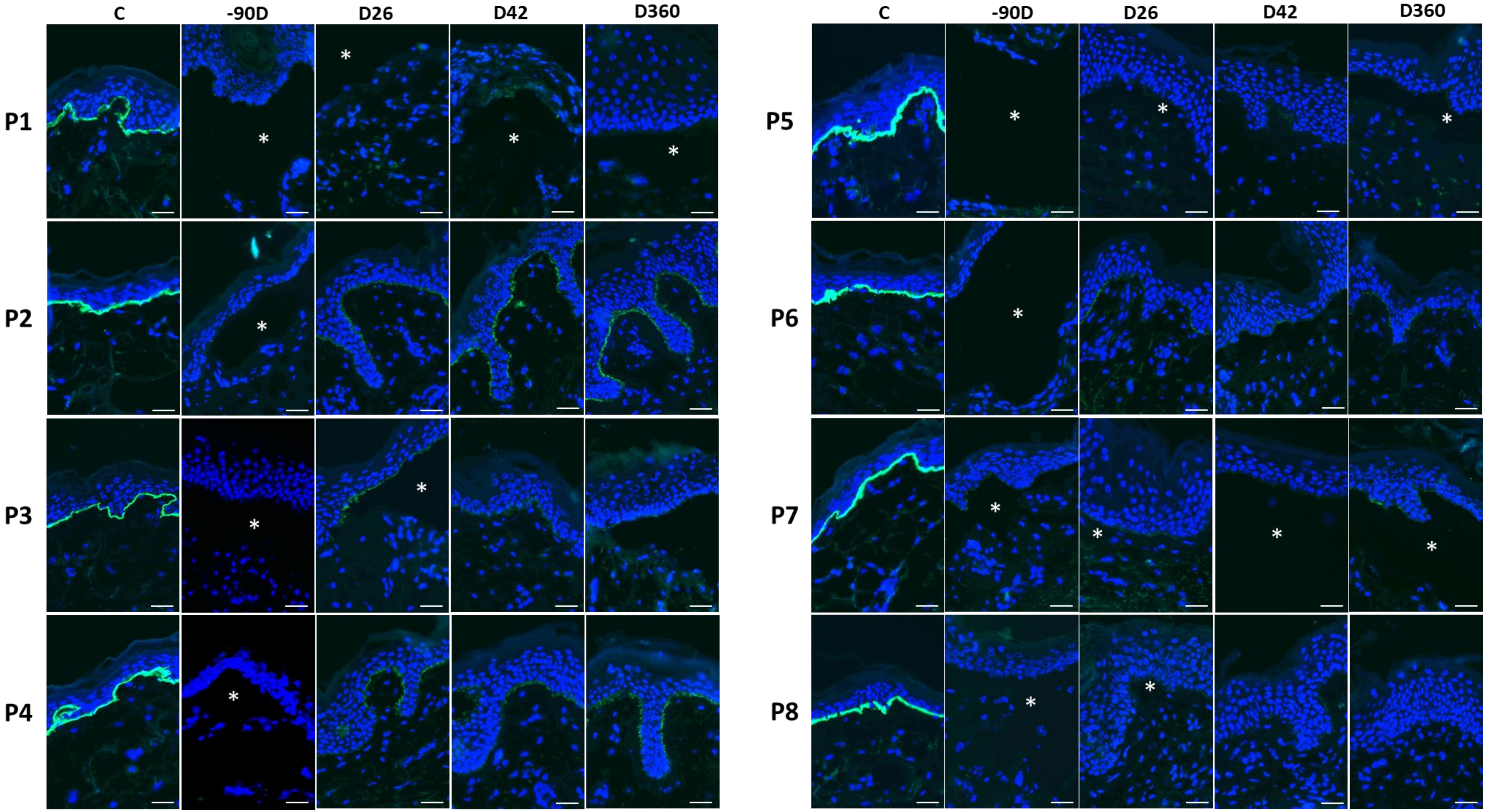

B

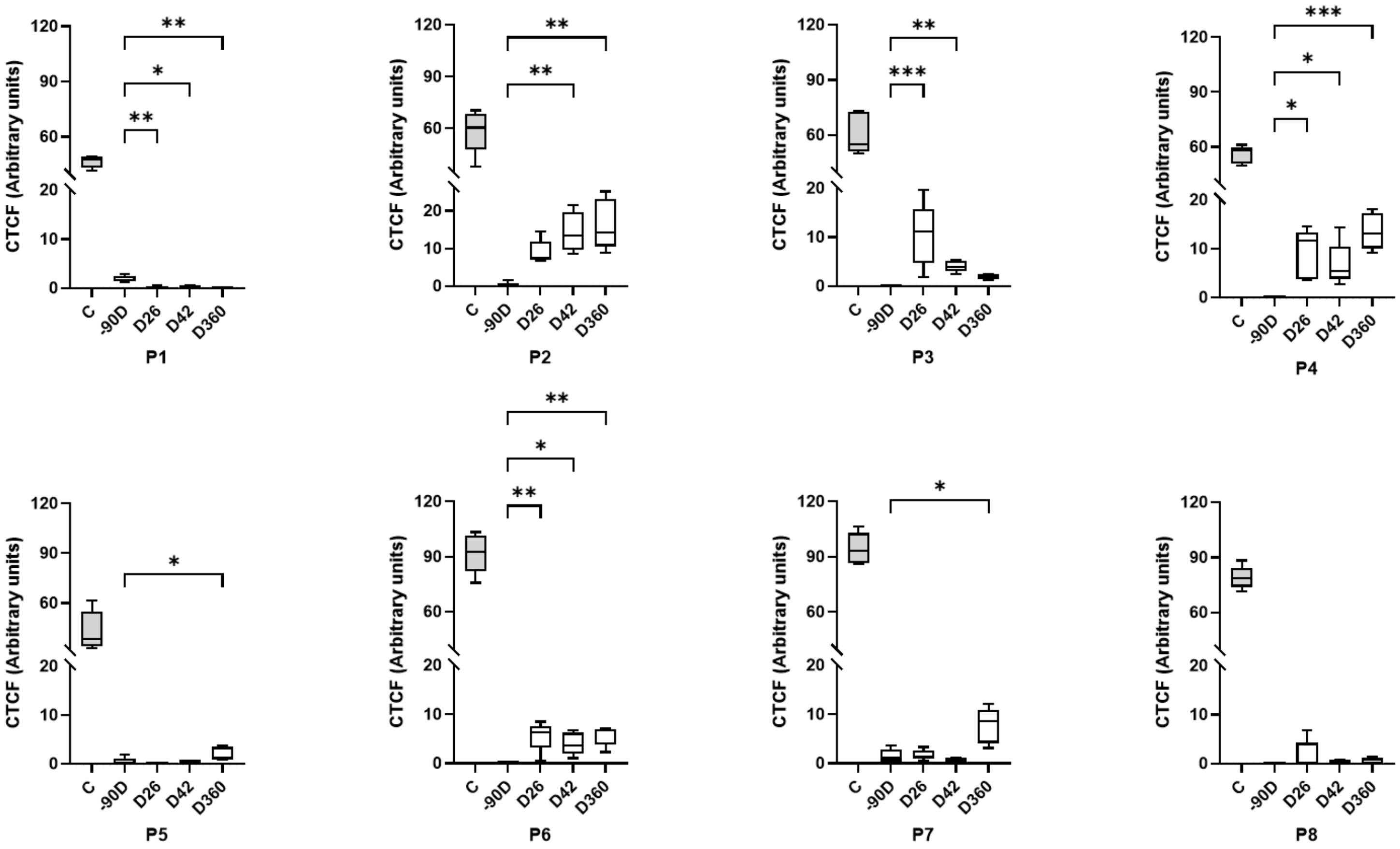

Supplementary Figure 4: C7 expression in RDEB patients following haploidentical BM-MSC systemic therapy. A. C7 was detected by indirect immunofluorescence using a monoclonal LH7.2 antibody (NC1 domain) on skin sections collected before (and after the treatment (D26, D42 and D360). **B.** Relative C7 expression was calculated from corrected total fluorescence (CTCF). White asterisks indicate blisters. Scale bars: 50 µm. Graphs show individual median values (IQR) from five random fields per sample. Statistical differences were calculated using the Kruskal-Wallis test followed by uncorrected Dunn's multiple comparisons test (*p ≤0.05, **p ≤ 0.01, ***p ≤0.001, ****p ≤0.0001).

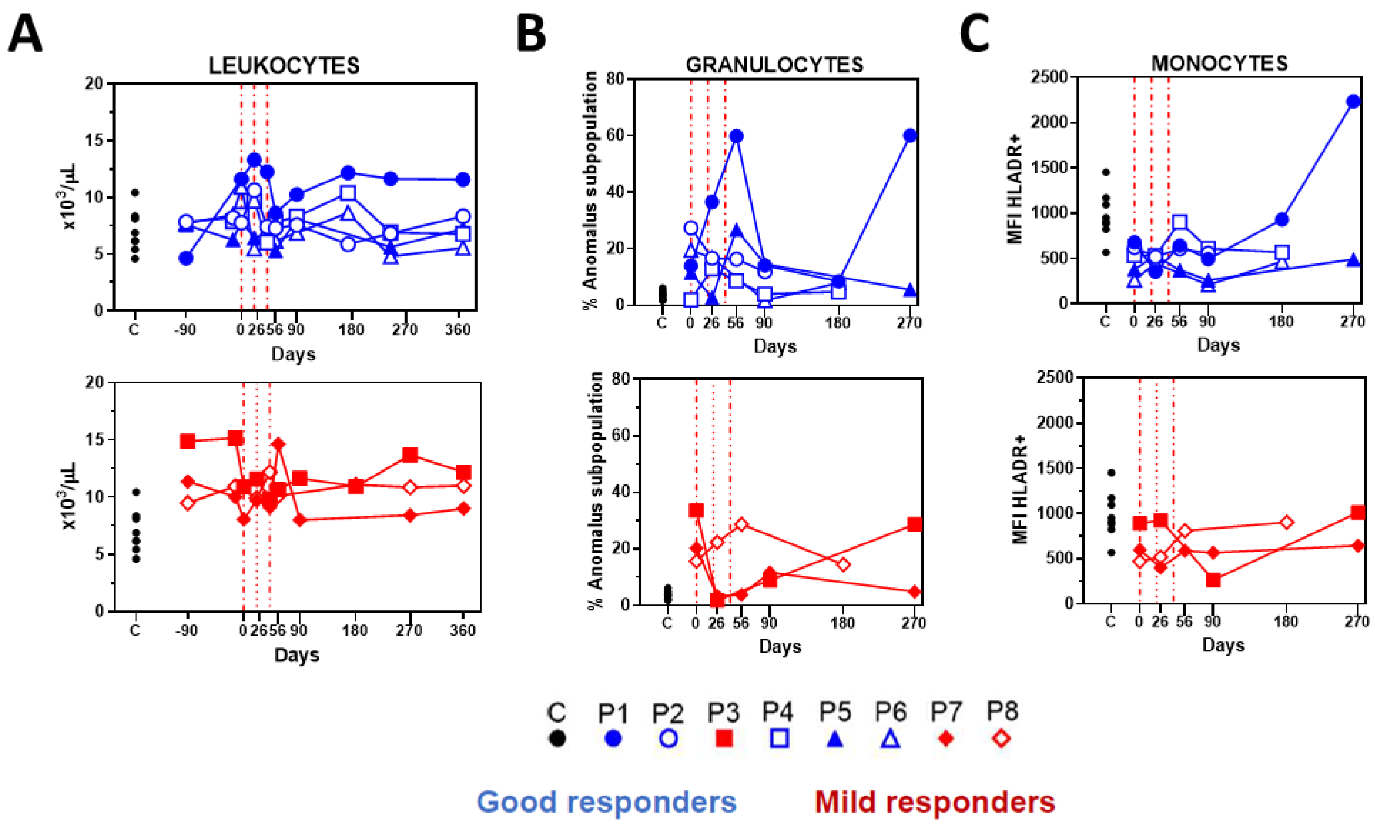

Supplementary Figure 5. Myeloid cell subpopulation dynamics in RDEB patients following haploidentical BM-MSC therapy, assessed by flow cytometry. Individual data for RDEB patients (P, n=8) and controls (C, n=8 unaffected individuals within the same age range). Vertical grids indicate treatment days. **A.** Baseline absolute leukocyte counts were generally lower in good responders (1/5 above normal) than in mild responders (2/3 above normal) and transiently normalized during follow-up. **B.** Baseline percentages of a larger anomalous granulocyte subpopulation (higher FSC) were elevated in all patients except P4 (1/5 good responders). This population transiently decreased or disappeared in all good responders and 2 mild responders (P3, P7). **C.** Monocyte HLA-DR/DQ mean fluorescence intensity (MFI, FSC/SSC gated). Half of the cohort had lower MFI than controls, with lowest values in P4, P5, P6 (good responders) and P8 (mild responder). Post-treatment fluctuations were not relevant.

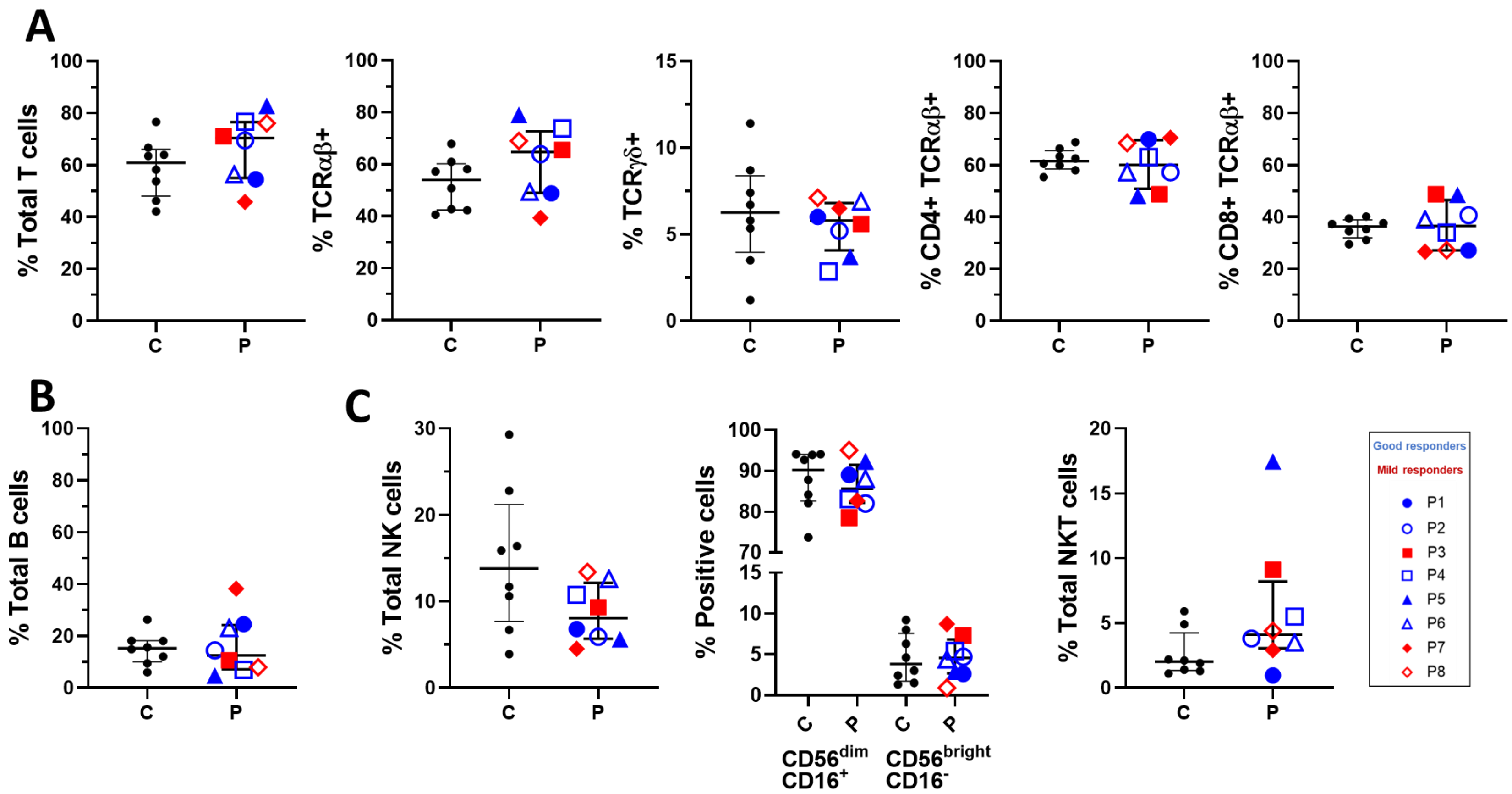

Supplementary Figure 6. Lymphoid cell populations at baseline in RDEB patients, assessed by flow cytometry. Individual data (symbols) and medians with interquartile ranges (bars) at day 0 in RDEB patients (P, n=8) and controls (C, n=8 unaffected individuals within the same age range). Outliers (1.5×IQR) were excluded. **A.** Percentages of circulating total T lymphocytes (CD3+) and TCRαβ (TCRγδ−), TCRγδ+, CD4 (CD4+TCRγδ−) or CD8 (CD4−TCRγδ−) subpopulations. **B.** Percentages of circulating total B lymphocytes (CD19+ cells) **C**. Percentages of circulating total NK cells (CD56+CD3−) and $CD56^{dim}$CD16+ or $CD56^{bright}$CD16- subpopulations **D.** Percentages of circulating total NKT cell (CD56+CD3+). No statistically significant differences were observed in these lymphoid subpopulations between patient and control medians (one-tailed unpaired Mann–Whitney U test). Notably P5 (a good responder) presented a proportion of NKT cells 7 times higher than the control mean before treatment.

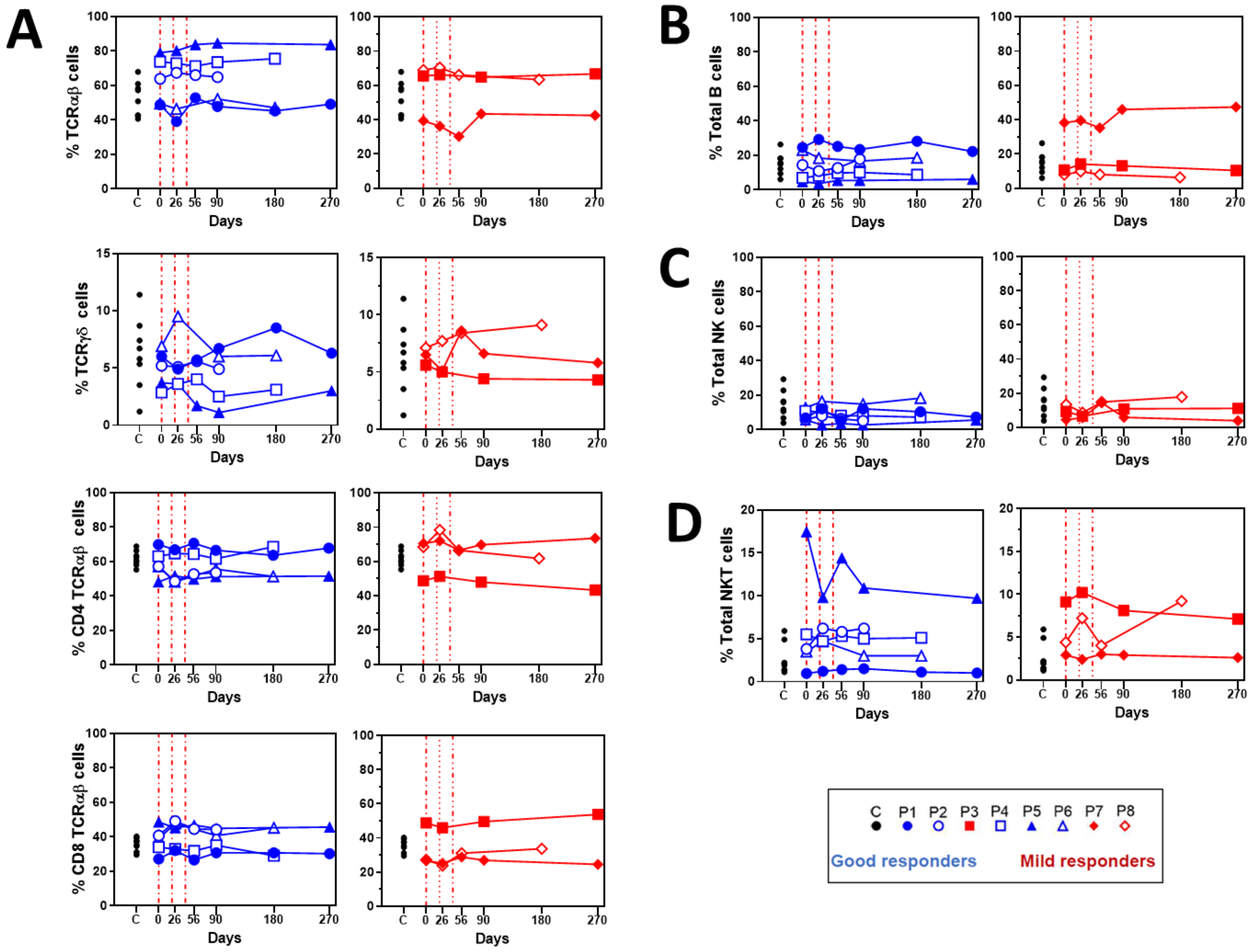

Supplementary Figure 7. Lymphoid cell population dynamics in RDEB patients following haploidentical BM-MSC therapy, assessed by flow cytometry. Individual data for RDEB patients (P, n=8) and controls (C, n=8 unaffected individuals within the same age range). Vertical grids indicate treatment days. **A.** Percentages of circulating TCRαβ (TCRγδ−), TCRγδ+, CD4 (CD4+TCRγδ−) or CD8 (CD4−TCRγδ−) subpopulations. **B.** Percentages of circulating total B lymphocytes (CD19+ cells) **C.** Percentages of circulating total NK cells (CD56+CD3−) and CD56^{dim}CD16+ or CD56^{bright}CD16- subpopulations **D.** Percentages of circulating total NKT cell (CD56+CD3+). In most cases, baseline percentages were comparable to controls and post-treatment fluctuations were not relevant. Notably, a consistent decrease in response to MSC was observed in the 7-fold elevated percentage of NKT in P5.

2. Supplementary Tables

Supplementary Table 1. Schedule of activities in MesenSistem-EB.

ACTIVITIES	VISIT: V<br>DAYS: D<br>Window	V0-V1<br>- 90D/- 15D	V2[1]<br>D0	V3[1]<br>D21	V4[1]<br>D42	V5<br>D56<br>+/-3D	V6<br>D90<br>+/-7D	V7<br>D180<br>+/-7D	V8<br>D270<br>+/-7D	V9<br>D360<br>+/-7D
Patient & Donor Informed consent		X								
Notification of eligibility		X								
Donor bone marrow aspiration		X								
Confirmation informed consent is available		X	X	X	X	X	X	X	X	X
Medical/personal history		X								
Physical exploration		X	X	X	X	X	X	X	X	X
Vital signs		X	X	X	X	X	X	X	X	X
Pregnancy test		X								
Birmingham epidermolysis severity score (BEBSS), percentage of body surface affected (BSA)		X	X	X	X	X	X	X	X	X
EB disease activity & scarring index (EBDASI)		X	X	X	X	X	X	X	X	X
Blood samples[2]	Genotyping COL7A1/HLA	X								
	α-C7 Autoantibodies (serum)	X				X				
	Mixed-lymphocyte culture	X								
	Blood test and inflammatory markers	X	X	X		X	X	X	X	X
Skin biopsy[2]	C7 expression IF	X				X				
	□-C7 Antibodies (at BMZ)	X				X				
	Primary culture & western blot	X								
	Electron microscopy	X						X		
	Dermal Chimerism							X		
Cutaneous resistance test (Suction blister time)		X				X		X		X
Photographs		X	X	X	X	X	X	X	X	X
Record of Adverse Events			X	X	X	X	X	X	X	X
Change of medication / dressings			X	X	X	X	X	X	X	X
Quality of life questionnaire (PedsQL)		X				X	X	X	X	X
Pain (VAS & Wong-Baker Faces)		X	X	X	X	X	X	X	X	X
Itching (Leuven Itch scale & Itchman).		X	X	X	X	X	X	X	X	X
Pre-treatment			X	X	X					
MSC infusion			X	X	X					

[1] Safety was also assessed by phone, 8 days (+/-2D) after every infusion.
[2] Remnants from diagnostic were used in V0-V2 when available

Supplementary Table 2. Recruitment criteria for haploidentical bone marrow donors

HUMAN LEUCOCYTE ANTIGEN (HLA) GENOTYPING [1]	CRITERIA
Antigens class I HLA-A, B and C<br>Antigens class II DRB1, DRB3/4/5 and DQB1	3/6 matching loci
**TEST FOR INFECTIOUS DISEASE**	**CRITERIA**
Hepatitis B virus (HBV) Surface Antigen (HBsAg)	Negative
Hepatitis C virus (HCV) Antibody testing	Negative
HVC Nucleic Acid Testing (NAT)	Negative
Human Immunodeficiency viruses, Types 1 and 2 (HIV 1,2) Antibody testing	Negative
Syphilis (Treponema pallidum)	Negative

[1]Analysis performed at Transfusion Center Autonomous Community of Madrid

Supplementary Table 3. Demographics of unaffected individuals

Control	SEX	AGE (years)	PATHOLOGY
**C1**	♀	5.6	Pilomatrixoma
**C2**	♀	10.7	Congenital melanocytic nevus
**C3**	♂	12.1	Congenital melanocytic nevus
**C4**	♀	14.4	Congenital melanocytic nevus
**C5**	♂	19.4	Without dematological pathology (standard pediatric health check)
**C6**	♀	6.9	Pilomatrixoma
**C7**	♀	8.0	Congenital melanocytic nevus
**C8**	♂	15.6	Without dematological pathology (standard pediatric health check)
**C9**	♂	18.0	Without dematological pathology (standard pediatric health check)

Sera from unaffected individuals of similar age range to the RDEB patients, attending the Dermatology Department at Hospital La Paz, were used to establish control levels for cytokines and other biomarkers not routinely analyzed.

Supplementary Table 4. Specifications, formulation and quality controls of the BM-MSC Investigational Medicinal Product

SPECIFICATION		ISCT CRITERIA[1]
Cellular passage		≤ 4
Cellular viability (Trypan Blue)		≥ 70%
In vitro differentiation in primary cultures: osteoblasts, adipocytes, chondroblasts		Positive staining for lineage specific markers
Immunophenotype CD73, CD90, CD105		≥ 95% positive
Immunophenotype CD45, CD34, CD14 /CD11b, CD79α /CD19 and HLA-DR		≤2% positive
**FORMULATION**		**QUALITY**
BM-MSC		GMP
30 ml Ringer Lactate		Registered
1% Human serum albumin		Registered
**QUALITY CONTROLS**	**METHOD**	**CRITERIA**
Initial Mycoplasma in collection medium	Luminescence	Negative
Total microbiological presence in supernatant	Sterility test (PH. Eur. 2.6.27) [2]	Absent
Total microbiological presence in final Lactated Ringer's wash	Sterility test (PH. Eur. 2.6.27) [2]	Absent

[1] Minimal standard criteria for defining multipotent mesenchymal stromal cells stablished by the International Society of Cellular Therapy ISTC (1) [2] European Pharmacopoeia (PH. Eur.) standards 2.6.27 Microbiological Examination of cell-based Preparations

Supplementary Table 5. Batch-Specific properties of the investigational medicinal product from haploidentical BM-MSC donors

DONOR Batch			HP1	HP2	HP3	HP4	HP5	HP6	HP7	HP8	HP9
BM aspirate		ml	120	140	125	120	120	115	120	115	115
Total, cell count (Coulter)		$x10^6$/ml	18.3	43.3	32.9	10.7	18.9	18.7	36.3	19.2	18.3
Mononucleated cell count		$x10^6$	485	1208	1025	270	635	510	561	282	290
No. Days passage 1			13	13	16	16	15	14	17	14	17
No. Cell passage 1		$x10^6$	68.7	212	7	31.6	27.2	80.6	13.5	7.9	41.4
Total No. Days			13	13	34	34	35	28	32	31	27
Total No. Cell passage			0	0	2	2	2	1	2	2	1
Total No. cells		$x10^6$	68.7	212	120	324	132.5	795	169	207	200
Duplication No.			2	0.8	5.5	4.4	3.3	4.7	4.8	6.1	2.3
SURFACE MARKERS	VEGFR2	%	40.9	45.6	31.8	22.9	22.89	27.5	42.9	59.5	32.4
	CD 71	%	85.6	59.4	78	29.7	59.3	86.5	73.8	78.6	79.9
	CD 31	%	8.6	0.5	2.9	0.4	4	2	0.5	0.6	0.4
	CD 106	%	93.3	71.3	55.4	38.5	49.4	73.1	59.0	62.4	62.2
	CD 184	%	49	45.8	34.1	11.9	45.5	51.3	64.9	50.1	56

frontiers

Supplementary Table 6. BM-MSC final product release data

DONOR BATCH	BM HARVESTING DATE (REGISTER) (DD/MM/YY)	INFUSION #	INFUSION DATE (DD/MM/YY)	DOSE/INFUSION CELLS (10^6) /KG	CUMULATIVE DOSE/INFUSION TOTAL, CELLS (10^6)	VIABILITY
**HP4**	06/11/2018	1st	20/12/2018	2.4	101	95
		2nd	10/01/2019	1.9	81	94.8
		3rd	31/01/2019	2.4	105	96
**HP2**	04/12/2018	1st	07/02/2019	2.9	67	93
		2nd	28/02/2019	2.4	58	98
		3rd	21/03/2019	2.6	60	93
**HP3**	08/01/2019	1st	18/02/2019	1.7	87	96.7
		2nd	11/03/2019	1.2	61.3	92.7
		3rd	01/04/2019	1.6	83.2	97
**HP1**	05/02/2019	1st	22/04/2019	2.8	43	93
		2nd	13/05/2019	2.8	44	92
		3rd	03/06/2019	2.9	44	95
**HP6**	01/04/2019	1st	10/06/2019	2.9	51	93
		2nd	01/07/2019	2.8	51	94
		3rd	22/07/2019	2.7	50	94
**HP7**	06/05/2019	1st	19/09/2019	2.9	62	94
		2nd	14/10/2019	2.8	62	93
		3rd	04/11/2019	1.9	42	91
**HP5**	03/06/2019	1st	07/10/2019	2.2	71	91
		2nd	28/10/2019	2.2	72	90
		3rd	18/11/2019	2.4	77	91
**HP8**	07/10/2019	1st	19/12/2019	2.9	57	93
		2nd	09/01/2020	2.9	57	92
		3rd	30/01/2020	2.9	57	94
**HP9**	04/11/2019	1st	09/03/2020	2.9	45	95
		2nd	POSTPONED DUE TO COVID-19 PANDEMIC.			
		3rd	OFF-LABEL ADMINISTRATION 20/09/2024			

Supplemental Table 7. Basal clinical features of the nine RDEB patient recruited in MesenSistem-EB

PATIENT		P1	P2	P3	P4	P5	P6	P7	P8	P9
V2	**Sex/age (V2)**	♀ 4y	♀ 10y	♂ 12y	♀ 14y	♂ 19y	♀ 6y	♀ 8y	♀5y	♂ 4y
	**Severity (acording to EBDASI)**	Severe	Severe	Severe	Severe	Severe	Severe	Severe	Severe	Severe
	**EBDASI (Activity+Damage) (V2)**	173(42+131)	176(38+138)	163(37+126)	153(25+128)	194(56+138)	119(14+105)	148(30+118)	176(43+133)	121 (24+97)
	**% skin afected (V2)**	42	44.5	49	31	45.5	17	36.5	39	20
	**Pseudosyndactily (Surgery)**	Yes (hands & feet)	Yes (Left hand)	Yes (hands & feet)	Yes (hands & feet)	Yes (hands & feet)	Synechiae	Yes	Yes	Synechiae
	**Anonychia (Nail dystrophy)**	Complete	Complete	Complete	Complete	Complete	Complete	Complete	Complete	Partial (Yes)
	**Ocular**	Xerophthalmia	Corneal ulcers	Xerophthalmia	Corneal ulcers	Severe corneal ulcers	No	No	Xerophthalmia	No
	**Oral Mucosa involvement**	Severe	Severe	Severe	Mild	Severe	Severe	Severe	Severe	Mod-Sev
	**Microstomy & Ankyloglosia**	Yes	Yes	Yes	Yes	Yes	Yes	Yes	Yes	Yes
	**Larinx**	Mild	No	No	No	Mild	No	No	No	No
	**Oesophageal Mucosa**	Severe	Severe	Mild	Moderate	Severe	No	Mild	Moderate	No
	**Dysphagia**	Occasional	Occasional	Occasional	Ocassional	Solid food	No	Ocassional	Ocassional	No
	**Stenosis (dilatation)**	Yes	No	No	Yes	Yes	No	No	No	No
	**Gastrostomy**	Yes	Yes	No	Yes	Yes	No	No	Yes	No
V0, V1 and V2	**Growth retardation:** Stunted, Wasted	Yes, No	Yes, Yes	No	Mildly Wasted	Yes, Yes	Yes, Yes	No	No	No
	**Anaemia (Hb)[1]**	Mild	**No**	**Moderate**	Mild	**Moderate**	**Moderate**	**No**	**Moderate**	**No**
	**Iron deficiency[2]**	**Yes**	**Yes**	**Yes**	**Yes**	**Yes**	**Yes**	**Yes**	**Yes**	**Yes**
	**Dyslipidemia[3]: Low HDL**	**Yes**	**Yes**	**Yes**	No	**Yes**	**Yes**	**Yes**	**Yes**	Yes
	**Dyslipidemia[3]: Hypocholesterolemia**	**Yes**	**No**	**No**	No	**Yes**	Yes	**No**	No	**No**
	**Dyslipidemia[3]: Hypotriglyceridemia**	**No**	**No**	**No**	Yes/No*	**Yes**	No	Yes	**No**	**No**
	**Hypovitaminosis[4]: Vit A**	Yes*	**Yes**	Yes*	Yes*	Yes*	**Yes**	**Yes**	Yes*	**Yes**
	**Hypovitaminosis[4]: Vit C**	Yes*	**No**	Yes*	No/Yes*	No/Yes*	**No**	**No**	No	**No**
	**Hypovitaminosis[4]: Vit D**	**Yes**	**Yes**	Yes*	Yes*	**Yes**	**No**	**Yes**	**No**	No
	**Inflammation (high levels): CRP[5]**	**Yes**	Yes	**Yes***	Yes*	**Yes***	**Yes**	**Yes**	**Yes***	**No**
	**Inflammation (high levels): IL6[6]**	**Yes**	**Yes**	**Yes**	Yes*	**Yes**	**Yes**	**Yes**	**Yes**	**Yes**
	**Inflammation (high levels): IL1b[6]**	**Yes**	**No**	**Yes**	Yes/No*	**No**	Yes	**No**	**Yes**	**No**
	**Inflammation (high levels): TNFa[6]**	**Yes**	**Yes**	**Yes**	Yes*	**Yes**	**Yes**	**Yes**	**Yes**	No
	**Inflammation (high levels): IgG*[7]**	Yes*	Yes*	No*	Yes*	Yes*	No*	Yes*	Yes*	No*
V2	**Itch Intensity Leuven (1-100)**	41.2	Intense (62.7)	37.5	43.8	Intense (62.5)	Very intense (86.2)	Intense (67.5)	56.2	50.0
	**Pain WB Faces (1-4)**	Moderate (4)	Severe (6)	No (0)	Mild (2)	Moderate (4)	Mild (2)	Mild (2)	Unbearable (10)	No (0)
	**Anxiety (depression)**	Yes	No	Yes (Yes)	No	Yes	No	No	No	No

Yes: at least two altered measurements; No: at least two normal measurements; Three altered measurements (in bold); *only two measurements available. [1] Anemia classification as defined by the World Health Organization. [2] Iron deficiency defined as Ferritin <100 ng/mL and TSI < 20%. [3] Hypocholesterolemia defined as total cholesterol < 120 mg/dL or LDL-cholesterol < 50 mg/dL; hypotriglyceridemia defined as triglycerides < 50 mg/dL. In all cases patients had at least two measurements under the normal rank. LDL was normal in all the patients (Low HDL < 50 mg/dL). [4]Hypovitaminosis as defined in supplemental table 13. [5]High levels of CRP compared with an age matched general population (>5 mg/dL); + indicates CRP levels > 50 mg/dL [6]High levels compared with an age-sex matched cohort. [7]High levels with an age matched general population (725-1900 mg/dL). Numerical values

Supplementary Table 8. Summary of emergent adverse events (AE) following haploidentical BM-MSC systemic therapy

TYPIFICATION OF ADVERSE EVENTS (AE)	N	%
Patients who presented AE	9	100
Total AE	127	100
**AE INTENSITY**		
Mild	124	97.6
Moderate	3	2.4
Severe (SAE)	0	0.0
**AE CAUSAL RELATIONSHIP WITH BM-MSC**		
Related	5	3.9
Not related	122	96.1
Expected	78	61.4
Unexpected	49	38.6
**EVOLUTION**		
Resolved	116	91.3
Improvement	5	3.9
Without changes	6	4.7
Deterioration	0	0.0
Death	0	0.0
**FREQUENCY**		
Single event	80	63.0
Recurrent event	25	19.7
Continous event	22	17.3
**ATTITUDE TOWARD STUDY MEDICATION**		
None	127	100
Abandonment	0	0.0
Interruption	0	0.0
Dose reduction	0	0.0
Start delay	0	0.0
**THERAPEUTIC INTERVENTION**		
Concomitant medication required	70	55.1

Supplementary Table 9. AEs by symptom type and relationship to MSC infusion

SYMPTOM TYPE	ADVERSE EVENT (AE)	PATIENTS (N)	AE (N)	BM-MSC RELATED		EXPECTED	
				YES	NO	YES	NO
**RESPIRATORY**	Rhinorrhea	2	**3**	0	3	3	0
	Dry cough	1	**2**	0	2	0	2
	Upper respiratory tract catarrh	2	**2**	0	2	0	2
**GENERAL**	Hypotensión	1	**1**	0	1	0	1
	General discomfort	1	**1**	0	1	0	1
**OPHTALMICS**	Eye stye	1	**1**	0	1	0	1
	Keratitis	1	**2**	0	2	0	2
	Conjunctivitis	1	**1**	0	1	1	0
**OTORHINOLARYNGOLOGICS**	Odynophagia	2	**5**	0	5	5	0
	Xerostomía	1	**1**	0	1	0	1
	Oral ulcers	1	**2**	0	2	0	2
**NUTRITION**	Iron deficency	1	**1**	0	1	0	1
	Iron deficency anemia	2	**2**	0	2	0	2
	Hypovitaminosis D	2	**2**	0	2	0	2
**NEUROLOGIC**	Headache	3	**5**	1	4	0	5
	Drowsiness	2	**2**	1	1	0	2
	Dizziness	1	**2**	0	2	0	2
**MUSCULOSKELETAL**	Pain	3	**6**	0	6	6	0
	Asthenia	4	**4**	1	3	0	4
	Arthritis	1	**1**	0	1	0	1
	Myalgias	2	**2**	0	2	2	0
**INFECTIOUS**	Fever	5	**23**	0	23	23	0
	Low fever	7	**19**	1	18	19	0
	Respiratory infection	1	**2**	0	2	2	0
	Vaginitis	1	**2**	0	2	0	2
	Cutaneous infection	5	**5**	0	5	5	0
	Cellulitis	2	**3**	0	3	0	3
	Cutaneous abcess	1	**1**	0	1	0	1
**GASTROINTESTINAL**	Abdominal pain	2	**3**	0	3	3	0
	Diarrhea	1	**1**	0	1	1	0
	Stomach flu	3	**4**	0	4	0	4
	Dysphagia	2	**2**	0	2	2	0
	Vomiting	2	**2**	1	1	2	0
**LLYMPH NODES**	Cervical adenopathy	1	**1**	0	1	1	0
**DERMATOLOGICAL**	Oral ulcers	1	**1**	0	1	1	0
	Wound exudation	2	**3**	0	3	0	3
	Schönlein-Henoch purple	1	**1**	0	1	0	1
	Atopic dermatitis outbreak	1	**1**	0	1	0	1
	Pruritus	1	**1**	0	1	1	0
**MOOD**	Low mood	1	**1**	0	1	0	1
**PROCEDURES**	Dentistry	1	**1**	0	1	0	1
	Esophageal dilation	1	**1**	0	1	1	0
	Percutaneous endoscopic gastrostomy	1	**1**	0	1	0	1

Supplementary Table 10. Vital signs

Parameter	Reference range	Median (IRQ)			Difference between medians (CI ≈95%)		Wilcoxon test (one tailed) P	
		BASAL	Short Term (ST)	Long Term (LT)	B vs ST	B vs LT	B vs ST	B vs LT
**Temperature (ºC)**	36.5-37	**36.7 (36.4; 37.0)**	**36.4 (36.1; 36.6)**	36.5 (36.2; 36.9)	**-0.4 (-0.6; -0.2)**	-0.1 (-0.9; 0.3)	**0.01**	0.24
**Blood pressure[1] (mmHg)**	Systolic (101-135)	115.5 (93.0; 121.8)	112.8 (93.5; 124.9)	108.0 (89.0; 116.8)	0.0 (-6.5; 11.0)	-6.0 (-23.0; 17.0)	0.41	0.10
	Diastolic (62-65)	61.5 (56.75; 67.0)	58.25 (56.0; 63.5)	57.5 (51.3; 67.3)	-1.0 (-10.5; 1.0)	-5.0 (-10.0; 10.0)	0.10	0.20
**Heart Rate (beats min^{-1})**	60-140	105.0 (83.5; 124.3)	107 (88.9; 118.4)	100.8 (89.0; 117.8)	-2.3 (-16.5; 27.5)	-1.3 (-22.0; 8.0)	0.470	0.32
**Respiratory Rate (breaths min^{-1})**	12-30	16.0 (12.5; 17)	14.0 (12.4; 14.0)	15 (12.5; 16.0)	-1.5 (-8.0; 2.0)	-1.5 (-8.0; 4.0)	0.06	0.34
**O_2 Saturation (%)**	95-100	98.8 (98.0; 100.0)	98.5 (98.0; 99.0)	98.8 (98.1; 100.0)	-0.3 (-1.5; 1.0)	0.0 (-2.0; 2.0)	0.20	0.450

Measurements were taken under 8h fasting conditions. **Basal status (B):** V1 (-90 days), V2 (-15 days), V3 (day 0), **Short term (ST):** V4 (day 21), V5 (day 42), V6 (day 56), V7(day 90); **Long term (LT):** V7 (day 180), V8 (day 270), V9 (day 360).[1]Reference values for children 4-17 years old according to the European Society of Hypertension (2). [2]Reference values for children 4-17 years old according to Fleming et al., 2011. https://ars.els-cdn.com/content/image/1-s2.0-S014067361062226X-mmc1.pdf

Supplementary Table 11. Autoantibodies following haploidentical BM-MSC systemic therapy

Code	PRE-TREATMENT		POST-TREATMENT (V5[2])	
	Circulating [1] (ELISA)	Deposited (IIF)	Circulating [1] (ELISA)	deposited (IFI)
**P1**	9 U/ml	No	121 RU/ml	No
**P2**	8 U/ml	No	15 RU/ml	No
**P3**	5 U/ml	No	7 RU/ml	No
**P4**	3 U/ml	No	5 U/ml	No
**P5**	1 U/ml	No	4 RU/ml	No
**P6**	5 U/ml	No	6 RU/ml	No
**P7**	3 U/ml	No	6 RU/ml	No
**P8**	4 RU/ml	No	19 RU/ml	No

[1]Values above 6 U/ml or 20 RU/ml are considered positive (in red), according to the Immunodermatology Laboratory at St John's Institute of Dermatology (London, UK).

Supplementary Table 12. Global analysis of the clinical benefits following haploidentical BM-MSC systemic therapy

			Median (IRQ)			Difference between medians (CI ≈95%)		Wilcoxon test (one tailed) P	
			**BASAL**	**SHORT TERM**	**LONG TERM**	**B vs ST**	**B vs LT**	**B vs ST**	**B vs LT**
**MUCO-CUTANEOUS**	**BEBSS** (0-100)		39.9 (31.9; 47.9)	40.3 (29.9; 45.6)	43.8 (32.9; 44.9)	-0.8 (-6.4; 1.4)	0.2 (-3.5; 3.8)	0.10	0.38
	**BSA** (%)		38.2 (32.8; 44.1)	38.1 (22.1; 42.8)	37.2 (28.6; 40.5)	-1.5 (-12.8; 2.8)	-1.0 (-7.0; 5.5)	0.11	0.34
	**EBDASI** (SCORE, points)	Activity (0-276)	36.5 (27.8; 41.8)	35.2 (21.2; 43.9)	29.0 (27.0; 47.0)	-1.8 (-12.0; 4.0)	-4.0 (-14.0; 14.0)	0.17	0.23
		Damage (0-230)	128.0 (120.4; 137.0)	128.0 (115.8; 134.6)	131.0 (119.8; 136.8)	-2.0 (-13.0; 2.0)	-1.0 (-8.5; 12.0)	0.06	0.38
		Total (0-506)	164.5 (149.6; 177.5)	163.3 (137.9; 178.3)	167.0 (146.3; 181.3)	-2.2 (-23.0; 9.0)	-4.8 (-11.0; 13.0)	0.10	0.27
	**SKIN RESISTENCE,** Blister time (NP2, 1745 ± 476.12 sec)		868.5 (603.4; 1040.0)	748.1 (669.2; 1013.0)	614.7 (504.7; 904.7)	-63.8 (-482.0; 292.5)	-6.6 (-560.7; 164.0)	0.23	0.23
	**SKIN C7 EXPRESSION** (IF, CTCF arbitrary units %)		**0.1 (0.0; 0.9)**	**3.7 (0.2; 10.2)**	**5.6 (1.6; 19.3)**	3.2 (-3.5; 16.2)	5.6 (-3.5; 23.2)	**0.04**	**0.02**
**INFLAMMATORY**[1]	**CRP** (≤ 5 mg/L)		20.9 (12.6; 97.7)	26.6 (14.5; 70.2)	20.4 (15.0; 65.8)	-1.4 (-35.7; 11.5)	0.5 (-41.4; 15.3)	0.37	0.37
	**FIBRINOGEN** (150-450 mg/dL)		518.3 (434.5; 601.0)	510.3 (442.5; 581.8)	519.5 (433.5; 588.5)	0.2 (-85.0; 43.0)	2.5 (-131.0;164.0)	0.42	0.42
	**PREALBUMIN** (20-40 mg/dL)		12.7 (11.6; 13.9)	12.4 (11.4; 13.8)	12.0 (10.9; 13.7)	-0.1 (-1.0; 1.5)	-0.4 (-1.7; 1.0)	0.42	0.19
	**RETINOL BINDING PROTEIN** (3-6 mg/dL)		2.1 (1.8; 2.3)	2.05 (2.0; 2.2)	2.2 (1.9; 2.4)	0.02 (-0.25; 0.23)	0.1 (-0.3; 0.5)	0.42	0.19
	**LEUKOCYTES** (4.80-15.00 x103/µl)		8.3 (7.9; 10.2)	8.9 (6.8; 10.9)	7.8 (6.5; 11.5)	-0.5 (-3.8; 3.1)	-1.2 (-2.9; 3.5)	0.13	0.13
**PAIN**	**WONG BAKER FACES** SCORES (0-10)	BEFORE CURE & DRESSING	2 (0.5; 5)	2 (1.3; 2.8)	2 (0.5; 3.8)	0 (-4; 3)	0 (-2; 1)	0.31	0.50
		AFTER CURE & DRESSING	3 (0.5; 4)	2 (2; 3.5)	2 (0.5; 3.5)	0 (-3; 2)	0 (-3; 4)	0.38	0.50
	**VAS PAIN** SCORES (0-10 points)	BEFORE CURE & DRESSING	3 (2; 5.2)	2.5 (2; 4)	3 (2; 4)	0 (-2; 0)	0 (-1.5; 0)	0.23	0.25
		AFTER CURE & DRESSING	3 (2.5; 7)	2 (2; 4.2)	2 (2; 4.5)	-1 (-4; 0)	-1 (-4; 0)	0.23	0.63
**PRURITUS**	**LEUVEN ITCH SCALE** (SCORE, points)	**FRECUENCY (0-100)**	**75 (75; 9.8)**	**75 (50; 75)**	**75 (53.1; 75)**	**-25 (-37; 0)**	**-12.5 (-25; 0)**	**0.03**	**0.03**
		DURATION (0-100)	24.8 (0.0; 33.0)	16.5 (0.0; 33.0)	24.8 (0.0; 33.0)	0.0 (-33.3; 17.0)	0.0 (-16.5; 16.5)	0.50	0.50
		SEVERITY (0-100 %)	53.8 (40.0; 64.4)	44.4 (17.7; 59.4)	62.5 (34.7; 65.0)	-5.3 (-41.5; 15.6)	1.2 (-32.5; 21.2)	0.10	0.42
		DISTRESS (0-100)	48.8 (38.1; 61.9)	47.2 (32.5; 65.9)	41.2 (12.1; 68.1)	0.02 (-21.2; 14.4)	-7.5 (-35; 13.8)	0.42	0.11
		CONSEQUENCES (0-100)	23.9 (18.8; 46.6)	27.9 (15.9; 43.1)	28.4 (12.53; 55.1)	-4.0 (-15.9; 14.3)	2.2 (-13.6; 2)	0.24	0.36
		SURFACE (0-100)	11.5 (7.0; 23.9)	12.2 (8.5; 19.6)	13.0 (8.8; 22.0)	-0.1 (-12.5; 14.8)	2.8 (-14.0; 17.0)	0.44	0.47
	**ITCHMAN** SCORES (0-10 points)		2 (1; 3)	1.5 (0.5; 3)	2.5 (1; 3.2)	0 (-1.5; 0.5)	0 (-1.00; 1.00)	0.19	0.50

Basal (B) medians from V0 (-90D), V1 (-15D), and V2 (D0); Short Term (ST) medians from V3 (D21), V4 (D42), V5 (D56) and V6 (D90) and Long Term (LT) medians from V7 (D180), V8 (D270), and V9 (D360). D= days.

[1]Reference values according to La Paz University hospital reference ranks: Normal, Elevated, Decreased

Statistically Significant differences: p ≤0.05 (in bold).

Supplementary Table 13. Global analysis of changes in the nutritional status following haploidentical BM-MSC systemic therapy

		Median (IRQ)			Difference between medians (CI ≈95%)		Wilcoxon test (one tailed) P	
		BASAL	SHORT TERM	LONG TERM	B vs ST	B vs LT	B vs ST	B vs LT
GROWTH	**Height cm**	**123.5 (106.8; 152.0)**	**124.8 (108.5; 152.6)**	**128.8 (112.8; 155.8)**	1,0 (-0,5; 2,3)	**4,5 (1,5; 7,5)**	**0,012**	**0,004**
	**Weight Kg**	**22.0 (17.8; 39.9)**	**22.9 (18.8; 40.7)**	**25.1 (20.5; 43.7)**	0,8 (-0,4; 1,1)	2,60 (-1,00; 4,50)	**0,012**	**0,012**
	**BODY MASS INDEX**	**10.3 (9.18; 11.55)**	**9.5 (9.09; 11.16)**	**10.7 (9.23; 11.6)**	0.3 (-0.2; 0.8)	0.3 (-1.3; 1.4)	**0.02**	0.07
ANEMIA	**HEMOGLOBIN**[1] (10.2-17.2 g/dL)	10.3 (9.2; 11.6)	9.5 (9.1; 11.2)	10.7 (9.2; 11.6)	-0.5 (-1.0; 0.4)	0.5 (-1.4; 1.2)	**0.04**	0.31
	**TRANSFERRIN** (250-380 mg/dL)	239.5 (198.3; 283.0)	222.0 (210.0; 269.0)	220.0 (196.1; 264.5)	-0.3 (-54.5; 16.0)	-14.5 (-62.0; 27.0)	0.37	0.7
	**SERUM IRON** (50-175mg/dL)	16.0 (9.2; 27.2)	17.8 (15.6; 27.25)	27.5 (15.2; 36.2)	0.5 (-9.0; 9.0)	5.5 (-4.0; 22.0)	0.23	**0.03**
	**FERRITIN** (10-322 ng/ml)	16.8 (10.2;30.5)	17.5 (7.9;30.2)	21 (10.8; 56.5)	0.5 (-11.5; 1.0)	6.0 (-17.0; 40.0)	0.46	0.13
	**HEPCIDIN**[2] (11.4-22.4ng/ml)	18.8 (14.7; 23.6)	11.4 (6.9; 17.9)	ND	-7.5 (-18.4; 4.9)	NA	**0.02**	ND
	**ERYTHROPOIETIN** [5.68 mU/ml (5.03; 10.67)]	26.0 (15.0; 86.7)	25.8 (18.0; 61.3)	ND	-7.7 (-31.4; 10.8)	NA	0.16	ND
	**Transferrin saturation index (15-50%)**	4 (3; 9,5)	5,25 (4,125; 9,125)	8 (5,125; 10,75)	0,75 (-1,5; 2,0)	2,25 (-1.0; 7.0)	0,125	**0,02**
PROTEIN METABOLISM	**ALBUMIN** [1] (2.9-5.4 mg/dL)	3.8 (3.5; 4.0)	3.7 (3.6; 3.9)	3.8 (3.6; 4.0)	-0.1 (-0.2; 0.1)	0.0 (-0.2;0.1)	0.07	0.38
	**PREALBUMIN** (20-40 mg/dL)	12.7 (11.6; 13.9)	12.35 (11.4; 13.8)	12 (10.9; 13.7)	-0.1 (-1.0; 1.5)	-0.4 (-1.7; 1.0)	0.42	0.19
	**PROTEÍNAS TOTALES** [3] (6– 8.3 g/dL)	**7.4 (7.2; 8.0)**	**7.2 (6.9; 7.9)**	**7.4 (7.0; 7.8)**	-0.2 (-0.8; 0.2)	-0.2 (-0.6; 0.1)	**0.03**	**0.04**
	**UREA** (11-49mg/dL)	24.5 (20.5; 26.5)	24.0 (20.6; 28.8)	24.5 (29.4; 29.8)	0.2 (-2.00; 4.5)	-0.50 (-4.5; 12.5)	0.17	0.33
	**CREATININ** (0.3-1.3 mg/dL)	0.3 (0.2; 0.4)	0.3 (0.2; 0.4)	0.3 (0.2; 0.4)	-0.01 (-0.16; 0.03)	-0.01 (-0.08; 0.07)	0.30	0.50
LIPID METABOLISM	**Triglycerides** (50-150 mg/dL)	65.5 (48.1; 87.5)	59.8 (45.6; 90.0)	64.0 (45.1; 97.0)	-0.5 (-28.0; 18.0)	1.2 (-30.0; 44.0)	0.35	0.37
	**Cholesterol Total** (120-200 mg/dL)	130.5 (110.5; 155.1)	122.8 (109.5; 153.9)	129.0 (115.0; 151.1)	-5.0 (-13.5; 14.0)	0.0 (-15.5; 22.0)	0.13	0.5
	**HDL Cholesterol** (35-85 mg/dL) (>50)	36.0 (31.2; 48.2	32.8 (28.8; 47.9)	31.0 (31.1; 48.8)	-2.0 (-4.0; 2.5)	-0.8 (-6.0; 3.0)	0.05	0.12
	**LDL Cholesterol** (50-130 mg/dL)	83.0 (63.0; 100.0)	78.0 (67.5; 105.0)	85.0 (72.0; 96.0)	-5.0 (-11.5; 12.0)	1.0 (-15.5; 12.0)	0.39	0.47
VITAMINS	**Vit A** (0.30-0.50 ug/mL)	0.2 (0.2; 0.2)	0.2 (0.2; 0.3)	0.2 (0.2; 0.3)	0.01 (-0.03; 0.08)	0.02 (-0.04; 0.05)	0.11	0.15
	**Vit 25(OH) D3** (30-100 ng/mL)	25.8 (20.6; 29.8)	26.2 (21.2; 28.5)	25.2 (19.5; 32.4)	-1.8 (-7.5; 8.0)	0.5 (-12.5; 8.5)	0.38	0.43
	**Vit E** (6.30-14.20 ug/mL)	12.6 (11.5; 11.3)	12.8 (11.3; 14.7)	12.7 (12.1; 18.5)	0.1 (-1.5; 3.6)	1.8 (-1.9; 7.2)	0.27	0.06
	**Vit C** (34-113 µmol/L)	**34.7 (21.9; 44.8)**	**47.0 (43.6; 74.9)**	**49.6 (39.5; 68.6)**	**20.1 (6.6; 36.9)**	12.7 (-1.5; 31.5)	**0.004**	0.05
	**Vit B12** (211-911 pg/mL)	754.5 (628.5; 914.9)	776.3 (608.8; 883.1)	758.3 (638.0; 928.0)	22.8 (-138.0; 101.0)	52.2 (-239.0; 184.5)	0.37	0.47
	**Vit B9/Folate** (>2.6-17 ng/mL)	15.2 (10.0; 19.70)	15.80 (10.90; 22.2)	18.4 (13.6; 20.5)	0.6 (-1.2; 4.0)	2.7 (-3.3; 6.3)	0.11	0.07
TRACE ELEMENTS	**ZINC** (670-1200 µg/L)	805.5 (726.3; 917.0)	841.5 (749.1; 1039.0)	782.5 (673.3; 1074.0)	58.2 (-237.0; 279.0)	-21.0 (-158.0; 299.0)	0.19	0.42
	**SELENIUM** (65-120 µg/L)	77.0 (68.2; 89.4)	73.8 (70.1; 85.9)	71.5 (64.6; 84.4)	-3.5 (-6.5; 5.0)	-4.5 (-9.0; 6.0)	0.32	**0.04**

Reference values according to La Paz University hospital reference ranks: Normal, Elevated, Decreased. [1]Varies with age. Hemoglobin: 10.1 - 14.6 g/dL (0-12 years old), 12.5 - 16.6 g/dL (12-18 years old) and 13.5 - 17.2 g/dL (>18 years old). Albumin: 3.8 - 5.4 mg/dL (0-14 years old) and 2.9 - 5.2 mg/dL (>14 years old). Total protein: 6 – 8 g/dL (0-12 years old) and 6.4 – 8.3 g/dL (>12 years old). Creatinin: 0.3 - 0.7 mg/dL (0-12 years old), 0.5 - 1.0 mg/dL (12-18 years old), 0.5 - 1.1 mg/dL (>18 females) and 0.7 - 1.3 mg/dL (>18 males); [2]Control rank in a cohort of 8 unaffected individuals aged 5-19 years old. Hypocholesterolemia defined as total cholesterol < 120 mg/dL or LDL-cholesterol < 50 mg/dL; hypotriglyceridemia defined as triglycerides < 50 mg/dL. **Basal status (B):** V1 (- 3 months), V2 (-15 days), V3 (day 0), **Short term (ST):** V4 (day 21), V5 (day 42), V6 (day 56), V7(day 90); **Long term (LT):** V7 (day 180), V8 (day 270), V9 (day 360).

Supplementary Table 14. Individual analysis of changes in pruritus by LIS 1.0 following haploidentical BM-MSC systemic therapy

		Median (IRQ)			Difference between medians (CI ≈95%)		Wilcoxon test (one tailed) P	
	Code	BASAL	SHORT TERM	LONG TERM	B vs ST	B vs LT	B vs ST	B vs LT
FRECUENCY (0-100)	P1	75 (75; 100)	50 (31.25; 68.75)	75 (50; 75)	-25 (-75; 0)	0 (-50; 0)	0.09	0.30
	P2	100 (50; 100)	75 (75; 93.85)	75 (75; 75)	-25 (-25; 50)	-25 (-25; 25)	0.37	0.20
	P3	75 (50; 75)	50 (50; 50)	50 (50; 50)	-25 (-25; 0)	-25 (-25; 0)	0.14	0.20
	P4	62.5 (50; 75)	25 (6.3; 62.5)	50 (25; 75)	-37.5 (-75; 25)	-12.5 (-50; 25)	0.20	0.50
	P5	75 (75; 75)	75 (75; 75)	62.5 (50; 75)	0 (0; 0)	-12.5 (-25; 0)	0.50	0.40
	P6	75 (75; 100)	75 (56.25; 75)	75 (50; 75)	0 (-50; 0)	0 (-50; 0)	0.29	0.30
	P7	75 (75; 75)	75 (75; 75)	75 (75; 75)	0 (0; 0)	0 (0; 0)	0.50	0.50
	P8	100 (75; 100)	75 (75; 75)	75 (75; 75)	-25 (-25; 0)	-25 (-25; 0)	0.14	0.20
DURATION (0-100)	**P1**	**33 (33; 33)**	**0 (0; 0)**	33 (0; 33)	**-33 (-33; -33)**	0 (-33; 0)	**0.03**	0.50
	P2	33 (33; 100)	50 (33; 67)	33 (0; 33)	17 (-67; 34)	0 (-100; 0)	0.50	0.30
	P3	33 (33; 33)	33 (8.5; 33)	33 (33; 33)	0 (-33; 0)	0 (0; 0)	0.57	0.50
	P4	16.5 (0; 33)	0 (0; 33)	0 (0; 33)	0 (-33; 33)	-16.5 (-33; 33)	0.70	0.70
	P5	0 (0; 33)	16.5 (0; 33)	16.5 (0; 33)	16.5 (-33; 33)	16.5 (-33; 33)	0.63	0.70
	P6	0 (0; 67)	0 (0; 0)	0 (0; 33)	0 (-67; 0)	0 (0; 33)	0.43	0.50
	P7	0 (0; 33)	16.5 (0; 33)	0 (0; 0)	16.5 (-33; 33)	0 (-33; 0)	0.63	0.50
	P8	33 (0 33)	33 (33; 33)	33 (33; 33)	0 (0; 33)	0 (0; 33)	0.43	0.50
SEVERITY (0-100)	P1	**57.5 (41.2; 67.5)**	**15.0 (10.94; 24.69)**	32.5 (22.5; 50)	**-42.5 (-57.5; -13.8)**	-25.0 (-45.0; 8.7)	**0.03**	0.10
	P2	50.0 (43.8; 62.7)	55.0 (46.6; 6.4)	65.0 (62.5; 68.8)	5 (-16.4; 20)	15.0 (-0.2; 25.0)	0.43	0.10
	P3	37.5 (30.0; 53.8)	33.8 (27.2; 58.1)	41.2 (32.5; 6)	-3.8 (-27.5; 3)	3.8 (-21.2; 2)	0.43	0.35
	P4	38.8 (33.8; 43.8)	9.4 (3.8; 39.4)	6.2 (3.7; 6)	-29.4 (-40.0; 13.8)	-32.8 (-40.0; 21.8)	0.27	0.40
	P5	43.75 (37.5; 62.5)	59.38 (52.19; 60)	65 (58.75; 71.25)	15.63 (-12.5; 22.5)	21.25 (-3.75; 33.75)	0.26	0.20
	P6	62.5 (47.5; 86.2)	25.6 (3.8; 67.2)	62.5 (12.5; 67.5)	-36.9 (-83.8; 27.5)	0.0 (-73.8; 20.0)	0.11	0.40
	P7	66.2 (53.8; 67.5)	59.4 (46.9; 65.3)	62.5 (57.5; 66.2)	-6.9 (-23.8; 12.5)	-3.8 (-10.0; 12.5)	0.23	0.40
	P8	65.0 (56.2; 77.5)	63.1 (56.2; 69.1)	67.5 (65.0; 70.0)	-1.9 (-22.8; 13.8)	2.5 (-12.5; 13.8)	0.43	0.40
DISTRESS (0-100)	P1	50.0 (45.0; 66.3)	28.8 (16.2; 46.0)	40.0 (13.8; 58.8)	-21.2 (-51.3; 3.8)	-10 (-52.5; 13.8)	0.06	0.20
	P2	66.2 (56.3; 68.8)	70 (62.23; 79.73)	70 (68.8; 70)	3.8 (-7.5; 25)	3.8 (0; 13.7)	0.31	0.10
	P3	47.5 (40; 52.5)	43.75 (35.35; 51.25)	42.5 (38.8; 52.5)	-3.75 (-18.7; 12.5)	-5 (-13.7; 12.5)	0.43	0.40
	P4	21.9 (5; 38.8)	6.3 (3.45; 36.3)	6.3 (5; 63.8)	-15.6 (-36.3; 41.3)	-15.6 (-33.8; 58.8)	0.50	0.50
	P5	40.0 (25.0; 50.0)	53.8 (46.0; 60.6)	58.8 (58.8; 58.8)	13.8 (-6.2; 37.5)	NA	0.57	NA
	P6	37.5 (0.0; 50)	43.8 (6.2; 86.0)	2.5 (0.0; 71.3)	6.2 (-50.0; 93.8)	-35.0 (-50.0; 71.3)	0.37	0.50
	P7	62.5 (43.8; 66.3)	50.65 (29.38; 68.18)	62.5 (60; 66.7)	-11.85 (-43.8; 30)	0 (-6.3; 22.5)	0.43	0.50
	P8	**60.0 (53.8; 62.5)**	**74.4 (68.9; 76.0)**	73.8 (65.0; 75.0)	14.4 (3.8; 22.5)	13.8 (2.5; 21.2)	**0.03**	5.00
CONSEQUENCES (0-100)	**P1**	**56.8 (52.1; 63.6)**	**45.4 (43.2; 49.4)**	61.4 (56.8; 63.6)	**-11.4 (-20.4; -2.1)**	4.6 (-6.8; 11.5)	**0.03**	0.40
	P2	36.4 (36.4; 45.6)	37.5 (31.2; 45.4)	34.1 (29.5; 34.1)	1.1 (-16; 11.3)	-2.3 (-16; 2.3)	0.50	5.00
	P3	**9.1 (9.1; 9.1)**	**15.9 (14.18; 15.9)**	11.4 (11.4; 11.4)	**6.8 (2.2; 6.8) ***	2.3 (0; 2.3)	**0.03**	0.20
	P4	18.15 (13.6; 22.7)	2.25 (0; 21.8)	4.5 (4.5; 31.8)	-15.9 (-22.7; 27.3)	-13.65 (-18.2; 18.2)	0.27	0.30
	**P5**	**25 (20.5; 31.8)**	39.3 (32.7; 44.93)	**50 (50; 50)**	14.3 (0; 25)	**25 (18.2; 29.5) ***	0.06	0.10
	P6	22.7 (11.4; 25)	15.9 (13.6; 19.3)	15.9 (13.6; 19.63)	-6.8 (-11.4; 9.1)	-6.8 (-13.6; 11.3)	0.26	0.40
	P7	20.5 (15.9; 22.7)	18.2 (14.75; 18.2)	22.7 (15.9; 27.9)	-2.3 (-9.1; 2.3)	2.2 (-6.8; 11.4)	0.20	0.40
	P8	50 (50; 52.3)	44.5 (38.1; 53.98)	56.8 (52.3; 63.6)	-5.7 (-15.9; 6.8)	6.8 (0.0; 13.6.0)	0.17	0.20
SURFACE (0-100)	**P1**	**20.5 (17.5; 3)**	8 (3.5; 16.3)	**15.0 (13.5; 16.0)**	-12.5 (-29.0; 0.5)	**-5.5 (-18.5; -1.5)**	0.06	5.00
	**P2**	**6.0 (4.0; 8.0)**	12.0 (7.2; 13.0)	**11.0 (11.0; 15.0)**	6.0 (-2.0; 9.0)	**5.0 (3.0; 11.0) ***	0.09	5.00
	**P3**	**13.0 (12.0; 16.0)**	10.0 (8.2; 11.8)	**5.0 (4.0; 9.0)**	-3.0 (-8.0; 0.0)	**-8.0 (-12.0; -3.0)**	0.06	5.00
	P4	7.0 (4.0; 10.0)	1.5 (0.2; 15.5)	8.0 (0.0; 9.0)	-5.5 (-10.0; 16.0)	1.0 (-10.0; 5.0)	0.27	0.40
	P5	10.0 (10.0; 11.0)	13.0 (9.1; 13.9)	**16.0 (12.0; 20.0)**	3.0 (-3.0; 4.0)	**6.0 (1.0; 10.0)**	0.17	0.10
	P6	25.0 (10.0; 41.0)	12.5 (6.5; 29.8)	11.0 (10.0; 17.0)	-12.0 (-35.0; 24.0)	-14.0 (-31.0; 7.0)	0.20	0.250
	P7	7.0 (7.0; 16.0)	21.8 (17.4; 27.2)	24.0 (12.0; 24.5)	14.8 (0.0; 22.0)	17.0 (-3.5; 17.5)	0.06	0.10
	P8	32.5 (21.0; 45.5)	35.2 (22.8; 39.5)	37.0 (34.0; 40.0)	2.8 (-25.5; 18.5)	4.5 (-11.5; 19.0)	0.40	0.35

*Statistically significant changes indicating worsening of the symptom

Supplementary Table 15. Global analysis of changes in HRQOL by the PedsQL measurement model following haploidentical BM-MSC systemic therapy

SCORES: 0-100			Median (IRQ)			Difference between medians (CI ≈95%)		Wilcoxon test[1]	
								P	
			BASAL	SHORT TERM	LONG TERM	B vs ST	B vs LT	B vs ST	B vs LT
Pediatric Quality of Life Inventory™ (PedsQL™ 4.0[2])	Children	Total Score	47.8 (37.3; 58.4)	52.2 (47.8; 66.3)	56.0 (45.7; 61.7)	3.3 (-0.01; 9.2)	2.18 (-2.28; 15.8)	0.06	0.11
		Physical Health Summary Score	36.0 (25.0; 44.5)	37.5 (28.1; 56.3)	32.8 (20.4; 47.7)	-1.6 (-9.4; 25.0)	2.3 (-15.6; 28.1)	0.42	0.50
		Psychosocial Health Summary Score	54.2 (44.0; 64.2)	60.0 (56.7; 66.7)	61.7 (51.2; 74.8)	6.7 (-10.0; 15.9)	6.7 (-6.7; 19.2)	0.16	0.08
		**Emotional scale score**	**55.0 (42.5; 62.5)**	**65.0 (60.0; 76.0)**	67.5 (48.1; 82.5)	15.0 (-10.0; 17.50)	7.5 (-20.0; 35.0)	**0.05**	0.094
		Social scale score	60.0 (52.5; 66.2)	65.0 (55.0; 70.0)	62.5 (55.0; 76.9)	5.0 (-20.0;10.0)	3.9 (-10.0; 10.0)	0.40	0.31
		School Functioning scale score	45.0 (36.9; 70.0)	60.0 (45.0; 80.0)	60.0 (48.8; 77.5)	10.0 (-5.0; 17.50)	10.0 (0.0; 20.0)	0.06	0.06
	Parents	Total Score	46.3 (40.9; 60.1)	54.0 (38.9; 64.7)	44.5 (34.0; 63.8)	-0.3 (-8.7; 18.4)	-5.2 (-12.5; 13.0)	0.32	0.41
		Physical Health Summary Score	35.9 (17.6; 46.9)	39.1 (19.5; 46.5)	28.1 (3.9; 35.9)	0.0 (-20.3; 28.1)	-10.2 (-36.0; 18.8)	0.5	0.06
		Psychosocial Health Summary Score	56.2 (47.1; 71.2)	60.3 (51.2; 76.7)	53.4 (50.0; 75.9)	2.9 (-5.9; 19.2)	0.03 (-9.2; 19.2)	0.17	0.34
		**Emotional scale score**	**53.8 (44.4; 72.5)**	**65.0 (50.0; 80.0)**	50.0 (40.0; 82.5)	7.5 (-2.5; 20.0)	-1.2 (-12.5; 12.5)	**0.02**	0.47
		Social scale score	58.8 (55.6; 68.1)	52.5 (46.2; 74.4)	56.2 (47.5; 75.0)	-6.2 (-17.5; 30.0)	1.2 (-25.0; 25.0)	0.36	0.50
		School Functioning scale score	56.2 (42.5; 69.4)	59.2 (51.2; 78.8)	60.0 (51.2; 78.8)	8.8 (-12.5; 26.7)	7.5 (-12.5; 26.7)	0.15	0.07
PedsQL™3.0 [3] Multidimensional Fatigue Scale	Children	Total Score	64.9 (57.8; 72.6)	70.8 (59.7; 76.4)	71.9 (63.9; 79.5)	-1.0 (-6.2; 9.7)	8.3 (-0.7; 16.0)	0.5	0.08
		**General fatigue score**	**60.4 (49.5; 70.8)**	**70.8 (58.3; 83.3)**	**66.7 (60.4; 73.4)**	4.1 (-0.01; 14.6)	**6.2 (2.1; 10.4)**	**0.03**	**0.01**
		**Sleep/Rest score**	**58.3 (45.8; 69.3)**	58.3 (50.0; 62.5)	**63.5 (56.2; 79.2)**	-0.01 (-12.5; 20.8)	14.6 (-6.2; 20.8)	0.44	**0.05**
		Cognitive Fatigue score	80.2 (64.1; 85.4)	83.3 (62.5; 100.0)	87.5 (77.1; 98.4)	0.0 (-18.8; 20.8	10.4 (-14.6; 22.9)	0.44	0.16
	Parents	Total Score	63.9 (55.0; 81.4)	68.1 (57.3; 76.7)	64.2 (53.1; 75.4)	2,3 (-12,5; 15,3)	-0.4 (-8.3; 9.7)	0.32	0.42
		General Fatigue score	49.0 (38.0; 71.9)	58.3 (42.7; 75.0)	47.9 (38.5; 68.8)	3,8 (-4,2; 18,8)	-2.1 (-16.7; 10.4)	0.06	0.35
		Sleep/Rest Fatigue	62.5 (51.0; 80.2)	58.3 (45.8; 85.4)	60.4 (47.4; 84.4)	-2,1 (-16,7; 14,6)	2.1 (-14.6; 12.5)	0.42	0.49
		Cognitive Fatigue	84.4 (100.0; 75.0)	81.2 (71.9; 99.0)	87.5 (75.5; 99.0)	0,0 (-29,2; 20,8)	0.0 (-12.5;20.8)	0.50	0.41
PedsQL™ 2.0 [4] Family Impact Module	Parents	Total score	54.3 (46.2; 69.6)	59.4 (47.0; 64.2)	60.4 (54.0; 67.7)	0.8 (-13.9; 9.7)	6.1 (-12.5; 19.4)	0.50	0.18
		The Parent HRQL Summary Score	54.7 (42.4; 69.7)	56.9 (44.7; 69.7)	58.8 (50.9; 78.4)	0.9 (-10.6; 7.5)	3.8 (-10.0; 11.9)	0.41	0.05
		Physical Functioning	46.9 (20.8; 75.5;)	52.1 (18.8; 69.8)	45.8 (32.8; 72.9)	-3.13 (-25.01; 8.33)	-1.1 (-25.0; 27.1)	0.16	0.47
		Emotional Functioning	51.2 (38.8; 62.5	55.0 (42.5; 68.8)	57.5 (49.4; 65.0)	5.0 (-5.0; 22.5)	5.0 (-5.0; 17.5)	0.05	0.06
		Social Functioning	59.4 (50.0; 78.1)	59.4 (37.5; 73.4)	65.6 (45.3; 79.7)	-6.3 (-25.0; 15.62)	1.56 (-18.75; 18.75)	0.22	0.46
		Cognitive Functioning	71.2 (57.5; 75.0)	70.0 (53.8; 80.0)	72.5 (55.0; 91.2)	1.3 (-15; 5)	5.0 (-5.0; 20.0)	0.44	0.09
		Communication	62.5 (46.9; 89.6)	70.8 (60.4; 87.5)	75.0 (60.4; 89.6)	-2.1 (-12.5; 33.3)	4.2 (-4.2; 41.7)	0.29	0.08
		Worry	50.0 (27.5; 60.0)	47.5 (21.2; 65.0)	52.5 (21.9; 68.8)	-3.7 (-25.0; 20.0)	6.3 (-25.0; 17.5)	0.34	0.42
		The Family Functioning Summary Score	61.7 (40.6; 73.1)	62.5 (37.5; 68.0)	58.6 (38.3; 70.2)	0.8 (-25.0; 25.0)	-3.91 (-25.0; 21.87)	0.40	0.16
		Daily Activities	43.8 (33.3; 57.3)	41.7 (27.1; 50.0)	33.3 (30.2; 50.0)	-4.2 (-50.0; 25.0)	-4.2 (-50.0; 25.0)	0.16	0.20
		Family Relationships	68.8 (38.8; 91.2)	72.5 (41.2; 75.0)	72.5 (45.0; 78.8)	-1.2 (-25.0; 25.0)	-3.8 (-20.0; 25.0)	0.31	0.36

[1]One-tailed. [2]The Pediatric Quality of Life Inventory™ version 4.0 (PedsQL™ 4.0) with Generic Core Scales (parent and child forms) assesses four domains of health divided into two Summary Score: Physical Health (physical functioning domain) and Psychosocial Health (emotional, social and school functioning domains). Higher scores indicate better HRQOL. [3] PedsQL™ Multidimensional Fatigue Scale version 3.0 (PedsQL™ 3.0) with parent and child forms (general, sleep/rest fatigue and cognitive fatigue). Higher scores indicate lower problems. [4]PedsQL™ 2.0 Family Impact Module divided into four Summary Score: Parent HRQL (physical, emotional, social and cognitive functioning), Family Functioning (daily activities and family relationships), Communication functioning and Worry. Higher scores indicate better functioning.

Supplementary Table 16. Individual analysis of PedsQL™ 4.0 (parent forms)

Scores: 0-100		Median (IRQ)			Difference between medians (CI ≈95%)		Wilcoxon test (one tailed) P	
		BASAL	SHORT TERM	LONG TERM	B vs ST	B vs LT	B vs ST	B vs LT
Total Score	P1	**45.2 (44.05; 46.4)**	**54.8 (47.6; 61.9)**	38.0 (38.0; 43.5)	**9.5 (1.2; 17.9)**	-8.0 (-8.4; -0.6)	0.17	0.10
	P2	47.3 (33.7; 60.9)	44.6 (42.4; 47.8)	41.3 (35.9; 45.6)	-2.2 (-18.5; 14.1)	-6.0 (-30.4; 13.0)	0.50	0.40
	P3	40.8 (35.9; 45.6)	37.0 (27.4; 39.1)	32.6 (25.0; 35.9)	-3.8 (-18.3; 3.2)	-8.1 (-20.6; 0.0)	0.40	0.20
	P4	39.1 (39.1; 39.1)	53.3 (39.1; 65.2)	47.7 (43.5; 47.8)	(14.1) NA	(8.6) NA	NA	NA
	P5	41.3 (25.0; 57.6)	32.6 (25.0; 38.0)	28.8 (27.2; 30.4)	-8.7 (-32.6; 13.0)	-12.5 (-30.4; 5.4)	0.50	0.50
	P6	63.0 (63.0; 63.0)	65.2 (53.3; 67.4)	63.0 (52.2; 71.7)	(2.2) NA	(0) NA	NA	NA
	P7	68.5 (65.2; 71.7)	63.0 (62.0; 69.6)	64.1 (62.0; 66.3)	-5.4 (-9.8; 4.4)	-4.4 (-9.8; 1.1)	0.20	0.20
	P8	**51.2 (45.2; 57.1)**	**69.6 (67.4; 75.0)**	**64.1 (61.2; 64.1)**	**18.4 (10.2; 29.8)**	**13.0 (4.1; 18.9)**	0.10	0.10
Physical Health Summary Score	P1	36.0 (34.4; 37.5)	45.3 (34.4; 56.2)	15.6 (12.5 (31.2)	9.4 (-3.1; 21.9)	-20.3 (-25.0; -3.1)	0.50	0.10
	P2	50.0 (25.0; 7.05)	31.2 (28.1; 34.4)	25.0 (18.8; 31.2)	-18.8 (-46.9; 9.4)	-25.0 (-56.2; 6.3)	0.50	0.30
	P3	6.2 (6.2; 6.2)	6.2 (3.1; 9.4)	0.0 (0.0; 3.1)	0.0 (-3.1; 3.1)	-6.3 (-6.3; -3.1)	0.50	0.10
	P4	12.5 (12.5; 12.5)	40.6 (21.9; 59.4)	31.2 (28.1; 31.2)	(28.1) NA	(18.8) NA	NA	NA
	P5	36.0 (0.0; 71.9)	15.6 (3.1; 21.9)	0.0 (0.0; 0.0)	-20.3 (-68.8; 21.9)	-35.9 (-71.9; 0.0)	0.50	0.50
	P6	37.5 (37.5; 37.5)	37.5 (21.9; 40.6)	37.5 (25.0; 40.6)	NA	NA	(0) NA	(0) NA
	P7	57.8 (56.2; 59.4)	46.9 (46.9; 59.4)	43.8 (43.8; 46.9)	-10.9 (-12.5; 3.1)	-14.1 (-15.6; -9.4)	0.30	0.10
	P8	**32.8 (28.1; 37.5)**	46.9 (40.6; 53.1)	**31.2 (28.1; 40.6)**	**14.1 (3.1; 25.0)**	-1.6 (-9.4; 12.5)	0.10	0.50
Psychosocial Health Summary Score	P1	**51.0 (48.1; 53.8)**	**60.6 (55.8; 65.4)**	50.0 (50.0; 51.7)	**9.7 (2.0; 17.3)**	-0.95 (-3.8; 3.6)	0.17	0.50
	P2	45.8 (38.3; 53.3)	50.0 (48.3; 58.3)	50.0 (36.7; 55.0)	4.2 (-5.0; 20)	4.2 (-16.6; 16.7)	0.40	0.50
	P3	59.2 (51.7; 66.7)	55.0 (40.4; 55.0)	50.0 (38.3; 53.3)	-4.2 (-26.3; 3.3)	-9.2 (-28.4; 1.6)	0.40	0.20
	P4	53.3 (53.3; 53.3)	60.0 (48.3; 68.3)	56.7 (51.7; 57.1)	(6.7) NA	(3.4) NA	NA	NA
	P5	44.15 (38.3; 50)	38.3 (36.7; 50.0)	44.2 (41.7; 46.7)	-5.85 (-13.3; 11.7)	0.05 (-8.3; 8.4)	0.50	0.50
	P6	76.7 (76.7; 76.7)	78.3 (70.0; 83.3)	76.7 (66.7; 88.3)	(2.0) NA	(0) NA	NA	NA
	P7	**72.5 (60.0; 85.0)**	80.0 (75.0; 85.0)	**85.0 (85.0; 90.0)**	7.5 (-10; 25)	**12.5 (0.0; 30.0)**	0.50	0.50
	P8	**62.5 (55.8; 69.2)**	81.7 (81.7; 86.7)	**81.7 (76.7; 83.3)**	19.2 (12.5; 30.9)	**19.2 (7.5; 27.5)**	0.10	0.10
Emotional scale score	P1	**42.5 (40; 45)**	**50 (50; 50)**	40 (25; 50)	**7.5 (5; 10)**	-2.5 (-20; 0)	0.17	0.30
	P2	52.5 (40; 65)	50 (50; 50)	40 (35; 55)	-2.5 (-15; 10)	-12.5 (-30; 15)	0.50	0.30
	P3	**55 (55; 55)**	**60 (60; 60)**	50 (50; 55)	**5 (5; 5)**	-5 (-5; 5)	0.10	0.30
	P4	50 (50; 50)	70 (55; 70)	50 (50; 55)	(20) NA	(0) NA	NA	NA
	P5	**42.5 (40; 45)**	**40 (35; 60)**	37.5 (30; 45)	**-2.5 (-10; 20)**	-5 (-15; 5)	0.50	0.50
	P6	70 (70; 70)	55 (50; 60)	45 (40; 80)	(-15) NA	(-25) NA	NA	NA
	P7	72,5 (60; 85)	80 (75; 85)	85 (85; 90)	7,5 (-10; 25)	12,5 (0; 30)	0.50	0.30
	P8	72.5 (70; 75)	80 (75; 90)	75 (62.5; 85)	7.5 (0; 20)	2.5 (-12.5; 15)	0.20	0.50
Social scale score	P1	**57.5 (55; 60)**	**72.5 (65; 80)**	60 (60; 70)	**15 (5; 25)**	2.5 (0.0; 15)	0.17	0.30
	P2	45 (40; 50)	45 (45; 70)	55 (40; 60)	5 (-5; 30)	10 (-10; 20)	0.40	0.30
	P3	62.5 (45; 80)	50 (25; 55)	45 (40; 50)	-12.5 (-55; 10)	-17.5 (-40; 5)	0.40	0.30
	P4	60 (60; 60)	50 (40; 60)	55 (50; 62.5)	(-10) NA	(-15) NA	NA	NA
	P5	57.5 (40; 75)	40 (30; 50)	57.5 (45; 70)	-17.5 (-45; 10)	0.0 (-30; 30)	0.30	0.50
	P6	75 (75; 75)	90 (70; 90)	85 (70; 85)	(15) NA	(10) NA	NA	NA
	P7	77.5 (75; 80)	75 (75; 85)	80 (70; 80)	-2.5 (-5; 10)	2.5 (-10; 5)	0.50	0.50
	P8	**55 (45; 65)**	**85 (80; 85)**	**80 (70; 85)**	**30 (15; 40)**	**25 (5; 40)**	0.10	0.10
School Functioning scale score	P1	54.2 (50; 58.3)	58.3 (50; 66.7)	55 (55; 55)	4.2 (-8.3; 16.7)	0.83 (-8.3; 5)	0.50	0.50
	P2	**40 (35; 45)**	**55 (50; 55)**	50.0 (35; 55)	**15 (5; 20)**	10 (-10; 20)	0.10	0.30
	P3	60 (55; 65)	50 (31.2; 55)	60 (15; 60)	-10 (-33.75; 0.0)	0 (-50; 5)	0.20	0.40
	P4	50 (50; 50)	60 (50; 75)	60 (50; 65)	(10) NA	(10) NA	NA	NA
	P5	**32.5 (30; 35)**	**40 (40; 40)**	37.5 (35; 40)	**7.5 (5; 10)**	5 (0; 10)	0.10	0.33
	P6	85 (85; 85)	95 (85; 100)	100 (90; 100)	(10) NA	(15) NA	NA	NA
	P7	72.5 (70; 8)	60 (50; 65)	60 (50; 70)	-12.5 (-25; -5)	-12.5 (-25; 0)	0.10	0.10
	P8	**58.3 (50; 66.7)**	**85 (85; 85)**	**85 (80; 90)**	**26.7 (18.3; 35)**	**26.7 (13.3; 40)**	0.10	0.20

The Pediatric Quality of Life Inventory™ version 4.0 (PedsQL™ 4.0) for parents is made of Generic Core Scales that assesses four domains of health divided into two Summary Score (in grey): Physical Health (physical functioning domain) and Psychosocial Health (emotional, social and school functioning domains). Higher scores indicate better HRQOL.

Supplementary Table 17. Individual analysis of PedsQL™ 4.0 (child forms)

SCORES: 0-100		Median (IRQ)			Difference between medians (CI ≈95%)		Wilcoxon test P	
		BASAL	SHORT TERM	LONG TERM	B vs ST	B vs LT	B vs ST	B vs LT
Total Score	P1	NA	NA	45.6 (41.3; 45.6)	NA	NA	NA	NA
	P2	**39.7 (32.6; 46.7)**	48.9 (46.7; 59.8)	**55.4 (54.4; 63.0)**	0.2 (0.0; 27.2)	**15.8 (7.6; 30.4)**	0.20	0.10
	P3	47.8 (47.8; 47.8)	47.8 (47.8; 53.3)	45.6 (41.3; 50.0)	(0.0) NA	(-2.2) NA	NA	NA
	P4	47.8 (47.8; 47.8)	52.2 (50.0; 59.8)	57.6 (56.5; 63.0)	(4.3) NA	(9.8) NA	NA	NA
	P5	30.4 (22.8; 38.0)	39.1 (34.8; 39.1)	31.0 (27.7; 34.8)	8.7 (-3.3; 16.3)	0.5 (-10.9; 12.0)	0.20	0.50
	P6	56.5 (54.4; 58.7)	56.5 (47.7; 58.7)	56.5 (56.5; 67.4)	0.0 (-11.0; 4.4)	0.0 (-2.2; 13.0)	0.50	0.40
	P7	64.1 (64.1; 64.1)	66.3 (56.5; 66.3)	68.0 (67.4; 68.5)	(2.2) NA	(3.8) NA	NA	NA
	P8	NA	76.1 (71.7; 80.4)	63.0 (56.5; 69.6)	NA	NA	NA	NA
Physical Health Summary Score	P1	NA	NA	25 (25; 37.5)	NA	NA	NA	NA
	P2	**31.3 (31.3; 31.3)**	28.1 (28.1; 40.6)	**37.5 (37.5; 40.6)**	-3.2 (-3.2; 9.3)	**6.2 (6.2; 9.3)**	0.30	0.10
	P3	37.5 (37.5; 37.5)	28.1 (25.0; 37.5)	28.1 (25.0; 37.5)	(-9.4) NA	(-9.4) NA	NA	NA
	P4	34.4 (34.4; 34.4)	37.5 (28.1; 40.6)	40.6 (37.5; 40.6)	(3.1) NA	(6.2) NA	NA	NA
	P5	6.2 (3.1; 9.4)	6.3 (6.3; 15.6)	4.7 (3.1; 6.3)	0.05 (-3.1; 12.5)	-1.6 (-6.3; 3.2)	0.40	0.50
	P6	37.5 (25.0; 5.00)	56.3 (28.6; 56.3)	50.0 (43.8; 62.5)	18.8 (-21.4; 31.3)	12.5 (-6.5; 37.5)	0.20	0.30
	P7	65.6 (65.6; 65.6)	59.4 (43.8; 65.6)	50.0 (50.0; 50.0)	(-6.2) NA	(-15.6) NA	NA	NA
	P8	NA	53.2 (50.0; 56.3)	18.8 (18.8; 31.3)	NA	NA	NA	NA
Psychosocial Health Summary Score	P1	NA	NA	50 (50; 50)	NA	NA	NA	NA
	P2	**44.2 (33.3; 55.0)**	**60.0 (56.7; 70)**	**63.3 (63.3; 76.7)**	**15.9 (1.7; 36.7)**	**19.5 (8.3; 43.4)**	0.10	0.10
	P3	53.3 (53.3; 53.3)	60.0 (58.3; 61.7)	55.0 (51.7; 61.7)	(6.7) NA	(1.7) NA	NA	NA
	P4	55.0 (55.0; 55.0)	61.7 (60.0; 70)	66.7 (66.7; 75)	(6.7) NA	(11.7) NA	NA	NA
	P5	43.3 (33.3; 53.3)	51.7 (50.0; 56.7)	45.0 (40.0; 50.0)	8.4 (-3.3; 23.4)	1.7 (.13.3; 16.7)	0.40	0.50
	P6	66.7 (56.7; 76.7)	56.7 (56.7; 60.0)	60.0 (53.3; 80.0)	-10.0 (-20; 3.3)	-6.7 (-23.4; 23.3)	0.40	0.50
	P7	63.3 (63.3; 63.3)	66.7 (63.3; 70.0)	77.5 (76.7; 78.3)	(6.3) NA	(14.2) NA	NA	NA
	P8	NA	88.3 (83.3; 93.3)	86.7 (76.7; 90.0)	NA	NA	NA	NA
Emotional scale score	P1	NA	NA	40 (20; 50)	NA	NA	NA	NA
	P2	**42.5 (25; 60)**	60 (55; 85)	**75 (75; 85)**	17.5 (-5; 60)	**32.5 (15; 60)**	0.30	0.10
	P3	60 (60; 60)	75 (75; 75)	65 (55; 70)	(15) NA	(5) NA	NA	NA
	P4	60 (60; 60)	75 (80; 65)	70 (70; 75)	(15) NA	(10) NA	NA	NA
	P5	**42.5 (40; 45)**	**50 (50; 50)**	47.5 (45; 50)	**7.5 (5; 10)**	5 (0; 10)	0.10	0.33
	P6	70 (50; 90)	60 (50; 70)	50 (50; 70)	-10 (-40; 20)	-20 (-40; 20)	0.50	0.40
	P7	50 (50; 50)	65 (70; 65)	85 (85; 85)	(15) NA	(35) NA	NA	NA
	P8	NA	85 (80; 90)	100 (80; 100)	NA	NA	NA	NA
Social scale score	P1	NA	NA	70 (40; 70)	NA	NA	NA	NA
	P2	45 (35; 55)	55 (50; 65)	55 (55; 65)	10 (-5; 30)	10 (0; 30)	0.30	0.30
	P3	55 (55; 55)	60 (60; 65)	55 (55; 65)	(5) NA	(0) NA	NA	NA
	P4	65 (65; 65)	70 (60; 75)	75 (70; 80)	(5) NA	(-5)NA	NA	NA
	P5	60 (55; 65)	65 (55; 75)	55 (55; 55)	5 (-10; 20)	-5 (-10; 0)	0.50	0.50
	P6	60 (60; 60)	40 (20; 50)	50 (40; 80)	-20 (-40; -10)	-10 (-20; 20)	0.10	0.40
	P7	70 (70; 70)	65 (55; 80)	77.5 (75; 80)	(-5) NA	(7.5) NA	NA	NA
	P8	NA	90 (80; 100)	80 (80; 80)	NA	NA	NA	NA
School Functioning scale score	P1	NA	NA	60 (60; 60)	NA	NA	NA	NA
	P2	**40 (40; 45)**	**55 (55; 75)**	**60 (60; 80)**	**15 (5; 35)**	**15 (10; 40)**	0.10	0.10
	P3	45 (45; 45)	45 (40; 50)	45 (45; 50)	(0) NA	(0) NA	NA	NA
	P4	40 (40; 40)	50 (45; 55)	60 (55; 70)	(10) NA	(20) NA	NA	NA
	P5	27.5 (0.0; 55)	45 (40; 45)	32.5 (15; 50)	17.5 (-15; 45)	5 (-40; 50)	0.50	0.50
	P6	70 (60; 80)	80 (70; 80)	90 (60; 90)	10 (-10; 20)	20 (-20; 30)	0.40	0.30
	P7	70 (70; 70)	65 (65; 70)	70 (70; 70)	(-5) NA	(0)NA	NA	NA
	P8	NA	90 (90; 90)	80 (70; 90)	NA	NA	NA	NA

The Pediatric Quality of Life Inventory™ version 4.0 (PedsQL™ 4.0) for children is calculated from Generic Core Scales that assesses four domains of health divided into two Summary Score (in grey): Physical Health (physical functioning domain) and Psychosocial Health (emotional, social and school functioning domains). Higher scores indicate better HRQOL.

Supplementary Table 18. Individual analysis of PedsQL™ Multidimensional Fatigue Scale (parent forms)

SCORES: 0-100		Median (IRQ)			Difference between medians (CI ≈95%)		Wilcoxon test	
							P	
		BASAL	SHORT TERM	LONG TERM	B vs ST	B vs LT	B vs ST	B vs LT
Total Score	P1	54.2 (45.8; 62.5)	63.9 (61.1; 72.2)	58.3 (54.2; 61.1)	9.7 (-1.4; 26.4)	4.2 (-8.3; 15.3)	0.20	0.50
	P2	**41.7 (40.3; 43.1)**	**56.9 (48.6; 59.7)**	51.4 (33.3; 55.6)	**15.3 (5.5; 19.4)**	9.7 (-9.7; 15.3)	0.10	0.40
	P3	62.5 (62.5; 62.5)	50.0 (23.6; 76.4)	60.3 (56.9; 66.7)	(-12.5) NA	(-2.2) NA	NA	NA
	P4	65.3 (65.3; 65.3)	73.6 (65.3; 81.9)	68.1 (65.3; 72.2)	(8.3) NA	(2.8) NA	NA	NA
	P5	57.6 (55.6; 59.7)	58.3 (45.8; 68.1)	49.3 (45.8; 52.8)	0.7 (-13.9; 12.5)	-8.3 (-13.9; -2.8)	0.50	0.17
	P6	91.7 (91.7; 91.7)	95.6 (84.7; 95.8)	93.1 (88.9; 93.1)	(3.92 NA	(1.4) NA	NA	NA
	P7	79.9 (73.6; 86.1)	72.2 (65.3; 81.9)	72.2 (70.8; 76.4)	-7.6 (-20.8; 8.3)	-7.6 (-15.3; 2.8)	0.20	0.20
	P8	82.0 (70.8; 93.1)	77.8 (73.6; 86.1)	76.4 (76.4; 83.3)	-4.2 (-19.4; 15.3)	-5.6 (-16.7; 12.5)	0.50	0.50
General Fatigue score	P1	**43.8 (37.5; 50.0)**	**62.5 (58.3; 70.8)**	50.0 (45.8; 58.3)	**18.4 (8.3; 33.3)**	6.2 (-4.2; 20.8)	0.10	0.30
	P2	27.1 (20.8; 33.3)	45.8 (33.3; 50.0)	37.5 (29.7; 45.8)	18.8 (0.0; 29.2)	10.4 (-4.2; 25.0)	0.20	0.20
	P3	37.5 (37.5; 37.5)	37.5 (20.8; 45.8)	41.7 (33.3; 50.0)	(0.0) NA	(4.2) NA	NA	NA
	P4	54.2 (54.2; 54.2)	58.3 (41.7; 70.8)	50.0 (37.5; 54.2)	(4,2) NA	(-4.2) NA	NA	NA
	P5	39.6 (37.5; 41.7)	41.7 (29.3; 62.5)	27.1 (0.0; 54.2)	2.1 (-12.5; 25.0)	-12.5 (-41.7; 16.7)	0.50	0.50
	P6	91.6 (91.7; 91.7)	95.0 (79.2; 95.8)	87.5 (83.3; 87.5)	(3.4) NA	(-4.1) NA	NA	NA
	P7	62.5 (41.7; 83.3)	58.3 (33.3; 70.8)	45.8 (45.8; 62.5)	-4.2 (-50.0; 29.2)	-16.7 (-37.5; 20.8)	0.40	0.50
	P8	75.0 (66.7; 83.3)	79.2 (70.8; 91.7)	75.0 (70.8; 79.2)	4.2 (-12.5; 25.0)	0.0 (-12.5; 12.5)	0.40	0.50
Sleep/Rest Fatigue	P1	31.2 (50.0 ; 12.5)	45.8 (37.5; 54.2)	37.5 (29.2; 50.0)	14.6 (-12.5; 41.7)	6.2 (-20.8; 37.5)	0.40	0.50
	P2	54.2 (41.7; 66.7)	62.5 (62.5; 70.8)	58.3 (37.5; 66.7)	8.3 (-4.2; 29.2)	4.2 (-29.2; 25.0)	0.40	0.50
	P3	50.0 (50.0; 50.0)	41.7 (16.7; 87.5)	62.5 (54.2; 62.5)	(-8.3) NA	(12.5) NA	NA	NA
	P4	66.7 (66.7; 66.7)	66.7 (66.7; 75.0)	62.5 (58.3; 66.7)	(0.0) NA	(-4.2) NA	NA	NA
	P5	58.3 (54.2; 62.5)	45.8 (37.5; 66.7)	43.8 (29.2; 58.3)	-12.5 (-25.0; 12.5)	-14.6 (-33.3; 4.2)	0.40	0.33
	P6	83.3 (83.3; 83.3)	91.7 (75.0; 91.7)	91.7 (83.3; 91.7)	(8.3) NA	(8.3) NA	NA	NA
	P7	95.8 (91.7; 100)	91.7 (87.5; 95.8)	95.8 (91.7; 95.8)	-4.2 (-12.5; 4.2)	-0.0 (-8.3; 4.2)	0.30	0.50
	P8	70.8 (45.8; 95.8)	54.2 (54.2; 66.7)	58.3 (58.3; 70.3)	-16.7 (-41.7; 20.8)	-12.5 (-37.5; 25.0)	0.50	0.59
Cognitive Fatigue	P1	87.5 (87.5; 87.5)	87.5 (83.3; 91.7)	87.5 (75.0; 87.5)	0.0 (-4.2; 4.2)	0.0 (-12.5; 0.0)	0.50	0.60
	P2	43.8 (33.3; 54.2)	58.3 (50; 62.5)	54.2 (33.3; 58.3)	14.6 (-4.2; 29.2)	10.4 (-20.8; 25.0)	0.20	0.50
	P3	100 (100; 100)	70.8 (33.3; 95.8)	87.5 (75.0; 90.0)	(-29.2) NA	(-12.5) NA	NA	NA
	P4	75 (75; 75)	95.8 (87.5; 100)	95.8 (95.8; 95.8)	(20.8) NA	(20.8) NA	NA	NA
	P5	75 (75; 75)	75.0 (70.8; 87.5)	77.1 (75.0; 79.2)	0.0 (-4.7; 12.5)	2.1 (0.0; 4.2)	0.50	0.50
	P6	100 (100; 100)	100 (100; 100)	100 (100; 100)	(0.0) NA	(0.0) NA	NA	NA
	P7	81.2 (75.0; 87.5)	75 (66.7; 79.2)	75.0 (70.8; 75.0)	-6.2 (-20.8; 4.1)	-6.2 (-16.7; 0.0)	0.30	0.30
	P8	100 (95.8; 100)	100 (95.9; 100)	100 (95.8; 100)	0.0 (-4.2; 0.0)	0.0 (-4.2; 0.0)	0.60	0.60

Total score in PedsQL™ Multidimensional Fatigue Scale, version 3.0 (PedsQL™ 3.0) for parent forms is calculated from general, sleep/rest fatigue and cognitive fatigue with higher scores indicate lower problems.

Supplementary Table 19. Individual analysis of PedsQL™ Multidimensional Fatigue Scale (child forms)

SCORES 0-100		Median (IRQ)			Difference between medians (CI ≈95%)		Wilcoxon test	
							P	
		BASAL	SHORT TERM	LONG TERM	B vs ST	B vs LT	B vs ST	B vs LT
Total Score	P1	NA	NA	63.9 (61.1; 66.7)	NA	NA	NA	NA
	P2	**47.9 (44.4; 51.4)**	47.2 (45.8; 58.3)	**63.9 (58.3; 65.3)**	-0.7 (-5.6; 13.9)	**16.0 (6.9; 20.8)**	0.40	0.10
	P3	68.8 (66.7; 70.8)	62.5 (58.3; 63.9)	68.1 (56.9; 70.8)	-6.2 (-12.5; -2.8)	-0.7 (-13.9; 4.2)	0.10	0.50
	P4	61.1 (61.1; 61.1)	70.8 (56.9; 73.6)	75.7 (70.8; 80.6)	(9.7) NA	(14.6) NA	NA	NA
	P5	61.1 (58.3; 63.9)	59.7 (56.9; 65.8)	60.4 (56.9; 63.9)	-1.4 (-6.9; 6.9)	-0.7 (-7.0; 5.6)	0.50	0.50
	P6	**77.8 (77.8; 77.8)**	75.0 (69.4; 80.6)	**88.9 (88.9; 94.4)**	-2.8 (-8.3; 2.8)	**11.1 (11.1; 16.7)**	0.40	0.10
	P7	70.8 (56.9; 84.7)	76.4 (75; 88.9)	76.4 (72.2; 84.7)	5.6 (-9.7; 32.0)	5.6 (-12.5; 27.8)	0.40	0.50
	P8	NA	80.6 (61.1; 83.3)	80.6 (72.2; 80.6)	NA	NA	NA	NA
General fatigue score	P1	NA	NA	66.7 (58.3; 66.7)	NA	NA	NA	NA
	P2	54.2 (54.2; 54.2)	58.3 (45.8; 62.5)	58.3 (54.2; 75.0)	4.2 (-8.3; 8.3)	4.2 (0.0; 20.8)	0.40	0.30
	P3	56.2 (54.2; 58.3)	58.3 (58.3; 62.5)	66.7 (54.2; 66.7)	2.1 (0.0; 8.3)	10.4 (-4.2; 12.5)	0.30	0.30
	P4	66.7 (66.7;66.7)	70.8; 50.0; 70.8)	68.8 (66.7; 70.8)	(4.2) NA	(2.1) NA	NA	NA
	P5	35.4 (25.0; 45.8)	45.8 (41.7; 50.0)	43.8 (41.7; 45.8)	10.4 (-4.2; 25.0)	8.3 (-4.2; 20.8)	0.30	0.50
	P6	83.3 (75.0; 91.7)	83.3 (75.0; 100.0)	91.7 (91.7; 91.7)	-0.0 (-16.7; 25.0)	8.3 (0.0; 16.7)	0.50	0.40
	P7	64.6 (50.0; 79.2)	79.2 (66.7; 83.3)	66.7 (62.5; 75.0)	14.6 (-12.5; 33.3)	2.1 (-16.7; 25.0)	0.30	0.50
	P8	NA	83.3 (58.3; 91.7)	75.0 (75.0; 75.0)	NA	NA	NA	NA
Sleep/Rest score	P1	NA	NA	41.7 (25.0; 50.0)	NA	NA	NA	NA
	P2	**45.8 (41.7; 50.0)**	45.8 (41.7; 50.0)	**62.5 (54.2; 62.5)**	-0.0 (-8.3; 8.3)	**16.7 (4.7; 20.8)**	0.50	0.10
	P3	68.8 (66.7; 70.8)	62.5 (54.2; 70.8)	62.5 (54.2; 70.8)	-6.2 (-16.6; 4.2)	-6.2 (-16.7; 4.2)	0.30	0.30
	P4	45.8 (45.8; 45.8)	58.3 (45.8; 66.6)	64.6 (58.3; 70.8)	(12.5) NA	(18.8) NA	NA	NA
	P5	50.0 (50.0; 50.0)	50.0 (50.0; 54.2)	54.2 (50.0; 58.3)	0.0 (0.0; 4.2)	4.2 (0.0; 8.3)	0.60	0.50
	P6	70.8 (58.3; 83.3)	58.3 (50.0; 58.3)	83.3 (75.0; 91.7)	-12.5 (-33.3; 0.0)	12.5 (-8.3; 33.3)	0.30	0.30
	P7	66.7 (41.7; 91.7)	87.5 (75.0; 100.0)	87.5 (79.2; 87.5)	20.8 (-16.7; 58.3)	20.8 (-12.5; 45.8)	0.40	0.50
	P8	NA	58.3 (41.7; 58.3)	66.7 (41.7; 66.7)	NA	NA	NA	NA
Cognitive Fatigue score	P1	NA	NA	91.7 (83.3; 91.7)	NA	NA	NA	NA
	P2	**43.8 (37.5; 62.5)**	50.0 (37.5; 62.5)	**62.5 (58.3; 75.0)**	6.2 (-12.5; 25.0)	**18.8 (8.3; 37.5)**	0.50	0.10
	P3	81.2 (75.0; 87.5)	62.5 (62.5; 62.5)	75.0 (62.5; 75.0)	-18.8 (-25;.0 -12.5)	-6.2 (-25.0; 0.0)	0.10	0.30
	P4	70.8 (70.8; 70.8)	83.3 (75.0; 83.3)	93.8 (87.5; 100)	(12.5) NA	(22.9) NA	NA	NA
	P5	97.9 (95.8; 100.0)	87.5 (75.0; 91.7)	83.3 (75.0; 91.7)	-10.4 (-25.0; -4.2)	-14.6 (-25.0; -4.2)	0.10	0.17
	P6	79.2 (58.3; 100.0)	100 (50; 100)	100 (91.7; 100)	20.8 (-50.0; 41.7)	20.8 (-8.3; 41.7)	0.50	0.40
	P7	81.2 (79.2; 83.3)	75.0 (70.8; 83.3)	83.3 (66.7; 91.7)	-6.2 (-12.5; 4.2)	2.1 (-16.7; 12.5)	0.30	0.50
	P8	NA	100 (100; 83.3)	100 (100; 100)	NA	NA	NA	NA

Total score in PedsQL™ Multidimensional Fatigue Scale version 3.0 (PedsQL™ 3.0) for children forms is calculated from general, sleep/rest fatigue and cognitive fatigue scores with higher scores indicate lower problems.

Supplementary Table 20. Individual analysis of PedsQL™ 2.0 Family Impact Module (parent forms)

SCORES: 0-100 %		Median (IRQ)			Difference between medians (CI ≈95%)		Wilcoxon test (one tailed) P	
		BASAL	SHORT TERM	LONG TERM	B vs ST	B vs LT	B vs ST	B vs LT
Total score	P1	47.2 (45.1; 49.3)	45.8 (45.1; 59.7)	57.6 (42.4; 63.2)	-1.4 (-4.2; 14.6)	10.4 (-6.9; 18.1)	0.50	0.40
	P2	68.1 (68.1; 68.1)	65.3 (59.0; 68.8)	64.6 (59.7; 65.3)	(-2.8) NA	(-3.5) NA	NA	NA
	P3	38.9 (38.9; 38.9)	25.7 (25.0; 25.7)	26.4 (7.6; 35.4)	(-13.2) NA	(-12.5) NA	NA	NA
	P4	50.7 (50.7; 50.7)	60.4 (54.2; 63.9)	57.6 (53.5; 57.6)	(9.7) NA	(7) NA	NA	NA
	P5	45.8 (43.8; 47.9)	50.7 (42.4; 50.7)	52.8 (45.1; 60.4)	4.9 (-5.6; 7.0)	7.0 (-2.8; 16.7)	0.30	0.33
	P6	72.2 (70.1; 74.3)	58.3 (57.6; 62.5)	68.8 (64.6; 70.1)	-13.9 (-16.7; -7.6)	-3.5 (-9.7; 0.0)	0.10	0.20
	P7	58.0 (50.7; 65.3)	61.0 (56.2; 70.1)	63.2 (59.7; 70.8)	3.1 (-9.0; 19.5)	5.2 (-5.6; 20.1)	0.40	0.40
	P8	70.1 (66.7; 73.6)	75.0 (72.9; 76.4)	78.5 (75.0; 81.9)	4.8 (-0.7; 9.8)	8.3 (1.4; 15.3)	0.20	0.10
The Parent HRQL Summary Score	P1	49.4 (47.5; 51.3)	46.3 (43.8; 66.3)	56.3 (42.5; 68.8)	-3.13 (-7.5; 18.75)	6.9 (-8.8; 21.2)	0.40	0.40
	P2	68.8 (68.8; 68.8)	68.8 (63.8; 77.5)	70.0 (66.5; 72.5)	(0.0) NA	(1.2) NA	NA	NA
	P3	40 (40; 40)	30.0 (23.8; 35.0)	30.0 (11.2; 48.8)	(-10.0) NA	(-10.0) NA	NA	NA
	P4	60 (60; 60)	67.5 (62.5; 70.0)	61.3 (57.5; 62.5)	(7.5) NA	(1.3) NA	NA	NA
	P5	41.9 (37.5; 46.3)	43.8 (35.0; 51.3)	53.8 (40; 67.5)	1.9 (-11.3; 13.8)	11.9 (-6.3; 30.0)	0.50	0.33
	P6	80.6 (80; 81.3)	70.0 (66.3; 71.5)	81.3 (76.2; 82.5)	-10.6 (-15; -8.8)	0.63 (-5; 2.5)	0.10	0.50
	P7	43.8 (35; 52.5)	45.8 (43.8; 62.5)	50.0 (41.3; 62.5)	2.1 (-8.8; 27.5)	6.3 (-11.3; 27.5)	0.40	0.40
	P8	**70.0 (65.0; 75.0)**	75.0 (73.8; 77.5)	**81.3 (76.3; 87.5)**	5.0 (-1.3; 12.5)	**11.9 (1.3; 22.5)**	0.30	0.10
Physical Functioning	P1	43.8 (41.7; 45.8)	45.8 (29.2; 58.3)	45.83 (41.7; 66.7)	2.1 (-16.7; 16.7)	2.1 (-4.1; 25.0)	0.50	0.50
	P2	79.2 (79.2; 79.2)	66.7 (62.5; 70.8)	66.7 (62.5; 70.8)	(-12.5) NA	(-12.5) NA	NA	NA
	P3	33.3 (33.3; 33.3)	25.0 (8.3; 29.2)	8.3 (4.2; 45.8)	(8.3) NA	(-25.0) NA	NA	NA
	P4	50.0 (50.0; 50.0)	58.3 (50.0; 58.3)	45.83 (29.2; 54.2)	(8.3) NA	(-4.2) NA	NA	NA
	P5	**16.7 (8.3; 25.0)**	16.7 (12.5; 41.7)	**43.8 (29.2; 58.3)**	0.0 (-12.5; 33.3)	**27.1 (4.2; 50)**	0.40	0.17
	P6	95.8 (91.7; 100)	70.8 (70.8; 75.0)	87.5 (79.2; 91.7)	-25.0 (-29.2; -16.7)	-8.3 (-20.8; 0.0)	0.10	0.20
	P7	14.6 (4.2; 25.0)	8.3 (8.3; 12.5)	29.2 (25.0; 33.3)	-6.3 (-16.7; 8.3)	14.6 (0.0; 29.2)	0.50	0.20
	P8	**64.6 (62.5 ; 66.7)**	**70.8 (66.7; 85.0)**	**75.0 (75.0; 83.3)**	**6.3 (0.0; 22.5)**	**10.4 (8.3; 20.8)**	0.20	0.10
Emotional Functioning	P1	37 (35; 40)	40 (20; 70)	55 (25; 60)	2.5 (-20; 35)	17.5 (-15; 25)	0.50	0.40
	P2	50 (50; 50)	60 (50; 65)	65 (55; 75)	(10) NA	(15) NA	NA	NA
	P3	25 (25; 25)	30 (25; 45)	25 (0; 50)	(5) NA	(0) NA	NA	NA
	P4	65 (65; 65)	75 (65; 80)	65 (65; 70)	(10) NA	(0) NA	NA	NA
	P5	52.5 (50; 55)	50 (50; 60)	47.5 (45; 50)	-2.5 (-5; 10)	-5 (-10; 0)	0.50	0.33
	P6	55 (55; 55)	50 (50; 50)	60 (50; 60)	-5 (-5; 0)	-5 (-5; 5)	0.30	0.30
	P7	42.5 (25; 60)	65 (60; 100)	55 (30; 75)	22.5 (0; 75)	12.5 (-30; 50)	0.20	0.40
	P8	65 (50; 80)	70 (55; 80)	70 (60; 85)	5 (-25; 30)	5 (-20; 35)	0.50	0.40
Social Functioning	P1	50.0 (50.0; 50.0)	56.2 (43.8; 62.5)	43.75 (37.5; 75)	6.2 (-6.2; 12.5)	-6.2 (-12.5; 25.0)	0.40	0.40
	P2	68.8 (68.8; 68.8)	62.5 (62.5; 81.2)	75.0 (62.5; 93.8)	(-6.2) NA	(6.2) NA	NA	NA
	P3	50.0 (50.0; 50.0)	25.0 (25.0; 43.8)	31.2 (18.8; 50.0)	(-25.0) NA	(-18.8) NA	NA	NA
	P4	50.0 (50.0; 50.0)	56.2 (56.25; 62.5)	50.0 (50.0; 56.2)	(6.2) NA	(0.0) NA	NA	NA
	P5	37.5 (31.2; 43.8)	31.2 (25.0; 43.8)	56.2 (37.5; 75.0)	-6.2 (-18.8; 12.5)	18.8 (-6.2; 43.8)	0.50	0.33
	P6	78.1 (68.8; 87.5)	68.8 (62.5; 81.2)	81.2 (75.0; 87.5)	-9.4 (-25.0; 12.5)	3.1 (-12.5; 18.8)	0.30	0.50
	P7	87.5 (97.5; 87.5)	75.0 (68.8; 81.2)	75.0 (75.0; 87.5)	-12.5 (-18.8; -6.5)	-12.5 (-12.5; 0.0)	0.10	0.30
	P8	**78.1 (75.0; 81.2)**	**93.8 (87.5; 93.8)**	**93.8 (87.5; 93.8)**	**15.6 (6.3; 18.8)**	**15.6 (6.2; 18.8)**	0.10	0.10
Cognitive Functioning	P1	67.5 (65; 70)	65 (65; 75)	75 (65; 80)	-2.5 (-5; 10)	7.5 (-5; 15)	0.50	0.30
	P2	75 (75; 75)	80 (80; 100)	70 (65; 75)	(5) NA	(-5) NA	NA	NA
	P3	55 (55; 55)	40 (25; 40)	50 (25; 60)	(-15) NA	(-5) NA	NA	NA
	P4	75 (75; 75)	80 (75; 85)	80 (75; 90)	(5) NA	(0) NA	NA	NA

	P5	65 (65; 65)	70 (55; 70)	70 (50; 90)	5 (-10; 5)	5 (-15; 25)	0.30	0.50
	P6	90 (90; 90)	80 (75; 90)	95 (90; 100)	-10 (-15; 0)	5 (0; 10)	0.30	0.30
	P7	45 (40; 50)	50 (50; 70)	50 (45; 65)	5 (0; 30)	5 (-5; 25)	0.30	0.30
	P8	75 (70; 80)	70 (65; 80)	95 (70; 100)	-5 (-15; 10)	20 (-10; 30)	0.50	0.30
Communication	P1	70.8 (66.7; 75.0)	58.3 (58.3; 66.7)	66.7 (41.7; 75.0)	-12.5 (-16.7; 0.0)	-4.17 (-33.33; 8.33)	0.20	0.50
	P2	33.3 (33.3; 33.3)	66.7 (58.3; 66.7)	75.0 (58.3; 75.0)	(33.3) NA	(41.7) NA	NA	NA
	P3	50.0 (50.0; 50.0)	75.0 (58.3; 83.3)	50.0 (16.7; 58.3)	(25.0) NA	(0.0) NA	NA	NA
	P4	58.3 (58.3; 58.3)	75.0 (58.3; 83.3)	83.3 (75.0; 83.3)	(16.7) NA	(25.0) NA	NA	NA
	P5	45.8 (33.3; 58.3)	41.7 (33.3; 7.05)	58.3 (41.7; 75.0)	-4.1 (-25; 41.67)	12.5 (-16.7; 41.7)	0.50	0.33
	P6	66.7 (50.0; 83.3)	66.7 (58.3; 66.7)	75.0 (58.3 ; 83.3)	0.0 (-25.0; 16.7)	8.4 (-25.0; 33.3)	0.50	0.50
	P7	100 (100; 100)	91.7 (91.7; 100)	100 (91.7; 100)	-8.3 (-8.3; 0.0)	0.0 (-8.3; 0.0)	0.30	0.60
	P8	95.8 (91.7; 100)	91.7 (91.7; 91.7)	91.7 (91.7; 91.7)	-4.1 (-8.3; 0.0)	-4.1 (-8.3; 0.0)	0.40	0.40
Worry	P1	**45 (40; 50)**	**65 (55; 65)**	**60 (55; 70)**	**20 (5; 25)**	**15 (5; 30)**	0.10	0.10
	P2	55 (55; 55)	45 (40; 45)	45 (30; 50)	(-10) NA	(-10) NA	NA	NA
	P3	25 (25; 25)	0 (0; 0)	0 (0; 0)	(-25) NA	(-25) NA	NA	NA
	P4	25 (25; 25)	15 (15; 25)	15 (15; 15)	(-10) NA	(-10) NA	NA	NA
	P5	**35 (35; 35)**	**40 (40;45)**	**42.5 (40; 45)**	**5 (5; 10)**	**7.5 (5; 10)**	0.10	0.17
	P6	60 (50; 70)	50 (45; 55)	65 (40; 75)	-10 (-25; 5)	5 (-30; 25)	0.30	0.50
	P7	62.5 (60; 65)	65 (60; 75)	80 (80; 85)	2.5 (-5; 15)	17.5 (15; 25)	0.50	0.10
	P8	**60 (60; 60)**	**70 (65; 75)**	70 (60; 70)	**10 (5; 15)**	10 (0; 10)	0.10	0.30
The Family Functioning Summary Score	P1	34.4 (34.4; 34.4)	37.5 (25.0; 40.63)	40.6 (34.4; 56.2)	3.1 (-9.4; 6.2)	6.2 (0.0; 21.9)	0.40	0.30
	P2	87.5 (87.5; 87.5)	62.5 (59.4; 68.8)	65.6 (43.8; 65.6)	(-25.0) NA	(-21.9)NA	NA	
	P3	40.6 (40.6; 40.6)	15.6 (0.0; 25.0)	15.6 (0.0; 25.0)	(-25.0) NA	(-25.0) NA	NA	NA
	P4	40.6 (40.6; 40.6)	65.6 (56.2; 65.6)	62.5 (59.9; 65.6)	(25.0) NA	(21.9) NA	NA	NA
	P5	62.5 (56.2; 68.8)	62.5 (59.4; 65.6)	54.7 (46.9; 62.5)	0.0 (-9.37; 9.38)	-7.8 (-21.9; 6.2)	0.50	0.33
	P6	60.9 (59.9; 62.5)	37.5 (37.5; 43.8)	37.5 (34.9; 50)	-23.4 (-25.0; -15.6)	-23.4 (-28.1; -9.4)	0.10	0.10
	P7	75.0 (65.6; 84.4)	78.1 (71.9; 78.1)	75.0 (71.9; 78.1)	0.0 (-12.5; 12.5)	-0.0 (-12.5; 12.5)	0.50	0.50
	P8	67.2 (65.6; 68.8)	68.8 (65.6; 8)	71.9 (71.9; 8)	1.6 (-3.1; 9.4)	4.7 (3.1; 9.4)	0.50	0.10
Daily Activities	P1	33.3 (33.3; 33.3)	25 (25; 41.67)	33.3 (25.0; 41.7)	-8.3 (-8.3; 8.4)	0.0 (-8.3; 8.3)	0.50	0.30
	P2	75.0 (75.0; 75.0)	50.0 (41.7; 66.7)	50.0 (33.3; 50.0)	(-25.0) NA	(-25.0) NA	NA	NA
	P3	50.0 (50.0; 50.0)	0.0 (0.0; 8.3)	0.0 (0.0; 25.0)	(-50.0) NA	(-50.0) NA	NA	NA
	P4	25.0 (25.0; 25.0)	50.0 (33.3; 50.0)	50.0 (25.0; 50.0)	(-25.0) NA	(-25.0) NA	NA	NA
	P5	33.3 (33.3; 33.3)	33.3 (33.3; 41.7)	29.7 (8.3; 50.0)	0.0 (0.0; 8.3)	-4.2 (-25.0; 16.7)	0.60	0.50
	P6	54.2 (50.0; 58.3)	41.7 (25.0; 41.7)	33.3 (16.7; 50.0)	-12.5 (-33.3; -8.3)	-20.8 (-41.7; 0.0)	0.10	0.20
	P7	37.5 (16.7; 58.3)	41.7 (25.0; 41.7)	33.3 (25.0; 50.0)	4.2 (-33.3; 2.0)	-4.2 (-33.3; 33.3)	0.50	0.50
	P8	58.3 (50.0; 66.7)	58.3 (58.3; 58.3)	66.7 (58.3; 66.7)	-0.0 (-8.3; 8.3)	8.3 (-8.3; 16.7)	0.50	0.40
Family Relationships	P1	**35 (35; 35)**	40 (25; 45)	**45 (40; 65)**	5 (-10; 10)	**10 (5; 30)**	0.4	0.1
	P2	95 (95; 95)	70 (70; 70)	75 (40; 85)	NA	NA	-25 (NA)	-20(NA)
	P3	35 (35; 35)	25 (0.00; 35)	25 (0.00; 25)	NA	NA	-10 (NA)	-10(NA)
	P4	50 (50; 50)	75 (70; 75)	75 (70; 80)	NA	NA	25 (NA)	25(NA)
	P5	80 (70; 90)	75 (75; 85)	70 (70; 70)	-5 (-15; 15)	-10 (-20; 0)	0.5	0.5
	P6	**65 (60; 70)**	**45 (35; 45)**	45 (40; 50)	**-25 (-35; -15)**	-20 (-30; -10)	0.1	0.1
	P7	97.5 (95; 100)	100 (100; 100)	100 (95; 100)	2.5 (0; 5)	2.5 (-5; 5)	0.4	0.7
	P8	72.5 (70; 75)	75 (70; 85)	80 (75; 80)	2.5 (-5; 15)	7.5 (0; 10)	0.5	0.2

Total score in PedsQLTM 2.0 Family Impact Module is divided into four Summary Scores (in grey): Parent HRQL (physical, emotional, social and cognitive functioning), Family Functioning (daily activities and family relationships), Communication functioning and worry. Higher scores indicate better functioning.

frontiers

Supplementary Table 21. Overview of clinical trials using mesenchymal stromal cells and subpopulations for the treatment of RDEB

CT IDENTIFIER	DESIGN	SOURCE	N	REGIME	DOSE CELLS/KG	FOLLOW-UP	SITE	GLOBAL EFICACY OUTCOMES	REF
**EBSTEM**<br>**EUDRACT 2012-00894-87**	Phase I/II	BM-MSC unrelated	10 RDEB children	3 IV injections (D0,7,28)	1-3 x10^6	1 year	UK	↓ BEBS, skin erythema & blister counts<br>↑ wound healing,<br>↑ QoL<br>Benefits up to 4-6 months	(3)
**Case Series report**	Phase I/II	BM-MSC parents	14 RDEB children	IV injections	2 x10^6	1 year	Egypt	↑ wound healing<br>↓ New blister<br>↑AF<br>Benefits up to 6 months & 1 year in 2 patients	(4)
**ADSTEM**<br>**NCT02323789**	Phase I/II,<br>single group, open-label	BM-MSC unrelated	10 RDEB adults	2 IV injections (D0,14)	2-4 x10^6	1 year	UK	↓ disease activity scores<br>↓ itch<br>Benefits up to 4-6 months	(5)
**MesensistemEB**<br>**NCT04153630**	**Phase I/II,<br>single group, open-label**	**BM-MSC haploidentic**	**8 RDEB children**	**3 IV injections (D0,21, 42)**	**2-3 x10^6**	**1 year**	**Spain**	**↓ itch frequency<br>↑ C7 in 2 patients (20-30% vs control)<br>Responses correlated with sCD40L & MCP1 dynamics<br>↑ Monocyte CLA expression<br>↑Memory CD8$^+$ T cells & ↓CD62L$^-$ TEMRA-like cells<br>Disappearance of aberrant granulocytes<br>↑ QoL (fatigue & rest/sleep)<br>Parents and children emotional relief<br>Variable transient benefits up to 1 year**	**This paper**
**Case report**	Proof of concept	Ad-MSC	1 RDEB adult	3 IV injections (D0,21, 42)	1 x10^6	2 years	Spain	↓ EBDASI, BSA, itch, pain<br>↑QoL	(6)
**NCT03529877;**<br>**EudraCT 2018-001009-98**	Phase I/IIa, multicentric, single-arm,	ABCB5+ dermal unrelated (RHEACELL)	16 RDEB children and adults	3 IV injections (D0,17, 35)	2 x10^6	12 weeks (efficacy)<br>1 year (safety)	Germany<br>Austria<br>France Italy<br>USA<br>UK<br>Australia	↓ EBDASI activity score, scorEB-c score (12 weeks)<br>↓ itch and pain (35 days)	(7)
**NCT04520022**	Phase I/II<br>single-group, open-label	hUCB-MSCs unrelated	6 RDEB children and adults	3 IV injections (D0,14,28)	1-3 x10^6	8 mo-2 years	South Korea	↓ BEBS, BSA & blister counts<br>↓ itch and pain<br>↑ QoL<br>Maximal benefits 56–112 days after treatment	(8)
**MissionEB**<br>**ISRCTN14409785**	Phase I dose de-escalation, randomised, placebo controlled, double blinded, crossover (3 mo)<br>Open label non-randomised (1 y)	hUCB-MSCs unrelated (CORDStrom)	34 RDEB children	Repeated (D0, W2, M4, M4+2W, M8, M8+2W)	2-3 x10^6 to 1-1,5x10^6	15 months	UK	EBDASI: No significant improvement (3 months)<br>↓ Itch most in RDEB-Severe<br>↓ Pain most in >10-year-olds (6 months)<br>↓Disease activity mainly <10-year-olds & RDEB-Intermediate<br>10/13 blinded participants reported benefit from UC-MSCs	(9, 10)
JapicCTI-184563	Phase 1/2 open-label, non-randomized, single-arm, non-controlled	CL2020 BM-MSC Allogeneic multilineage-differentiating stress-enduring (Muse) cells	5 DEB patients with refractory ulcers and recurrent ulcers lasting for more than 4 weeks	Single IV infusion	1.98-3.55 x10^6 (2.98 ± 0.61 × 10^5)	52 weeks	Japan	Ulcer↓ (>50% in 2 patients)<br>↓ ulcer size (significant at Wk04)<br>Moderate pain improvement<br>Improved liver function in 2 patients<br>Temporary benefit (ulcers returned by Wk12)	(11)
